# EmmaEmb: A quantitative framework for analyzing embedding spaces in molecular biology

**DOI:** 10.1016/j.patter.2026.101635

**Published:** 2026-08-12

**Authors:** Pia Francesca Rissom, Vít Škrhák, Paulo Yanez Sarmiento, Jordan F. Safer, Connor W. Coley, Bernhard Y. Renard, Henrike O. Heyne, Sumaiya Iqbal

**Affiliations:** 1 Hasso Plattner Institute, Digital Engineering Faculty, University of Potsdam, Potsdam, Germany; 2 Bioinformatics and Machine Learning, Broad Institute of MIT and Harvard, Cambridge, MA, USA; 3 Department of Software Engineering, Charles University, Prague, Czech Republic; 4 Faculty of Biology, Technion-Israel Institute of Technology, Haifa, Israel; 5 Department of Chemical Engineering, Massachusetts Institute of Technology, Cambridge, MA, USA; 6 Department of Electrical Engineering, Massachusetts Institute of Technology, Cambridge, MA, USA; 7 Hasso Plattner Institute for Digital Health at Mount Sinai, Icahn School of Medicine, New York, NY, USA; 8 Finnish Institute for Molecular Medicine (FIMM), University of Helsinki, Helsinki, Finland; 9 Program in Medical and Population Genetics, Broad Institute of MIT and Harvard, Cambridge, MA, USA; 10 Cancer Data Science, Dana-Farber/Harvard Cancer Center, Cambridge, MA, USA

**Keywords:** embedding spaces, foundation models, protein language models, molecular biology, vector space analysis, embedding interpretability, embedding comparison, transfer learning, error detection, pairwise comparison

## Abstract

Embeddings, numerical vectors learned by deep-learning models, are increasingly used to represent complex molecular biology data and support predictive tasks and generative design. There is a growing need for systematic approaches to interpret and explain the information encoded in high-dimensional embedding spaces. Here, we introduce EmmaEmb, a quantitative, model-agnostic framework for geometric correction, direct analysis, and comparison of embedding spaces. Our framework encompasses local and global analysis methods to quantify data distribution within an embedding space and enable comparisons of representations across spaces in relation to known biological features. Through experiments with seven embedding models across six molecular biology tasks, we demonstrate that our methods reveal insights from embedding spaces that align with downstream predictive tasks, uncover misclassification patterns, and contextualize differences in biological information captured by ProtT5, AlphaFold2, and ESM C. We provide an open-source Python library implementing all analysis methods and a guided diagnostic workflow.

## Highlights

• EmmaEmb is a computational framework to analyze and compare biological embedding spaces
• Embedding analyses rank protein model representations by task relevance without training
• Embedding-space neighborhoods reveal misclassification patterns in transfer learning
• EmmaEmb quantifies representational changes induced by model fine-tuning

## The bigger picture

Foundation models are large AI models that learn from the structure and patterns of large datasets without being trained for a specific task. They are increasingly trained on biological molecules and processes and can then be used to solve biological problems, such as predicting protein function or advancing drug discovery. This works because these models convert complex data into lists of numbers, called embeddings. Each embedding represents a protein, gene, or cell as a point in a high-dimensional space. Much of what a model learns is captured in the geometry of this embedding space: which samples lie close together, which lie far apart, and how biological categories are arranged. To assess what an embedding space has captured, or which one is best suited to a particular question, researchers today typically train additional predictive models using the foundation model’s embeddings. This gives an informative answer for one specific task. However, it becomes computationally costly to scale across many tasks and embedding spaces and reveals little about how the embedding space is organized overall.

EmmaEmb offers a complementary approach. Instead of evaluating one task at a time, it examines how biological samples are arranged within a foundation model’s embedding space as a whole. Using information researchers already have, such as functional annotations, taxonomic labels, or clinical metadata, it measures how different biological categories are represented and enables comparison between models. It also flags technical artifacts (so-called geometric distortions), that can mislead such comparisons. Although demonstrated here using protein models, we hope the EmmaEmb framework presented in this study will find use across other biological data, including gene expression, multi-omics, or cell imaging. As foundation models spread across biology, tools that help researchers understand and compare what they have learned will be increasingly valuable before investing in more resource-intensive training and evaluation.

## Teaser

Foundation models generate embeddings that capture complex biological information, but interpreting and comparing these representations remains challenging. Rissom et al. introduce EmmaEmb, a model-agnostic framework for the analysis of embedding spaces. Applied to protein language models and high-stakes molecular biology and drug-discovery tasks, EmmaEmb ranks representations by task relevance, reveals patterns underlying prediction errors, and tracks representational changes during model adaptation, providing a quantitative approach to understanding learned biological representations. All methods are available as an open-source Python library.

## Introduction

With the growing availability of data and computing power, foundation models have become increasingly relevant in molecular biology for encoding high-dimensional, multi-modal data. These models are trained to capture inherent relationships within large datasets, uncovering complex patterns and placing individual data points into a broader biological context. Protein language models (PLMs), for example, are a type of foundation model based on large language model architectures, trained on extensive protein sequence data from diverse species.^1^^,^^2^^,^^3^^,^^4^ PLMs capture rich, biologically relevant, contextual information about protein structure, function, and evolution. Reportedly, PLMs are transforming many molecular and protein biology tasks, from structure and function prediction^3^^,^^4^^,^^5^^,^^6^^,^^7^ to protein design and engineering.^8^^,^^9^

Challenges exist, however, in interpreting the information learned by foundation models due to their “black-box” nature, hidden biases, and dataset-specific artifacts.^10^ Moreover, the proliferation of diverse architectures (e.g., layers and parameters) and training regimes (e.g., data modalities and training objectives) of foundation models has made it challenging to systematically assess which models are most suitable for specific biological questions. Such explainability and interpretability of foundation models are especially crucial in high-stakes applications in molecular biology and drug discovery.^11^^,^^12^^,^^13^ As foundation models become tools for biological discovery, there is a pressing need for methods that can elucidate information captured by these models and guide their applications in a principled manner.

Foundation models encode the learned information from input data in numerical vectors, known as embeddings (i.e., latent spaces), and are therefore also referred to as embedding models. It has been demonstrated that embeddings are powerful in downstream prediction tasks^14^^,^^15^^,^^16^ and as representational spaces for generative design.^9^^,^^17^^,^^18^ However, because these vectors are high-dimensional, the encoded information is difficult to interpret directly. Probing embedding spaces offers a window into model learning, shedding light on how the model internally represents biological data. Current strategies for comparing embedding spaces of multiple models usually leverage machine learning (ML), such as training task-specific ML classifiers using embeddings to evaluate their predictive power^6^ and correlating the embeddings with biological features to identify where specific feature information is encoded.^19^^,^^20^ While effective, these approaches require additional training and are resource intensive.

A complementary method is to analyze the embedding spaces directly: in-space feature distribution and cross-space comparison. In natural language processing (NLP), the direct analysis of embeddings has revealed semantic relationships and biases within the data,^21^^,^^22^ and comparison of embedding spaces has enabled knowledge transfer from high-resource to low-resource languages.^23^ In molecular biology, the analysis of single embedding spaces has reportedly enhanced the understanding of continuous prediction tasks,^24^^,^^25^ multi-modal data integration,^26^ informed model uncertainty,^27^ and improved interpretability by visualizing embeddings.^28^ However, comparative analysis across embedding spaces remains underexplored in molecular biology. Although tools for embedding-space comparison exist (Table 1), their application to molecular biology remains limited, as most tools rely on visual inspection, providing few quantitative measures. Moreover, existing tools focus on individual data points, which are rarely meaningful in isolation for genes or proteins, where biological insight often emerges from patterns across functional groups.

**Table 1:** Comparison of published tools for analysis of multiple embedding spaces

Tool name	Dimensionality reduction techniques for visualization^a^	Feature distribution: visualization	Feature distribution: quantification	Feature distribution: neighbors	Space comparison: visualization	Space comparison: global analysis	Space comparison: neighborhood	Space comparison: feature integration	Code available	Tool description
EmmaEmb	PCA, t-SNE, UMAP	✔	✔	✔	✔	✔	✔	✔	✔	the framework and library introduced in this paper
repcomp^29^	–	–	–	–	–	✔	✔	–	✔	computation of similarities between two embedding spaces; nearest neighbors, canonical correlation, unit match; no visualization of results
embComp^30^	IsoMap, LLE, MDS, PCA, t-SNE, UMAP	–	–	–	✔	–	✔	–	–	visual local neighborhood analysis, based on KNN; distribution of similarities; cosine or Euclidean distance
Embedding comparator^31^	PCA, t-SNE, UMAP	–	–	–	✔	–	✔	–	✔	visual local neighborhood analysis, based on KNN; distribution of similarities; highlighting least similar samples and their nearest neighbors; cosine or Euclidean distance
Vectory^32^	PCA, UMAP	–	–	–	✔	✔	–	–	✔	aggregated similarity of local neighborhoods based on KNN; cosine or Euclidean distance
Emblaze^33^	PCA, t-SNE, UMAP	✔	–	–	✔	–	✔	–	✔	visual local neighborhood analysis, based on KNN; visualizing trajectories of points between embedding spaces; highlighting nearest neighbors of individual embeddings and user-selected sets of embeddings; cosine or Euclidean distance
EmbCompare^34^	PCA	✔	–	–	✔	✔	✔	–	✔	visual local neighborhood analysis, based on KNN; distribution of similarities; highlighting least similar samples; cosine distance
Latentverse^35^	PCA	✔	✔	–	✔	–	–	✔	✔	evaluation of latent representations, by assessing clustering, disentanglement, generalization, expressiveness, and robustness

a PCA, principal-component analysis; t-SNE, t-stochastic neighbor embedding; UMAP, uniform manifold approximation and projection; IsoMap (Tenenbaum et al.^36^); LLE, local linear embedding (Roweis and Saul^37^); MDS, multi-dimensional scaling (Kruskal^38^).

To bridge this gap, we present a quantitative, model-agnostic framework, EmmaEmb (Embedding, Metadata, and Multimodel Analyzer for Exploration in Molecular Biology), for direct analysis and comparison of embedding spaces in molecular biology. We propose methods to analyze the distribution of data (i.e., biological features or metadata) within an embedding space and compare multiple embedding spaces using local neighborhood analyses and global distance metrics. We illustrate the utility of EmmaEmb through a series of experiments on PLMs, including embedding-space ranking for downstream tasks, interpreting misclassification patterns, and providing insights into how different model architectures encode data. Furthermore, we implemented a suite of diagnostic tools in EmmaEmb to uncover and adjust for geometric properties of embedding spaces (e.g., anisotropy and hubness) and to assess the robustness of conclusions with regard to data biases such as feature noise and class label imbalance. While we focus on molecular biology applications in this paper, our framework is generalizable to other data modalities. We publish a Python library that implements all components of EmmaEmb for open accessibility and reproducibility (https://github.com/broadinstitute/EmmaEmb).

In the next section, we detail the EmmaEmb framework and its analysis methods. We also describe the datasets and embeddings used in the subsequent results section to benchmark EmmaEmb in different applications. We end with the discussion, where we provide a summary along with further insights from the experiments, the limitations of EmmaEmb, and future directions.

## Methods

This section describes the EmmaEmb framework and its analysis methods, metrics, and visualization functions. Additionally, the datasets and embeddings utilized to validate EmmaEmb and its Python implementation are detailed.

### EmmaEmb framework

**Figure 1:**
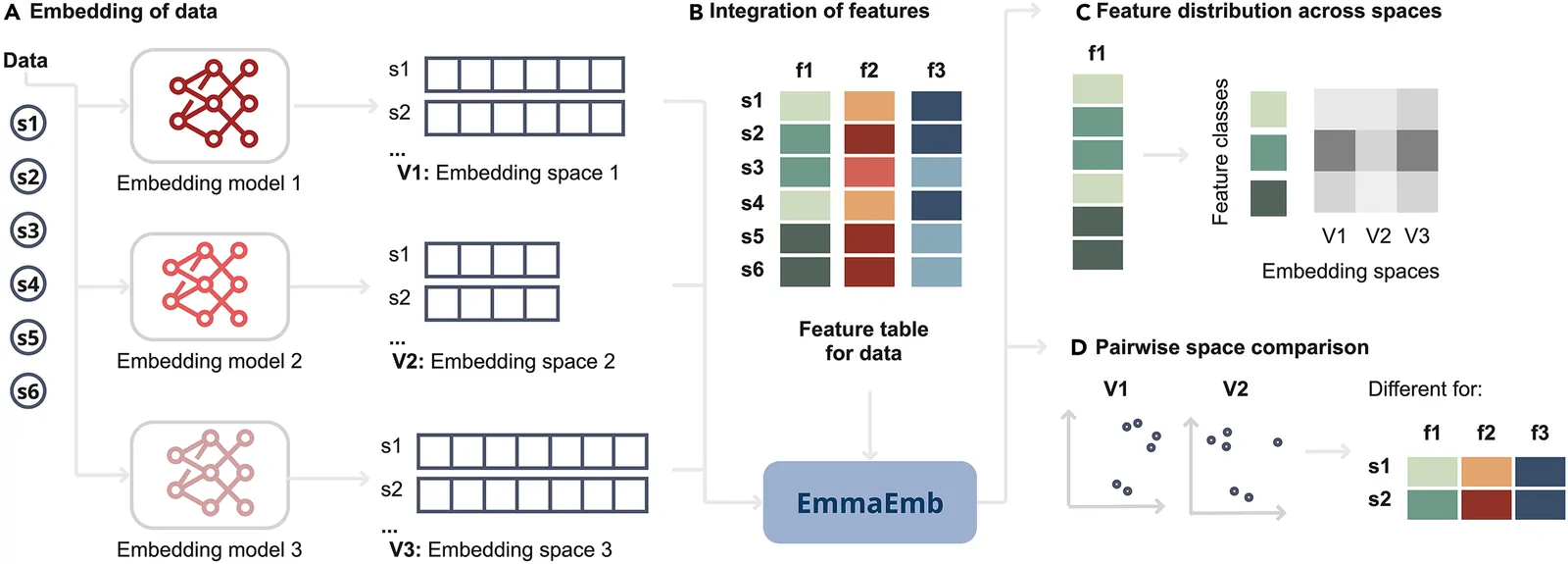
Schematic of the EmmaEmb framework and proposed workflow (A) Starting with a set of data samples (e.g., genes or proteins, here *s*_1_–*s*_6_), embeddings are derived from different embedding models (here, embedding models *V*_1_–*V*_3_). For protein biology applications, embeddings can be derived from protein language models that vary in their architecture or training. (B) Categorical features (here, *f*_1_–*f*_3_) associated with the samples are integrated into the analysis. (C) Analysis approach 1. Feature distribution across spaces focuses on examining how samples with specific features, such as protein families, are grouped within the neighborhoods within an embedding space as well as across spaces. Multiple *k*-nearest-neighbor (KNN)-based methods can be applied within this approach to quantify the proximity of samples with the same feature as well as the proximity of groups of samples with the same feature. (D) Analysis approach 2. Pairwise space comparison involves the identification of similarities and differences in distances between embeddings or KNNs between two embedding spaces. Regions of notable difference can be further analyzed using the features to characterize samples that are represented differently across the models. Methods in (C) and (D) are modular and can be applied in any order, either independently within each approach or in combination between the approaches, depending on the analysis goals.

Figure 1 provides an overview of EmmaEmb and the proposed workflow. We begin by generating embeddings of data samples (Figure 1A), denoted as $s_{1},\ldots ,s_{N_{S}}$, where *N*_*S*_ is the total number of samples (e.g., proteins or genes). Embeddings of all samples from an embedding model are referred to as an embedding space, denoted by *V*. The *h*th model takes each sample *s*_*i*_ and maps it onto its corresponding embedding space $V_{h}\in R^{d_{h}}$, producing the vector representation of embedding, ${\;v}_{i}^{\left(h\right)}$, where *d*_*h*_ is the dimensionality of the embedding vectors for model *h*, and *N*_*V*_ is the total number of embedding spaces compared.

(Equation 1) $$\begin{array}{c} s_{i}⟼v_{i}^{\left(h\right)}\in V_{h},\forall \;i=1,\ldots ,N_{S},h=1,\ldots ,N_{V},N_{V}\geq 2\; \end{array}\text{.}$$

Next, we integrate features (Figure 1B) of samples into EmmaEmb to analyze the information captured in the embedding spaces. The feature vectors can include biological groupings as metadata, such as protein classes, experimental annotations, or functional labels. Each feature vector of length *N*_*S*_ is denoted by f_*p*_, where *p* = 1,…,*N*_*f*_, and *N*_*f*_ is the total number of feature vectors. We denote the value of the *p*th feature for sample *s*_*i*_ as f_p_[*i*].

Finally, EmmaEmb includes two complementary analysis approaches. The first approach, “feature distribution across spaces,” examines how specific biological features are represented within and across embedding spaces (Figure 1C). The second approach, “pairwise space comparison,” quantifies differences between embedding spaces directly (Figure 1D). The methods are modular and can be applied independently or in combination, depending on the task.

In both analysis approaches, we compare pairwise distances between samples across embedding spaces. For example, the distance between embeddings of samples *s*_*i*_ and *s*_*j*_ in space *V*_*h*_ is dist$\left(v_{i}^{\left(h\right)},v_{j}^{\left(h\right)}\right),h=1,\ldots ,N_{V}$. Because pairwise distances between samples are intrinsic properties of the embedding space that do not depend on dimensionality or axis alignment, they provide a coordinate-free basis for comparing how different models represent the same set of samples. These analyses assume that distances between embedding spaces are meaningful, that is, that the embedding space does not exhibit strong geometric biases such as anisotropy or hubness that can distort distance-based comparisons. Guidance on verifying and correcting for these properties is provided in the guided diagnostic workflow (see “EmmaEmb Python library” and Note S1). EmmaEmb considers multiple distance metrics, such as Euclidean, cosine, or Manhattan distances, capturing complementary aspects of proximity in high-dimensional spaces.^39^

The proposed analysis approaches can be performed on the local and global structures of samples in an embedding space. The local structure is the geometric relationship between each sample and its immediate neighbors in the embedding space. In contrast, the global structure represents the overall sample distribution and organization within the full space. For local structure analysis, we consider a subset of pairwise distances between each sample and its *k*-nearest neighbors (KNNs) within an embedding space. For a sample *s*_*i*_ in embedding space *V*_*h*_, this neighborhood is denoted as $N_{s_{i}}^{\left(h\right)}$. Following prior works,^27^^,^^40^ we adapt KNN-based metrics and introduce several scores based on embedding neighborhoods within the EmmaEmb framework (Table 2; detailed below). We suggest analyzing embeddings and datasets using multiple distance metrics and a range of *k* values to capture local structures robustly and at different scales.

**Table 2:** Overview of EmmaEmb’s neighborhood-based formulas

Formula name	Formula	Description
KNN feature alignment score	$\text{Alig}n_{p}^{\left(h\right)}\left(s_{i}\right)≔\frac{1}{k}\left|\left\{s_{j}\in N_{s_{i}}^{\left(h\right)}:f_{p}\left[j\right]=f_{p}\left[i\right]\right\}\right|$	measures, for sample *s*_*i*_, how well a feature is preserved within its local neighborhood $N_{s_{i}}^{\left(h\right)}$ in embedding space *h*. Here, *f* denotes a feature vector over all samples, *f*_*p*_[*i*] the value of the *p*th feature for sample *s*_*i*_, and *s*_*j*_ ranges over all *k*-nearest neighbors of *s*_*i*_ in $N_{s_{i}}^{\left(h\right)}$. The score lies in [0,1], with 1 indicating that all *k* neighbors share the same feature value as *s_i_*
KNN class mixing matrix	$M_{a,b}^{\left(h,p\right)}≔\sum_{s_{i}\in S_{c_{a}}}\left|\left\{s_{j}\in N_{s_{i}}^{\left(h\right)}:f_{p}\left[j\right]=c_{b}\right\}\right|$	summarizes how samples from class *c*_*a*_ are locally connected to samples from class *c*_*b*_ in embedding space *h*. For each sample *s*_*i*_ with class label *f*_*p*_[*i*] = *c*_*a*_, the formula counts neighbors $s_{j}\in N_{s_{i}}^{\left(h\right)}$ whose feature value *f*_*p*_[*j*] equals *c*_*b*_. The full matrix *M*^(*h*,*p*)^ captures cross-class neighborhood patterns, providing a structured overview of how biological classes relate to one another in embedding space *h*
KNN class ranking matrix	$R_{a,b}^{\left(h,p\right)}≔\text{rank}{\left(M_{a,\cdot }^{\left(h,p\right)}\right)}_{b}$	derived from the KNN class mixing matrix *M*^(*h*,*p*)^. For each query class *c*_*a*_, neighbor classes *c*_*b*_ are ranked according to their frequency in $M_{a,b}^{\left(h,p\right)}$, with rank 1 assigned to the most frequent neighbor class. The entries of *R*^(*h*,*p*)^ range from 1 to $\left|C_{p}\right|$, providing an order-based view of class mixing patterns in embedding space *h*
Cross-space neighborhood similarity	$\text{Si}m^{\left(h,h^{'}\right)}\left(s_{i}\right)≔\frac{\left|N_{s_{i}}^{\left(h\right)}\cap N_{s_{i}}^{\left(h^{'}\right)}\right|}{k}$	quantifies how similar the local neighborhoods of sample *s*_*i*_ are across embedding spaces *h* and *h*′. The measure counts how many of the *k* nearest neighbors of *s*_*i*_ appear in both $N_{s_{i}}^{\left(h\right)}$ and $N_{s_{i}}^{\left(h^{'}\right)}$. The score lies in [0,1], where 1 indicates identical neighborhoods and 0 indicates no shared neighbors

#### EmmaEmb analysis approach, 1: Feature distribution across spaces

**Figure 2:**
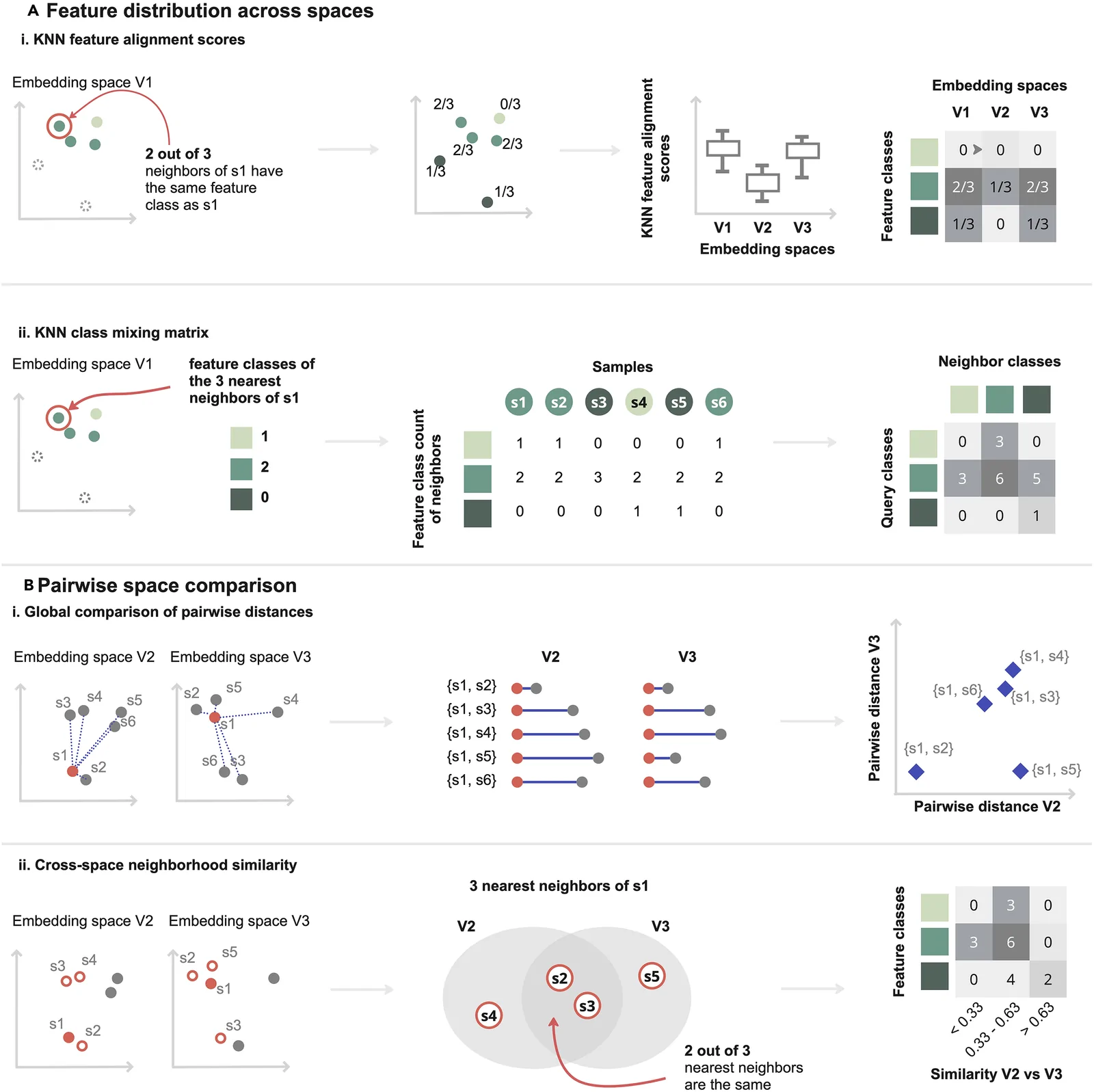
Graphical illustration of embedding-space analysis and comparison methods in EmmaEmb (A) For the analysis of the feature distribution of a specific feature (here, feature *f*_1_) for a set of samples (here, *s*_1_–*s*_6_) across embedding spaces (here, features *V*_1_–*V*_3_), we propose two methods. (i) KNN feature alignment scores. The KNN feature alignment scores are calculated for each embedding within its embedding space, as a fraction of its *k*-nearest-neighboring embeddings that share the same class label as the embedding itself. By comparing the distribution of KNN feature alignment scores across different embedding spaces, we can assess how closely embeddings of the same *f*_1_ class are to other embeddings of the same class. The score provides insights into how well an embedding model has learned to group embeddings with similar characteristics. (ii) KNN class mixing matrix. The matrix is calculated separately for each embedding space (here, for *V*_1_). For each embedding, the class distribution of class labels of its *k*-nearest neighbors is computed. The class distributions are then summed for all embeddings of the same class. The resulting matrix shows, for each class, the number of *k*-nearest neighbors of embeddings from this class for another class. This allows insights into which classes are close to each other, and which are more differentiated within the embedding space. (B) We also propose methods for pairwise embedding-space comparison. (i) Global comparison of pairwise distances (here, between embedding spaces *V*_2_ and *V*_3_). For this method, we first calculate all pairwise distances between embeddings within each embedding space. We then compare the correlation of pairwise distances, e.g., distance between {*s*_1_, *s*_2_}, across the two embedding spaces. This method provides an overview of the global similarity between embedding spaces and reveals groups of samples that are differently represented. (ii) Cross-space neighborhood similarity, in contrast, quantifies local differences between two embedding spaces. It is calculated for each sample as the number of *k*-nearest neighbors shared between *V*_2_ and *V*_3_. Identifying and characterizing samples with high divergence in their local neighborhood can help to uncover structural differences between the embedding spaces.

This analysis approach enables the quantitative evaluation of how a selected feature (e.g., the functional class for a protein or gene; Figure 2A) is distributed within the neighborhoods of an embedding space. We propose the following two KNN-based methods and scores for quantitative assessments (Table 2).

##### KNN feature alignment score

The KNN feature alignment score quantifies how closely related a sample is to its embedding neighbors with respect to the feature *f*_*p*_.

For sample *s*_*i*_, the KNN feature alignment score in embedding space *V*_*h*_ is the proportion of its neighbors $N_{s_{i}}^{\left(h\right)}$ with the same value of *f*_*p*_:

(Equation 2) $$\begin{array}{c} \text{Alig}n_{p}^{\left(h\right)}\left(s_{i}\right)≔\frac{1}{k}\left|\left\{s_{j}\in N_{s_{i}}^{\left(h\right)}:f_{p}\left[j\right]=f_{p}\left[i\right]\right\}\right| \end{array}\text{.}$$

The score ranges from 0 to 1, with higher values indicating a greater representation of the feature class in the sample’s neighborhood.

KNN feature alignment scores can be grouped by feature labels to examine how each feature class is captured in an embedding space. Furthermore, KNN feature alignment scores of one embedding space can be directly compared to those from another embedding space, enabling an assessment of how well different models capture relationships for the same feature.

##### KNN class matrix

To gain a broader view on how different feature classes relate to one another in an embedding space, we analyze the distribution of neighbors across classes. Specifically, for each embedding space *V*_*h*_, we define a “KNN class mixing matrix” that captures how frequent samples from one class have neighbors from another class.

Let $C_{p}=\left\{c_{1},\ldots ,c_{\left|C_{p}\right|}\right\}$ denote the set of $\left|C_{p}\right|$ unique categorical values (classes) of a feature *f*_*p*_. For each class $c_{a}\in C_{p},S_{c_{a}}=\left\{s_{i}:f_{p}\left[i\right]=c_{a}\right\}$ is the set of all samples belonging to class *c*_*a*_. For a specific embedding space *V*_*h*_ and each pair of classes $\left(c_{a},c_{b}\right)\in C_{p}\times C_{p}$, we count the number of neighbors of samples in class *c*_*a*_ that belong to class *c*_*b*_ to compute the KNN class mixing matrix, $M^{\left(h,p\right)}\in {\mathbb{N}}^{\left|C_{p}\right|\times \left|C_{p}\right|}$.

(Equation 3) $$\begin{array}{c} M_{a,b}^{\left(h,p\right)}≔\sum_{s_{i}\in S_{c_{a}}}\left|\left\{s_{j}\in N_{s_{i}}^{\left(h\right)}:f_{p}\left[j\right]=c_{b}\right\}\right| \end{array}\text{.}$$

A value of 0 indicates that none of the *k*-nearest neighbors of any sample in class *c*_*a*_ belong to class *c*_*b*_, indicating that these two classes are separated within the embedding space according to the target feature. In contrast, the maximum value of $k\cdot N_{c_{a}}$ corresponds to when all neighbors of all samples in class *c*_*a*_ belong exclusively to class *c*_*b*_. The KNN class mixing matrix, therefore, captures the extent of neighborhood proximity between classes. However, high neighborhood proximity between two classes does not necessarily imply a single large cluster; it could indicate the presence of multiple distinct subclusters within or between those classes.

To obtain a simplified summary of the proximity relationships, we further define a KNN class ranking matrix, $R^{\left(h,p\right)}\in {\mathbb{N}}^{\left|C_{p}\right|\times \left|C_{p}\right|}$, derived from *M*^(*h*,*p*)^. For each query class *c*_*a*_, the *a*th row of *R*^(*h*,*p*)^ contains the rank indices of the neighbor classes sorted in descending order of *M*^(*h*,*p*)^. In other words, $R_{a,b}^{\left(h,p\right)}=r$ means that class *c*_*b*_ is the *r*th most frequent neighbor class for query class *c*_*a*_. The entries of *R*^(*h*,*p*)^ therefore range from 1 to $\left|C_{p}\right|$, with a value of 1 corresponding to the most frequent neighbor class and $\left|C_{p}\right|$ to the least frequent.

#### EmmaEmb analysis approach, 2: Pairwise space comparison

Here, we compare two embedding spaces directly by analyzing differences in distances between samples or neighborhood structures and use feature information to interpret divergent regions (Figure 2B).

##### Global comparison of pairwise distances

To perform a global comparison of pairwise distances across embeddings, we compute Spearman’s rank correlation coefficient (*ρ*) of pairwise distance values. This statistical measure allows us to evaluate how well the pairwise relationships between embeddings are preserved across different embedding spaces by comparing their ranking across different spaces. The pairwise distances in the two embedding spaces can be visualized in a scatterplot.

##### Cross-space neighborhood similarity

To quantify and analyze local differences between two embedding spaces *V*_*h*_ and $V_{h^{\prime }}$, we first identify the *k*-nearest-neighbor sets $N_{s_{i}}^{\left(h\right)}$ and $N_{s_{i}}^{\left(h^{\prime }\right)}$ previously defined for each sample *s*_*i*_. Following prior works,^30^^,^^31^^,^^33^^,^^34^ we define the neighborhood similarity score for sample *s*_*i*_ as the fraction of overlapping neighbors between the two embedding spaces:

(Equation 4) $$\begin{array}{c} \text{Si}m^{\left(h,h^{\prime }\right)}\left(s_{i}\right)≔\frac{\left|N_{s_{i}}^{\left(h\right)}\cap N_{s_{i}}^{\left(h^{\prime }\right)}\right|}{k} \end{array}\text{.}$$

This score ranges from 0 (no shared neighbors) to 1 (identical neighborhoods), capturing how well local neighborhood structures align across embedding spaces.

In addition to quantifying neighborhood overlap, EmmaEmb includes methods to analyze how the shared (i.e., overlapping) neighbors are distributed across classes in relation to a categorical feature *f*_*p*_, offering insights into whether neighborhood similarity reflects meaningful biological groupings.

### EmmaEmb Python library

We implemented EmmaEmb as a lightweight, modular Python library,^41^ centered around the Emma object that handles embedding spaces, feature data, and intermediate computational results. Multiple functions on the Emma object enable analysis of feature distributions across embedding spaces, including feature alignment and neighborhood analysis (Figure 2A), as well as pairwise space comparisons between different embedding spaces (Figure 2B). EmmaEmb integrates functionalities from established libraries such as scikit-learn^42^ and Plotly^43^ to calculate the scores (Table 2) and offer intuitive visualizing functions. For distance measurement, EmmaEmb uses a variety of metrics, including Manhattan, Euclidean, and cosine distances, leveraging standard implementations from scikit-learn.^42^ To ensure scalability for very large datasets, we provide the option to approximate neighborhood searches using the ANNOY algorithm,^44^ which allows efficient identification of approximate nearest neighbors in high-dimensional spaces.

#### Geometric diagnostics and mean centering

EmmaEmb’s local and global methods assume that distances between embeddings are meaningful. This assumption can be violated when embedding spaces exhibit anisotropy or hubness. Anisotropy refers to the tendency of embedding vectors to cluster in a narrow directional cone rather than spreading uniformly, which inflates cosine similarity between all pairs and reduces the discriminative power of distance metrics.^45^ Hubness refers to the phenomenon whereby a small number of points become the nearest neighbors of a disproportionately large fraction of other points—a mathematical consequence of high dimensionality that makes KNN-based scores unreliable.^46^ To allow users to empirically verify these assumptions before proceeding, EmmaEmb includes geometric diagnostic functions covering anisotropy (average cosine similarity and per-dimension variance, following Ethayarajh^45^) and hubness (*k*-occurrence distribution skewness and Robin Hood index [RHI], following Radovanovic et al.^46^ and Feldbauer et al.^47^). Where anisotropy is detected, EmmaEmb provides a function to apply mean centering, subtracting the mean embedding vector from all embeddings, as a correction prior to all subsequent analysis steps.

#### Metadata requirements and feature integration

EmmaEmb is primarily designed for categorical metadata, although continuous features can be incorporated via binning or used directly for visual exploration after dimensionality reduction. Neighborhood-based scores require at least *k* samples per group to be interpretable, and EmmaEmb automatically flags when this constraint is violated. Further, EmmaEmb provides missing data flagging when loading a feature dataframe, with columns exceeding a configurable threshold of missing values flagged before analysis proceeds.

#### Guided diagnostic workflow for embedding-space ranking

To support systematic and reproducible analysis of how well different embedding spaces encode task-relevant biological features, EmmaEmb provides a six-step guided diagnostic workflow (see Note S1 for details) that structures the analysis from geometric verification through to robustness analysis. The geometric diagnostics in steps 1 and 2 characterize properties of the embedding space itself and are recommended prior to any EmmaEmb analysis, including pairwise space comparison. Steps 3 through 6 are specific to feature distribution analysis and the ranking of embedding spaces by their alignment with a given biological feature and are run separately for each feature of interest.

Step 1. Anisotropy assessment: computes average cosine similarity on raw and mean-centered embeddings and per-dimension variance to detect and correct for geometric directional bias; determines the recommended distance metric and whether mean centering should be applied
Step 2. Hubness assessment: computes the *k*-occurrence distribution and RHI on both raw and mean-centered embeddings (evaluated at neighborhood sizes *k* = 5, 10, 20); the severity of hubness informs the ceiling on the neighborhood size *k* used in the analysis steps 3–6
Step 3. Class distribution and neighborhood size range: determines the appropriate range of neighborhood sizes (*k*) for analysis in steps 4–6, based on class sizes, dataset size, and hubness severity from step 2; computes the expected random baseline alignment score per class; flags missing data and class imbalance
Step 4. Parameter sensitivity analysis: evaluates ranking stability of mean KNN feature alignment scores across the determined *k* range and selected distance metrics, producing a ranking stability plot
Step 5. Class imbalance robustness (run if flagged in step 3): progressively downsamples the majority class and recomputes mean KNN feature alignment scores at each subsampling ratio, assessing whether embedding-space rankings are stable across different class balance conditions
Step 6. Feature noise robustness: progressively permutes a fraction of annotations while keeping embeddings fixed, recomputing mean KNN feature alignment scores at each noise level to assess whether embedding-space rankings are robust to annotation uncertainty

Based on the diagnostic results, the workflow provides guided recommendations for distance metric selection and *k* range, ensuring that parameter choices are appropriate for the embedding spaces and datasets analyzed. A step-by-step walkthrough example can be accessed as an interactive Google Colab notebook in the EmmaEmb repository.

#### Library design and extensibility

The EmmaEmb library is designed as a modular framework that can be extended for further types of analysis, such as clustering approaches or topological analysis. Additionally, the repository includes a script that facilitates the extraction of embeddings from five PLMs listed in Table 3, including sequence chopping and embedding rejoining algorithms to process long sequences.

**Table 3:** Overview of the protein language models used in this study

Model name	Reference	Version	No. of parameters	Embedding length	Training
Ankh	Elnaggar et al.^48^	large	1.15 billion	1,536	only trained on amino acid sequence data
ProtT5	Elnaggar et al.^2^	prot_t5_xl_uniref. 50	3 billion	1,024	only trained on amino acid sequence data
ProstT5	Heinzinger et al.^6^	–	3 billion	1,024	ProtT5 fine-tuned for translation between sequence and structure
ESM-2	Lin et al.^3^	esm2_t36_3B_UR50D^a^	3 billion	2,560	only trained on sequence data
ESM C	ESM Team^49^	esmc-300m-2024-12	300 million	960	related to the multi-modal model ESM3 (Hayes et al.^4^), but optimized for representation learning

a For ligand-binding sites (three-class), a different ESM-2 model version was used: esm2_t33_650M_UR50D. All other tasks used esm2_t36_3B_UR50D.

### Experiments

We applied EmmaEmb to the following six molecular biology applications and datasets. A summary of each dataset, including the distributions of samples per class, is provided in Tables S1–S6. We performed experiments with seven different embedding models: five PLMs^2^^,^^3^^,^^4^^,^^6^^,^^48^ (Table 3), a representation-adapted fine-tuned embedding model (ProtTucker),^50^ and a supervised deep-learning model (AlphaFold2).^1^

#### Datasets

##### Subcellular localization

We utilize the training set of version 1.0 of the DeepLoc dataset, which consists of 11,231 protein sequences from eukaryotes annotated with subcellular localization information across ten distinct classes.^51^

##### Residue conservation

We obtain residue conservation scores from the ConSurf10k datasets provided by Marquet et al.,^14^ which classifies each residue into one of nine conservation categories ranging from 1 (highly variable) to 9 (highly conserved) based on ConSurf-DB.^52^ Due to computational constraints, we excluded proteins with lengths >1,022 residues, resulting in a dataset of 2,398,737 residue annotations.

##### Residue binding (binary)

The residue-binding dataset comprises 170,686 annotated residues from the *DevSet1014*,^16^ including binding to small molecules, metal ions, and DNA/RNA (excluding protein-protein interactions). Each residue is labeled as either binding or non-binding.

##### Ligand-binding site (three-class)

We constructed a new dataset^53^ combining three sources for multi-class residue-level annotation of ligand-binding sites, distinguishing between regular binding, cryptic binding, and non-binding residues. The training set was taken from the CryptoBench train split (cryptic)^54^^,^^55^ and sc-PDB enhanced (regular), an augmented version of sc-PDB in which additional pockets identified in alternative protein conformations were added using AHoJ-DB.^56^^,^^57^^,^^58^^,^^59^^,^^60^^,^^61^^,^^62^ A test set was compiled from the LIGYSIS benchmark (regular),^63^^,^^64^ a recently published dataset of ligand-binding human proteins, and the CryptoBench test split (cryptic). Data leakage was controlled separately for each source: the CryptoBench train/test split was used as provided, and scPDB-enhanced was filtered to a maximum of 10% pairwise sequence identity against the LIGYSIS test set using MMseqs2.^65^ To address class imbalance, all three classes were randomly downsampled to match the smallest class in both the training and test sets, yielding a balanced training set of 38,898 residues (∼12,966 per class) across 1,082 proteins and a balanced test set of 9,336 residues (∼3,112 per class) across 282 proteins. Therefore, each residue was annotated as one of three classes: regular binding, cryptic binding, or non-binding.

##### Protein structure (CATH)

We use the *test300* dataset from CATH version 4.3,^66^ as defined by Heinzinger et al.^50^ This set contains 300 proteins. For our analysis, we focus on the highest hierarchical level, class C, which groups proteins based on their secondary structure composition into five classes: mainly alpha, mainly beta, alpha beta, few secondary structures, and special.

##### Phospholipase A2 family

This dataset consists of proteins from the phospholipase A2 family, specifically the phospholipase A2 isozyme group II (PLA2G2).^67^^,^^68^ The dataset includes protein sequences annotated across seven different species and categorized into 11 different enzyme classes. Only the proteins with both species and enzyme class information were included, resulting in a final set of 446 proteins for our analysis.

#### Embeddings

##### PLM embeddings

We validated EmmaEmb analysis methods using five transformer-based PLMs: Ankh,^48^ ProtT5,^2^ ProstT5,^6^ ESM-2,^3^ and ESM C^49^ (Table 3). For residue-level prediction tasks, we use the embedding corresponding to the respective residue position. For all models, embeddings were extracted from the final hidden layer of each respective model. As some of these models have restrictions on the maximum sequence length, protein sequences longer than 1,022 amino acids were excluded from residue-level analyses, which affected 34 genes in the residue conservation task. For protein-level prediction tasks, we compute the mean of residue-level embeddings to obtain a single vector per protein. For sequences exceeding the maximum input length, protein sequences are divided into smaller chunks, and each chunk is processed with a minimum overlap of 300 amino acids to maintain sequence continuity. As proposed by Brandes et al.,^69^ to ensure that the entire sequence is effectively represented, chunks were taken with overlapping windows from both sides, and, if necessary, an additional 1,022-amino-acid window was included in the middle to ensure full coverage of the input sequence. The embeddings for each chunk are then aggregated using the mean of all chunk embeddings, resulting in a single unified representation for the full-length protein. For convenience, this embedding pipeline, including chunking and aggregation, is implemented in the EmmaEmb library.

##### ProtTucker embeddings

We obtain ProtTucker embeddings using the code and pretrained models provided by the authors.^50^ We follow their default settings without modifications to ensure consistency with the original work. For protein-level analyses, we compute the mean of the residue-level embeddings to obtain a single vector representation per protein.

##### AlphaFold embeddings

AlphaFold2 embeddings were generated using ColabFold^70^ (v.1.5.2) with default parameters, including MMseqs2^65^ for generation of multiple sequence alignments. Specifically, we extract the single representation, a residue-level embedding produced during the structure prediction process, and perform mean pooling across the residue embeddings to retrieve protein-level embeddings.

Geometric diagnostics were run for all embedding spaces and datasets used in this study. Where anisotropy was detected, mean centering was applied to the embedding space as a correction before all subsequent analysis steps including hubness computation, distance calculation, and neighborhood scoring. Diagnostic outputs and whether mean centering was applied are reported for each embedding space and dataset in Table S9.

#### Transfer model for ligand-binding site (three-class) prediction

For the ligand-binding-site task, we trained a shallow three-layer multi-layer perceptron (MLP) with 1,024 neurons for each layer.^53^^,^^71^ The model was trained for 10 epochs. A dropout of 0.3 was utilized, together with AdamW optimizer and learning rate of 5 × 10^−6^, batch size equal to 2,048, and focal loss with gamma of 7. A single model was trained for each embedding space. The hyperparameters were determined using a random 80/20 split optimizing for area under the precision-recall curve (AUPRC). An overview of model performance across a range of evaluation metrics (accuracy, area under the curve [AUC], Matthews correlation coefficient, F1 score, and AUPRC) is provided in Table S8.

#### Statistical tests

All statistical comparisons between KNN feature alignment score distributions of models were performed using one-sided Wilcoxon signed-rank tests, testing whether model 1 had greater values than model 2. Rank-biserial correlations are reported as effect-size measures. *p* values and rank-biserial correlations were computed with the Python package Pingouin.^72^

## Results

In this section, we report the results and insights of embedding analysis experiments conducted using EmmaEmb on a diverse set of molecular biology applications and seven different embedding models.

### Embedding-space analysis using EmmaEmb ranks PLM representations by their relevance for downstream tasks

We investigated whether direct analysis of the embedding-space structure using EmmaEmb can serve as a proxy for evaluating the task-relevant information captured by different PLMs. Specifically, we assessed whether the local organization of embeddings of samples, measured through the distribution of task-specific labels within their neighborhoods, can be used to rank embedding spaces in a manner consistent with their performance on the downstream task. In this study, transfer learning refers to an approach in which representations learned by a large pretrained model are used as input features for a smaller task-specific model.

We evaluated four PLMs—Ankh,^48^ ProtT5,^2^ ProstT5,^6^ and ESM-2^3^—across four benchmark classification tasks for proteins: subcellular localization (i.e., protein location within a cell),^51^ residue conservation (evolutionary persistence of amino acids),^14^^,^^52^ residue binding (binary annotation of amino acids as binding or non-binding),^16^ and ligand-binding site (three-class annotation of residues as non-binding, binding, or cryptic; where cryptic binding sites are absent or occluded in the ligand-free protein conformation but become accessible upon conformational change, making them of particular interest as drug targets). For the first three tasks, we benchmark the ranking of embedding spaces using EmmaEmb against published transfer learning performance from Heinzinger et al.,^6^ including a light-attention model for subcellular localization and two-layer convolutional neural networks for residue conservation and binary residue binding, providing an independent comparison that does not rely on classifiers trained in this study. For the ligand-binding site (three-class) task, we trained a dedicated MLP classifier on a newly constructed dataset (see methods). This enables direct comparison of EmmaEmb rankings against transfer learning performance generated in this study. For each task, we computed the mean KNN feature alignment scores (Align, Equation 2) using EmmaEmb across multiple neighborhood sizes (*k* values) and distance metrics (cosine, Manhattan, and Euclidean). To handle the large-scale residue conservation and ligand-binding site datasets (∼2.4 million embeddings), we used the ANNOY algorithm^44^ with 100 trees to efficiently approximate KNNs, demonstrating the scalability of EmmaEmb to millions of samples. Table S7 summarizes the transfer model training and test datasets, model architectures, and performance metrics and measures applied to limit homology between training and test sets for the four tasks. Importantly, EmmaEmb KNN feature alignment scores were computed on training set embeddings and compared against transfer learning performance evaluated on held-out test sets, ensuring that the correspondence between the two measures reflects genuine generalization of embedding structure.

**Figure 3:**
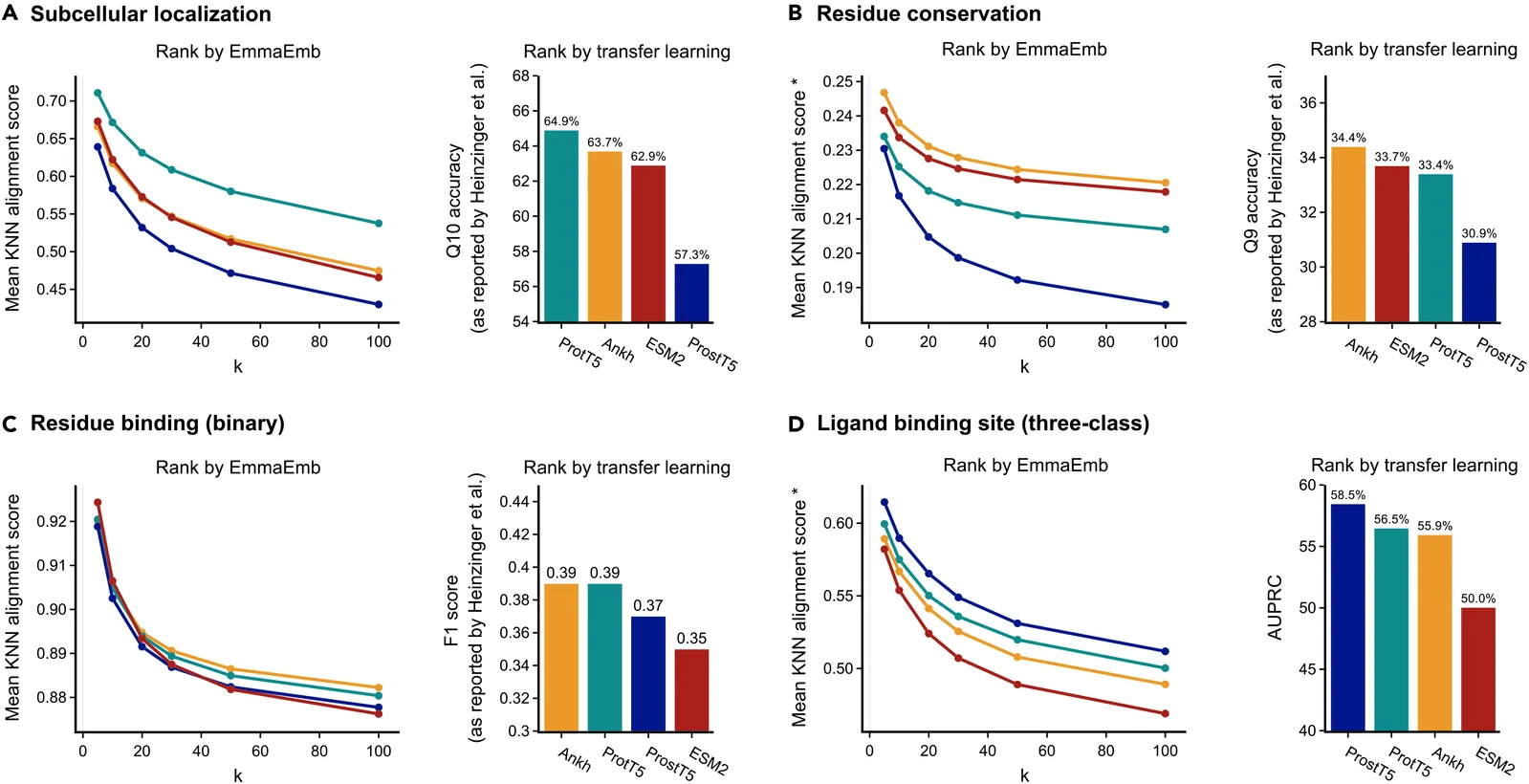
Representational relevance of protein language models assessed by embedding-space analysis vs. transfer learning Mean KNN feature alignment scores (*y* axis, EmmaEmb, cosine distance) across varying *k* values (*x* axis) are shown along downstream transfer learning performance (bar charts) for the four PLMs Ankh,^48^ ProtT5,^2^ ProstT5,^6^ and ESM-2^3^ across four tasks. Embedding-space analysis was performed on training set embeddings; transfer learning performance was evaluated on held-out test sets. (A) Protein subcellular localization (transfer learning performance: Q10, 10-state-per-protein accuracy, as reported by Heinzinger et al.^6^). (B) Residue conservation (transfer learning performance: Q9, 9-state-per-residue accuracy, as reported by Heinzinger et al.^6^; KNN approximated using the ANNOY algorithm^44^). (C) Residue binding, binary (transfer learning performance: F1 score, as reported by Heinzinger et al.^6^). (D) Ligand-binding site, three-class (transfer learning performance: AUPRC, generated in this study using an MLP classifier trained on combined CryptoBench and scPDB-enhanced training sets and evaluated on the combined LIGYSIS and CryptoBench test sets; see methods). For all four tasks, the ranking of embedding spaces by mean KNN feature alignment score is consistent with their ranking by downstream transfer learning performance, i.e., the line order at large *k* mirrors the bar-chart order. For tasks (B)–(D), this consistency is observed only after mean centering correction and with cosine distance; results prior to mean centering and across all three distance metrics (cosine, Manhattan, Euclidean) are provided in Figure S1.

For subcellular localization, mean KNN feature alignment scores consistently ranked ProtT5 highest across all *k* values (range 1–100) and ProstT5 lowest, while the relative ranking of Ankh and ESM-2 varied slightly across *k* values (Figure 3A). This ranking pattern was consistent before and after mean centering and across distance metrics (Figure S1A). Statistical testing confirmed these trends: ProtT5 scores were significantly higher than those of Ankh and ESM-2 (one-sided Wilcoxon signed-rank test at *k* = 100: rank biserial correlations 0.41–0.50; all *p* < 10^−10^), and both Ankh and ESM-2 significantly outperformed ProstT5 (rank biserial correlations 0.22–0.37; all *p* < 10^−10^; Table S10). This embedding-based ranking using EmmaEmb reflects overall reported model performance on this task, with ProtT5 achieving the highest transfer accuracy (10-state per-protein accuracy = 64.9%), ahead of Ankh (63.7%), ESM-2 (62.9%), and ProstT5 (57.3%).^6^

For the residue conservation task (Figure 3B), geometric diagnostics revealed strong hubness for ESM-2 embeddings prior to correction (RHI = 0.958), which substantially resolved after mean centering (RHI = 0.494; Table S9). The geometric correction impacted the observed embedding-space rankings: prior to mean centering, the ranking of ESM-2 relative to other models was inconsistent with downstream transfer learning performance (Figure S1B). After mean centering, EmmaEmb ranked Ankh, ESM-2, ProtT5, and ProstT5 (high to low) across *k* values (1–100), consistent with the downstream transfer learning performance (9-state per-residue accuracy = 34.4%), followed by ESM-2 (33.7%), ProtT5 (33.4%), and ProstT5 (30.9%).^6^ This demonstrates that correcting for geometric biases before analysis is not merely a technical formality but can directly affect conclusions about model prioritization. Statistical testing confirmed this ordering: Ankh scored significantly higher than ESM-2 (one-sided Wilcoxon signed-rank test at *k* = 100: rank biserial correlation 0.05, *p* < 10^−10^), ESM-2 was higher than ProtT5 (rank biserial correlation 0.23, *p* < 10^−10^), and ProtT5 outperformed ProstT5 (rank biserial correlation 0.20, *p* < 10^−10^) (Table S11).

For binary residue binding (Figure 3C), geometric diagnostics again revealed strong hubness for ESM-2 embeddings prior to correction (RHI = 0.560), which was substantially reduced after mean centering (RHI = 0.268; Table S9). Prior to mean centering, embedding-space rankings fluctuated substantially across distance metrics (Figure S1C). Following correction, EmmaEmb consistently ranked Ankh above ProtT5 and ProtT5 above ProstT5 across *k* values. ESM-2 ranked highest at small *k* values but lowest at larger *k* values (*k* = 50–100), preventing a reliable ranking for this model. Statistical testing confirmed the ordering among the three stable models: Ankh scores were significantly higher than those of ProtT5, and ProtT5 scores were significantly higher than those of ProstT5 (one-sided Wilcoxon signed-rank test at *k* = 100: rank biserial correlations 0.06–0.09; all *p* < 10^−10^; Table S12). The ranking of Ankh, ProtT5, and ProstT5 is broadly consistent with their downstream transfer learning performance (F1 score: Ankh = 0.39, ProtT5 = 0.39, ProstT5 = 0.37, and ESM-2 = 0.35).

A key challenge for this dataset is its severe class imbalance (8.2% binding vs. 91.8% non-binding), which may affect neighborhood-based metrics, as the proportion of same-class neighbors a binding residue can have is inherently constrained by the rarity of the class. To assess the robustness of embedding-space rankings under different balancing conditions, we performed progressive class rebalancing by progressively downsampling the majority (Figure S2). Rankings for Ankh, ProtT5, and ProstT5 remained consistent across subsampling ratios, while ESM-2 showed unstable ranking relative to the other models, indicating that its ranking is sensitive to class distribution and should be interpreted with caution. We additionally performed progressive feature perturbation ablations, where an increasing fraction of annotations was randomly shuffled while embeddings were held fixed (Figure S3). Despite the strong class imbalance, all four models’ mean KNN feature alignment scores declined progressively with increasing permutations, converging toward the expected random baseline.

For ligand-binding sites (three-class) (Figure 3D), geometric diagnostics revealed anisotropy resolved by mean centering, especially for ESM2 embeddings prior to correction (average cosine similarity 0.356 before and 0.003 after mean centering; Table S9). As observed for residue conservation, embedding-space rankings prior to mean centering were inconsistent with downstream transfer learning performance across distance metrics (Figure S1D). Following correction, EmmaEmb ranked ProstT5 highest, followed by ProtT5, Ankh, and ESM-2 across *k* values, consistent with their downstream transfer learning performance (AUPRC: ProstT5 = 58.5%, ProtT5 = 56.5%, Ankh = 55.9%, and ESM-2 = 50.0%). Statistical testing confirmed the EmmaEmb ordering (one-sided Wilcoxon signed-rank test at *k* = 100: rank biserial correlations 0.09–0.17; all *p* < 10^−10^; Table S13).

Together, these results demonstrate that EmmaEmb can quantify how readily task-relevant information is encoded and accessible in an embedding space, offering a fast, training-free proxy for transfer learning performance and model prioritization, as shown here against both published transfer learning benchmarks and a classifier trained in this study. Correcting for geometric biases and evaluating rankings across *k* values, class balance, and feature noise conditions allows users to assess not just the ranking itself but the confidence with which it can be reported.

### EmmaEmb analysis captures class-wise transfer learning performance and provides insights into misclassification patterns

Building on the model-level comparisons described above, we next examined whether embedding-space neighborhoods could capture class-specific transfer learning performance and misclassification tendencies. Per-class transfer learning performance was available for subcellular localization and ProtT5 from Stärk et al.^15^ for residue conservation and ProtT5 from Marquet et al.,^14^ as well as for the ligand-binding site (three-class) task from the MLP classifier trained in this study, enabling class-level analysis across all multi-class tasks examined and across both literature-reported and study-generated transfer learning benchmarks.

**Figure 4:**
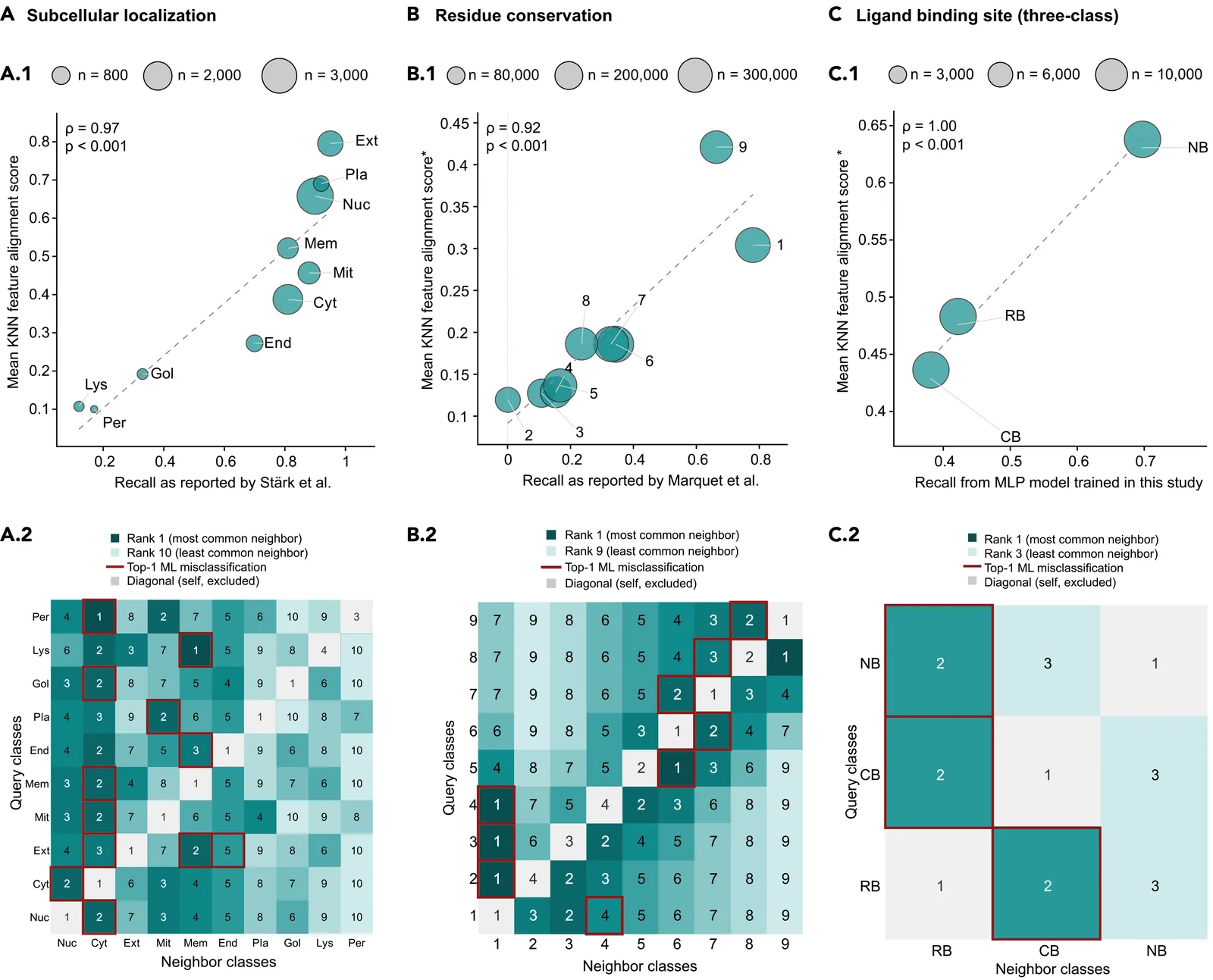
Class-level embedding-space analysis vs. transfer learning analysis across three tasks Embedding-space analysis based on *k* = 100, cosine distance, performed on training set embeddings after mean centering. All panels (A.1, B.1, C.1) on the top row show scatterplots of mean KNN feature alignment scores (*y* axis, EmmaEmb) vs. per-class transfer learning recall (*x* axis), with dot size reflecting class sample count; asterisk on the *y*-axis label indicates that KNN neighborhoods were approximated using the ANNOY algorithm. All panels on the bottom row (A.2, B.2, C.2) show KNN class ranking matrices where, for each query class (*y* axis), numbers and colors indicate the ranked frequency of each neighbor class in the embedding space from most to least common. Red boxes indicate the top-1 misclassification as reported by the transfer learning model. Diagonal cells (self, excluded) are shown in gray. Fractional neighbor class distributions underlying each ranking matrix are provided in Figure S4. (A) Subcellular localization. ProtT5 embedding space. Transfer learning performance as reported by Stärk et al.^15^ An overall high correlation (Spearman's *ρ* = 0.97, *p* < 0.001) is observed, with some smaller classes (e.g., Pla, plastid) both well predicted and well represented in the embedding space. For nine out of ten classes, the most frequently misclassified class is also the most common neighboring class in the embedding space; for the extracellular (Ext) class, three classes share an equal misclassification pattern. Cyt, cytoplasm; End, endoplasmic reticulum; Ext, extracellular; Gol, Golgi apparatus; Lys, lysosome/vacuole; Mem, cell membrane; Mit, mitochondrion; Nuc, nucleus; Per, peroxisome; Pla, plastid. (B) Residue conservation. ProtT5 embedding space. Transfer learning performance as reported by Marquet et al.^14^ The correlation between per-class EmmaEmb alignment scores and per-class transfer learning recall is high (*ρ* = 0.92, *p* < 0.001). For six of nine classes, the most frequently misclassified class is also the most common neighboring class in the embedding space; for two further classes, the most frequently misclassified class is the second most common neighboring class. Conservation categories 1–9 range from highly variable to highly conserved. (C) Ligand-binding site (three-class). ProstT5 embedding space, selected as the best-performing embedding space for this task. Transfer learning performance from the MLP classifier trained in this study. The correlation between per-class EmmaEmb alignment scores and per-class transfer learning recall is perfect (*ρ* = 1.00, *p* < 0.001). For all three classes, the most frequently misclassified class is also the most common neighboring class in the embedding space. The lower per-class recall for regular and cryptic binding residues (C.1) reflects the classifier’s tendency to mix these two structurally similar classes, as shown in the confusion matrix (C.2). NB, non-binding; RB, regular binding; CB, cryptic binding.

For all three tasks, we computed mean KNN feature alignment scores (Align, Equation 2, *k* = 100, cosine distance) for each class and compared them with per-class transfer learning recall. A strong Spearman correlation between per-class EmmaEmb alignment scores and per-class transfer learning recall was observed across all three tasks: *ρ* = 0.97 (*p* < 0.001) for subcellular localization, *ρ* = 0.92 (*p* < 0.001) for residue conservation, and *ρ* = 1.00 (*p* < 0.001) for ligand-binding site (three-class) (Figures 4A.1–4C.1). Notably, this also held for smaller class sizes; for example, in the subcellular localization task, embeddings for proteins in the plastid, despite their relatively low representation (605 proteins, approximately 5% of the overall dataset), showed the second highest prediction recall in transfer learning (0.92, Figure 4A.1), and also second highest mean KNN feature alignment scores (0.69, Figure 4A.1) in the embedding space.

To better understand the relationship between different feature classes in the embedding space, we calculated the KNN class ranking matrix (*R*, derived from *M*, Equation 3), which analyzes the proximity of different feature classes with other feature classes in the embedding space. When comparing these distributions with the predicted class distributions from the transfer learning models, we found a close structural similarity across all three tasks (Figures 4A.2–4C.2; full fractional distributions in Figure S4). For subcellular localization, the most frequently misclassified class is also the most common neighboring class in the embedding space for nine of ten classes. For residue conservation, this holds for six of nine classes, with the most frequently misclassified class appearing as the second most common neighbor for two further classes. For ligand-binding site (three-class), all classes show this pattern. For classes that are not yet well differentiated in the embedding space, such as “cytoplasm,” our approach allows a deeper investigation into which neighboring feature classes they tend to mix with. For example, proteins labeled as “cytoplasm” were frequently embedded near proteins from the “nucleus” and “mitochondrion” classes, both of which are common sources of misclassification by the transfer learning model. The insights expose the structural roots of model errors and can guide more targeted approaches to model refinement and domain-specific fine-tuning.

### EmmaEmb supports analysis for diverse embedding models and assessment of model learning

**Figure 5:**
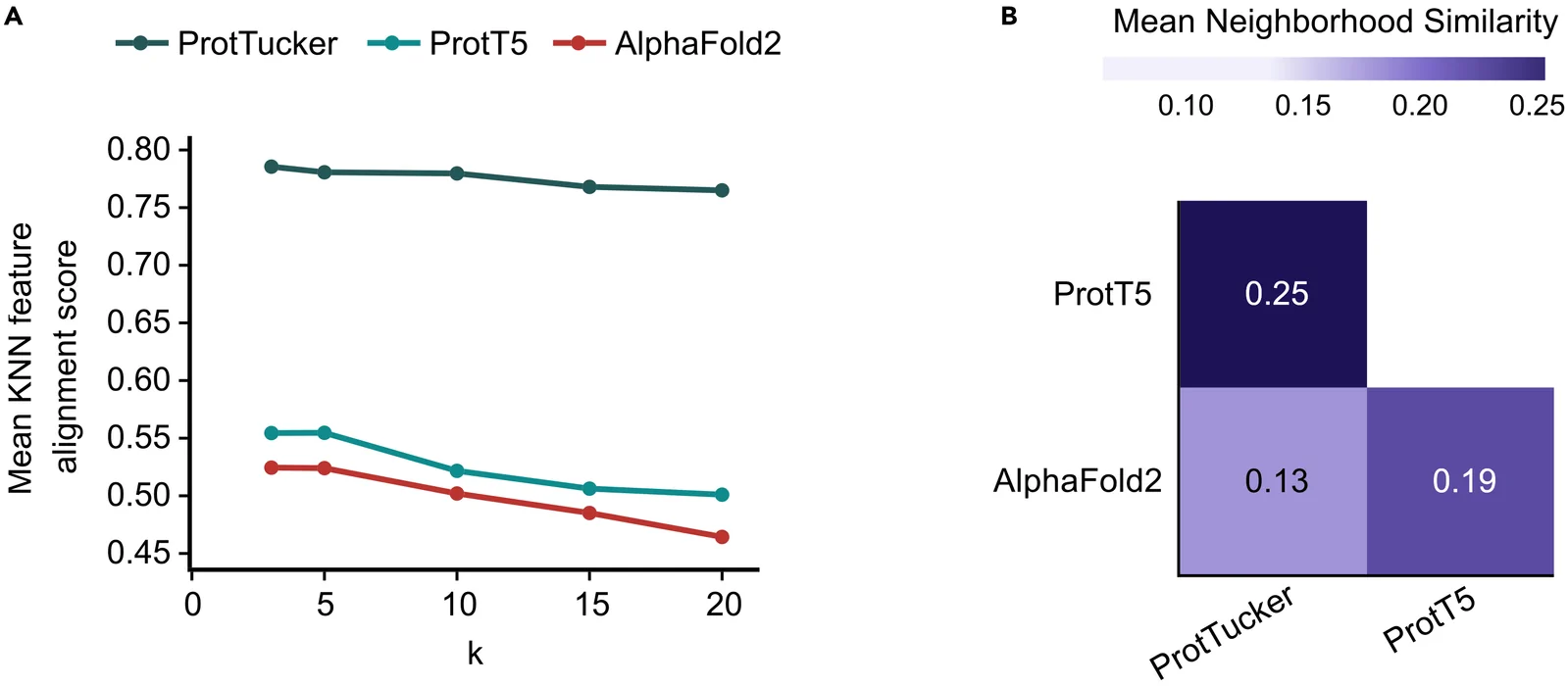
Comparison of ProtT5, ProtTucker (ProtT5 adapted to CATH hierarchy), and AlphaFold2 embeddings for protein structural representation (A) Mean KNN feature alignment scores (*y* axis) for CATH class feature across varying neighborhood sizes (*k*, *x* axis), shown for cosine distance after mean centering. Each line represents one embedding model. ProtTucker, which is explicitly fine-tuned for CATH structural classification using contrastive learning, consistently shows higher mean KNN feature alignment scores than ProtT5 and AlphaFold2 across all *k* values. AlphaFold2 embeddings show alignment scores comparable to ProtT5 and consistently below ProtTucker, despite being trained on structural data. Results for Manhattan and Euclidean distance are provided in Figure S5. (B) Mean cross-space neighborhood similarity (shown for *k* = 20, cosine distance) between pairs of embedding models, displayed as a 2 × 2 matrix where each cell shows the mean similarity between the corresponding model pair. The ProtTucker-ProtT5 pair shows highest mean neighborhood similarity (0.25), indicating that contrastive fine-tuning reshaped the ProtT5 embedding space in a targeted manner while preserving core representational geometry. Both ProtTucker-AlphaFold2 (0.13) and ProtT5-AlphaFold2 (0.19) show lower similarity. Full results across *k* values and distance metrics are provided in Figure S6.

To examine how embedding-space analysis can be used to monitor representational changes during model adaptation, we compared embeddings from the original ProtT5 model with those from ProtTucker,^50^ a model that adds task-specific layers to adapt the ProtT5 embedding space for CATH structural classification^66^ using contrastive learning. We analyzed the embeddings of 300 proteins from the ProtTucker test set that were not seen during the ProtTucker training. Across a range of neighborhood sizes and distance metrics, ProtTucker embeddings consistently exhibited higher mean KNN feature alignment scores with respect to CATH class feature than ProtT5 (Figures 5A and S5A), reflecting improved capture of structural hierarchy information by ProtTucker embeddings.

We next compared ProtTucker and ProtT5 embeddings to those from AlphaFold2,^1^ a model that also captures protein structural information. Interestingly, AlphaFold2 embeddings exhibited KNN feature alignment scores comparable to those of ProtT5, while scores in both AlphaFold2 and ProtT5 remained consistently below those of ProtTucker (Figure 5A). This trend was further confirmed by statistical testing, which showed the KNN feature alignment scores to be significantly higher for ProtTucker embeddings compared to ProtT5 and AlphaFold2 embeddings (one-sided Wilcoxon signed-rank test at *k* = 20: rank biserial correlations 0.77–0.87; all *p* < 10^−10^; Table S14) and prompted further investigation into the structure of these embedding spaces. To quantify representational overlap, we computed cross-space neighborhood similarity (Sim, Equation 4) between the three embedding spaces. Across all tested *k* values and distance metrics, the mean cross-space neighborhood similarity was higher between ProtTucker and ProtT5 than between either and AlphaFold2 (Figures 5B andS5B). Again, the trends were confirmed by statistical testing showing higher cross-space neighborhood similarity for ProtTucker vs. ProtT5 compared to ProtT5 vs. AlphaFold2 (one-sided Wilcoxon signed-rank test at *k* = 20: rank biserial correlation 0.41; *p* < 10^−8^; Table S15) as well as higher scores for ProtT5 vs. AlphaFold2 compared to ProtTucker vs. AlphaFold2 (rank biserial correlation 0.71; *p* < 10^−10^ at *k* = 20; Table S15). This suggests that ProtTucker retains core structural characteristics from ProtT5 while incorporating new task-specific organization, whereas AlphaFold2 encodes structural information in a distinct and different manner.

ProtTucker, explicitly fine-tuned for CATH classification using contrastive learning, shows strong neighborhood alignment for CATH features and serves as a positive control confirming EmmaEmb’s ability to detect known embedding structure. Notably, AlphaFold2, despite being trained on structural data, showed no advantage over ProtT5 in neighborhood alignment with CATH classes, and its embedding space was more dissimilar to both ProtT5 and ProtTucker than those two were to each other. This suggests that structural information is encoded through distinct representational routes in sequence-based vs. structure-based models, a finding that would not be accessible from downstream task performance alone. More broadly, these results demonstrate that embedding-space analysis provides a quantitative, architecture-agnostic approach to evaluating how learning objectives shape representations. It can uncover latent similarities and distinctions between models trained with different objectives or architectures, with minimal computational overhead.

### Pairwise embedding-space comparison using EmmaEmb reveals and contextualizes differences in information captured by PLMs

**Figure 6:**
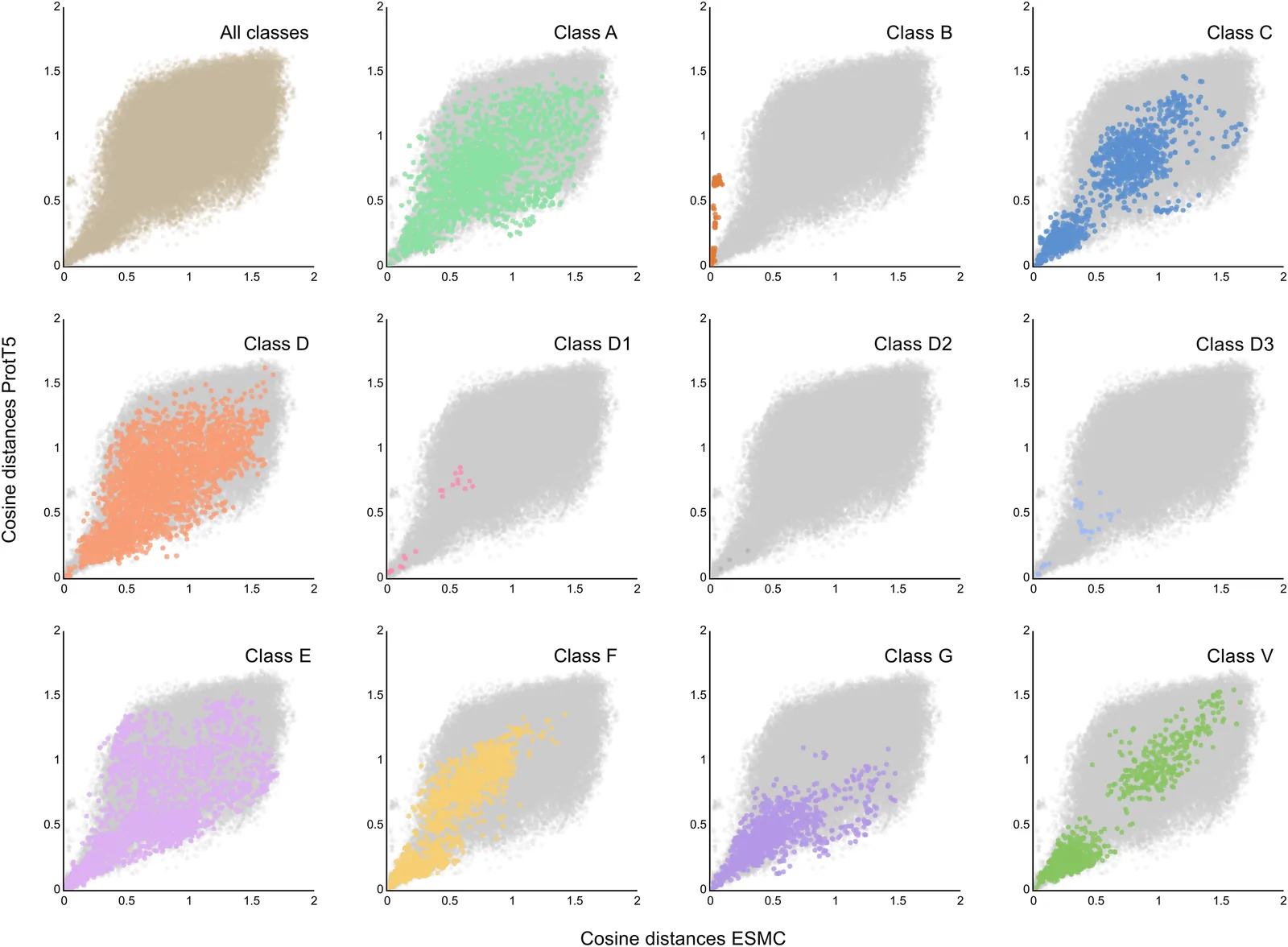
Pairwise embedding-space comparison between ESM C and ProtT5 embeddings on PLA2G2 enzyme proteins Pairwise cosine distances between PLA2G2 protein embeddings with ESM C (*x* axis) and ProtT5 (*y* axis) for all pairs (all classes, Spearman's *ρ* = 0.55, *p* < 0.001) and for pairs belonging to the same enzyme class (one panel per class, colored points; all other pairs are shown in gray). A distinct cluster of pairs with comparatively low ESM C distances and comparatively high ProtT5 distances is visible and predominantly composed of enzyme class B pairs. This suggests that ESM C places class B proteins closer together than does ProtT5.

In previous sections, we explored embedding spaces starting from specific downstream tasks and comparing model performance in the context of the information encoded in the embeddings. Here, we reversed this perspective and adopted a model-centric view, directly comparing the embedding spaces of models using biologically meaningful features to interpret the models’ differences. Toward this end, we analyzed ProtT5 and the more recently released ESM C model,^49^ which differ in training strategies, parameter counts, and embedding dimensions (Table 3). Using a set of 446 enzyme proteins from the dataset,^67^ we performed pairwise embedding-space comparisons between ProtT5 and ESM C embedding spaces to identify regions of representational divergence and provide context for these differences, leveraging associated biologically interpretable features (Figure 6). Prior to analysis, geometric diagnostics revealed anisotropy in both embedding spaces, which was corrected by applying mean centering of each embedding space (average cosine similarity reduced by mean centering from 0.920 to 0.021 for ESM C and 0.894 to 0.005 for ProtT5, Table S9). Subsequent global correlation analysis of the pairwise cosine distances between the embeddings of the models showed a Spearman’s *ρ* of 0.55 (*p* < 0.001) between all pairwise distances of the ESM C and ProtT5 embeddings, indicating moderate overall similarity in how these two models represent relationships among the protein sequences.

Prior to mean centering, to correct for anisotropy, a visually distinct divergent cluster appeared to be driven primarily by bird protein pairs, an observation that resolved after applying the anisotropy correction (Figure S7), suggesting it was a geometric artifact rather than a genuine representational difference.

After correction, global pairwise distance analysis revealed a cluster of enzyme class B protein pairs showing comparatively low cosine distances in ESM C relative to ProtT5, indicating that ESM C places class B proteins closer together as a group than does ProtT5 (Figure 6). Enzyme class B (Pla2g2B) has been suggested to be an avian-specific clade of the PLA2G2 family, which derived independently from the enzyme class D2 in birds, is present as a single copy, has a unique N-terminal structure, and whose function remains largely unknown.^67^ To assess whether this divergence could be explained by systematic biases, we examined sequence length as a potential confounder. While class B proteins tend to have comparatively shorter sequences relative to other enzyme classes (Figure S9), we found no apparent correlation between pair distance divergence and sequence length across all pairs (Figure S8). Further analysis controlling for additional potential confounders such as taxonomic sampling density and training data overlap would be needed to substantiate the observed class B clustering.

One hypothesis for the class B divergence is that ESM C, which is related to the multi-modal ESM3 model family^4^ and incorporates structural information during training, may represent the structurally distinct class B proteins as more similar to each other than a sequence-only model such as ProtT5 would. Given the structural uniqueness of class B within the PLA2G2 family and the limited functional characterization of this clade, this remains a hypothesis for further investigation rather than a conclusion.

While the global pairwise distances between class B proteins differ between the two models, local neighborhood analysis showed that the immediate neighbors of class B proteins are highly similar across the two spaces (Figure S10), indicating that despite their collective repositioning, individual class B proteins retain a consistent local biological context in both models. This dissociation between global and local differences illustrates the complementary information captured by the two levels of analysis and highlights why analyzing embedding spaces at both scales simultaneously is essential for a complete picture of representational differences between models.

## Discussion

Despite growing interest in foundation models and their applications, systematic characterization and comparison of these models’ embedding spaces remain underexplored in the field of molecular biology (Table 1). Insights from biological data often emerge from patterns across functionally defined groups of genes or proteins rather than individual data points, calling for tools that support structured, group-level comparison of biological data grounded in features or metadata.^73^^,^^74^ In this study, we present EmmaEmb, a quantitative, model-agnostic computational framework for analyzing and comparing embedding spaces. By systematically incorporating biological features, EmmaEmb enables researchers to evaluate the organization of embeddings both locally and globally and to contextualize differences between models (Figure 1). Through rigorous experiments in a variety of molecular biology applications, we demonstrate that embedding-space analysis using EmmaEmb can reveal aspects of representational quality that align with transfer learning performance while requiring low computational overhead.

Our findings show that embedding-space neighborhoods can serve as a proxy for downstream predictive power, provided that geometric properties of the embedding space are verified and corrected before analysis (Figures 3 and 4). Neighborhood-based metrics assess whether task-relevant information is readily accessible in the embedding geometry, complementing transfer learning by providing a fast, training-free assessment of representational quality. A high-performing downstream classifier may exploit distributed or non-linear structure that distances between embeddings do not capture, meaning that a low alignment score does not imply absence of information but rather that it may require more complex models to extract. EmmaEmb is therefore best understood as a screening tool that identifies when embedding structure strongly supports a downstream task.

The guided diagnostic workflow implemented in EmmaEmb, covering anisotropy correction, hubness assessment, class imbalance evaluation, and feature noise robustness, provides users with structured, confidence-annotated embedding-space rankings rather than point estimates. A step-by-step walkthrough is provided in Note S1 and the EmmaEmb repository. The residue-binding (binary) task (Figure 3C) illustrates the value of the accompanying diagnostic tools: despite severe class imbalance, feature permutation analyses confirmed that all models’ embedding spaces encode binding-relevant information above chance (Figure S3), and progressive rebalancing analyses (Figure S2) identified which embedding-space rankings were stable and which were sensitive to class distribution, allowing conclusions to be drawn selectively and with appropriate robustness assessments.

Notably, for the ligand-binding site (three-class) task (Figures 3D and 4C), which distinguishes between non-binding, regularly binding, and cryptically binding residues, we trained a dedicated MLP classifier on a newly constructed dataset, enabling direct comparison of EmmaEmb rankings against transfer learning performance generated in this study and extending the benchmark to a clinically relevant formulation with direct implications for drug discovery.

Beyond embedding-space ranking, embedding analysis can also monitor how a model’s representations change during fine-tuning, an increasingly important capability as foundation models become more specialized.^75^ The high cross-space neighborhood similarity between ProtTucker and ProtT5 (Figure 5B) indicates that contrastive fine-tuning introduced targeted task-specific reorganization while retaining the core geometry of the base model. EmmaEmb’s cross-space similarity score therefore provides a geometry-aware measure of representational transformation, one that complements downstream performance by quantifying how much the underlying space has changed and what has been preserved. A sharp drop in cross-space similarity relative to the base model may signal that fine-tuning has overwritten general-purpose representations rather than augmenting them, a pattern associated with catastrophic forgetting,^76^ while a modest drop combined with improved task alignment suggests targeted, well-controlled adaptation.

The pairwise comparison between ProtT5 and ESM C further illustrates the value of analyzing embedding spaces at both global and local levels simultaneously. The dissociation between global and local differences observed for enzyme class B proteins has direct practical implications for how the two measures should be used in different biological contexts. Global pairwise distance structure is most informative for questions where broad organizational relationships matter, e.g., phylogenetic analysis, taxonomic clustering, or understanding how evolutionary distance is encoded across a protein family.^28^^,^^77^^,^^78^ Local neighborhood structure is most informative where fine-grained similarity is critical, e.g., identifying candidate drug targets by proximity to known binders or retrieving functionally related proteins for annotation transfer.^50^^,^^64^ Neither failure mode is detectable from a single summary metric, which is why EmmaEmb’s combination of global and local analysis is essential for principled model selection. As demonstrated here for sequence length, EmmaEmb’s feature-aware design makes it straightforward to investigate confounding effects. The enzyme class B finding should be considered a hypothesis, and future work could leverage this approach to systematically explore further potential confounders.

EmmaEmb is applicable beyond protein embeddings to a wider range of biological data modalities, gene expression, multi-omics, and cell imaging, provided that relevant metadata are available and the embedding space meets the geometric constraints.^79^^,^^80^^,^^81^ In the context of existing embedding analysis tools (Table 1), EmmaEmb is unique in combining feature-aware analysis, local and global comparisons, geometric diagnostics, and flexible neighborhood-based metrics in a single open-source framework.

We acknowledge that the metadata can be noisy, incomplete, or inconsistent, which can influence the scores computed by EmmaEmb. Further, neighborhood-based metrics are inherently sensitive to class imbalance, a mathematical consequence of minority-class neighborhood availability. To address both challenges directly, EmmaEmb implements progressive class rebalancing and feature perturbation analyses that allow users to quantify the influence of class distribution and metadata quality on embedding-space rankings and to report conclusions with explicit robustness assessments. We note that the benchmark datasets used in this study vary in the stringency and metrics applied to define train and test splits, ranging from published splits inherited from the original studies to sequence identity filtering applied in the newly constructed ligand-binding site dataset. Additional splitting strategies based on protein family, fold type, or binding-pocket similarity would provide further rigor.

The analyses presented here are based on embeddings extracted from a single, fixed representation layer per model. Applying EmmaEmb across layers within a single model is a natural direction for future work. Mean centering was applied in most embedding spaces analyzed in this study, underscoring that anisotropy correction is an important prerequisite for meaningful distance-based analysis; further investigation into anisotropy correction strategies is an interesting direction for future work. Additionally, EmmaEmb is well suited to investigate how neighborhood structure changes with commonly applied preprocessing steps such as dimensionality reduction or feature scaling.

More broadly, EmmaEmb contributes to the growing need for interpretability and trustworthiness in deep learning for molecular biology. By elucidating what biological information is captured, where misclassifications originate, and how representations change with fine-tuning, it supports principled decisions about embedding-space ranking, prioritization, and deployment. We demonstrated this utility through critical applications such as drug discovery, where opaque model behavior can undermine confidence in predictions. Future directions include applying EmmaEmb to other high-stakes applications such as clinical variant interpretation. Extensions could integrate additional interpretability tools such as sparse autoencoders^19^ or hierarchical visualization,^28^ and EmmaEmb’s modular design supports community-driven additions.

In summary, EmmaEmb provides a unified and extensible framework for quantitative comparison of embedding spaces, enabling principled model evaluation, misclassification analysis, and representational monitoring across fine-tuning. As the landscape of foundation models in molecular biology continues to expand, such tools will be indispensable for bridging the gap between raw predictive performance and biologically meaningful understanding.

## Resource availability

### Lead contact

Requests for further information and resources should be directed to and will be fulfilled by the lead contact, Sumaiya Iqbal (sumaiya@broadinstitute.org).

### Materials availability

All datasets used in this study are publicly available.

### Data and code availability

All original code regarding the EmmaEmb library, including scripts for data preprocessing and figure reproduction, has been deposited at https://github.com/broadinstitute/EmmaEmb and archived on Zenodo: https://doi.org/10.5281/zenodo.21246289.^41^ Code for the ligand-binding site classification models and associated figures is available at https://github.com/skrhakv/emb-space-analysis and archived on Zenodo: https://doi.org/10.5281/zenodo.21246342.^71^ The newly curated ligand-binding site dataset including train, evaluation, and test splits, sequence IDs, accession lists, and random seeds has been deposited on Zenodo: https://doi.org/10.5281/zenodo.21246325.^53^

## Acknowledgments

The project was supported by the Designing for Sustainability research program, a joint initiative of the Morningside Academy for Design at 10.13039/100006919MIT and the Hasso Plattner Institute, funded by the 10.13039/501100020408Hasso Plattner Foundation. This work was also supported by the Deutsche Forschungsgemeinschaft (DFG, 10.13039/501100001659German Research Foundation; project number 459422098). We express our gratitude to Eugenia Alleva, Jannis Baum, Katharina Baum, Jakub Bartoszewicz, Hilary Finucane, Theresa Hradilak, Marta Lemanczyk, and Melania Nowicka for their valuable discussions and insightful input regarding this project.

## Author contributions

Conceptualization, P.F.R., P.Y.S., J.F.S., and S.I.; data curation, P.F.R., V.S., and J.F.S.; formal analysis, P.F.R. and V.S.; funding acquisition, H.O.H. and S.I.; investigation, P.F.R. and V.S.; methodology, P.F.R., P.Y.S., B.Y.R., and S.I.; project administration, S.I.; resources, C.W.C., B.Y.R., H.O.H., and S.I.; software, P.F.R. and J.F.S.; supervision, H.O.H. and S.I.; validation, P.F.R. and V.S.; visualization, P.F.R. and V.S.; writing – original draft, P.F.R. and P.Y.S.; writing – review & editing, P.F.R., V.S., P.Y.S., J.F.S., C.W.C., B.Y.R., H.O.H., and S.I.

## Declaration of interests

No competing interests are declared.

## Declaration of generative AI and AI-assisted technologies in the writing process

During the preparation of this work, the authors used ChatGPT (GPT-4 and GPT-5 by OpenAI) and Claude Sonnet 4.6 to revise portions of the text for clarity. After using this tool or service, the authors reviewed and edited the content as needed and take full responsibility for the content of the publication.

## Supplemental information

Document S1. Figures S1–S10, Tables S1–S15, and Note S1

Document S2. Article plus supplemental information
