# Supplementary material for: EmmaEmb: A quantitative framework for analyzing embedding spaces in molecular biology
Source: Patterns (N Y). 2026 Aug 12;7(9):101635. doi: 10.1016/j.patter.2026.101635 (PMC13576657; doi:10.1016/j.patter.2026.101635)
Supplement: Supplementary file 1 [file mmc1.pdf]

Patterns, Volume *7*

Supplemental information

EmmaEmb: A quantitative framework for analyzing embedding spaces in molecular biology

Pia Francesca Rissom, Vít Škrhák, Paulo Yanez Sarmiento, Jordan F. Safer, Connor W. Coley, Bernhard Y. Renard, Henrike O. Heyne, and Sumaiya Iqbal

Table of Contents

Supplemental Figures....3

Figure S1: Mean KNN feature alignment scores across distance metrics and k-values without mean centering....3

Figure S2: Class rebalancing analysis for residue binding (binary)....5

Figure S3: Feature perturbation robustness analysis for residue binding (binary)....6

Figure S4: Fractional class distributions from transfer learning and embedding space neighborhood analysis across three tasks....7

Figure S5: Mean KNN feature alignment scores for the CATH class feature across Manhattan and Euclidean distance metrics....9

Figure S6: Mean cross-space neighborhood similarity matrices between ProtTucker, ProtT5, and AlphaFold2 across k values and distance metrics....10

Figure S7: Pairwise cosine distances between PLA2G2 protein embeddings in ESM C and ProtT5, colored by species, before and after mean centering....11

Figure S8: Pairwise cosine distances after mean centering between PLA2G2 protein embeddings in ESM C and ProtT5, colored by binned sequence length....12

Figure S9: Sequence length distribution per PLA2G2 enzyme class....12

Figure S10: Cross-space neighborhood similarity between ESM C and ProtT5 embeddings of PLA2G2 proteins, stratified by species, sequence length, and enzyme class....13

Supplemental Tables....14

Table S1: Protein counts per subcellular localization class in the subcellular localization dataset....14

Table S2: Residue counts per conservation class in the residue conservation dataset....14

Table S3: Residue counts per residue binding class in the residue binding (binary) dataset....14

Table S4: Protein counts per CATH class in the protein structure (CATH) dataset....15

Table S5: Protein counts per class in the Phospholipase A2 family (PLA2G2) dataset....16

Table S6: Residue counts per ligand binding class in the ligand binding site (three-class) dataset....17

Table S7: Overview of protein language model benchmark tasks, datasets, and reference....18

Table S8: Performance of transfer learning model for ligand binding site (three-class)....19

Table S9: Embedding space geometry diagnostics for all model-dataset combinations....20

Table S10: One-sided Wilcoxon signed-rank tests for KNN feature alignment scores at k = 100 for the subcellular localization dataset after mean centering of the embeddings....21

Table S11: One-sided Wilcoxon signed-rank tests for KNN feature alignment scores at k = 100 for the residue conservation dataset after mean centering of the embeddings....21

Table S12: One-sided Wilcoxon signed-rank tests for KNN feature alignment scores at k = 100 for the residue binding (binary) dataset after mean centering of the embeddings....21

Table S13: One-sided Wilcoxon signed-rank tests for KNN feature alignment scores at k = 100 for the ligand binding site (three-class) dataset after mean centering of the embeddings....21

Table S14: One-sided Wilcoxon signed-rank tests for KNN feature alignment scores at k = 20 for the protein structure (CATH) dataset after mean centering of the embeddings....22

Table S15: One-sided Wilcoxon signed-rank tests for neighborhood similarity scores at k = 20 for the protein structure (CATH) dataset after mean centering of the embeddings....22

Supplemental Notes....23

S1: EmmaEmb guided diagnostic workflow for embedding space ranking....23

References 27

Supplemental Figures

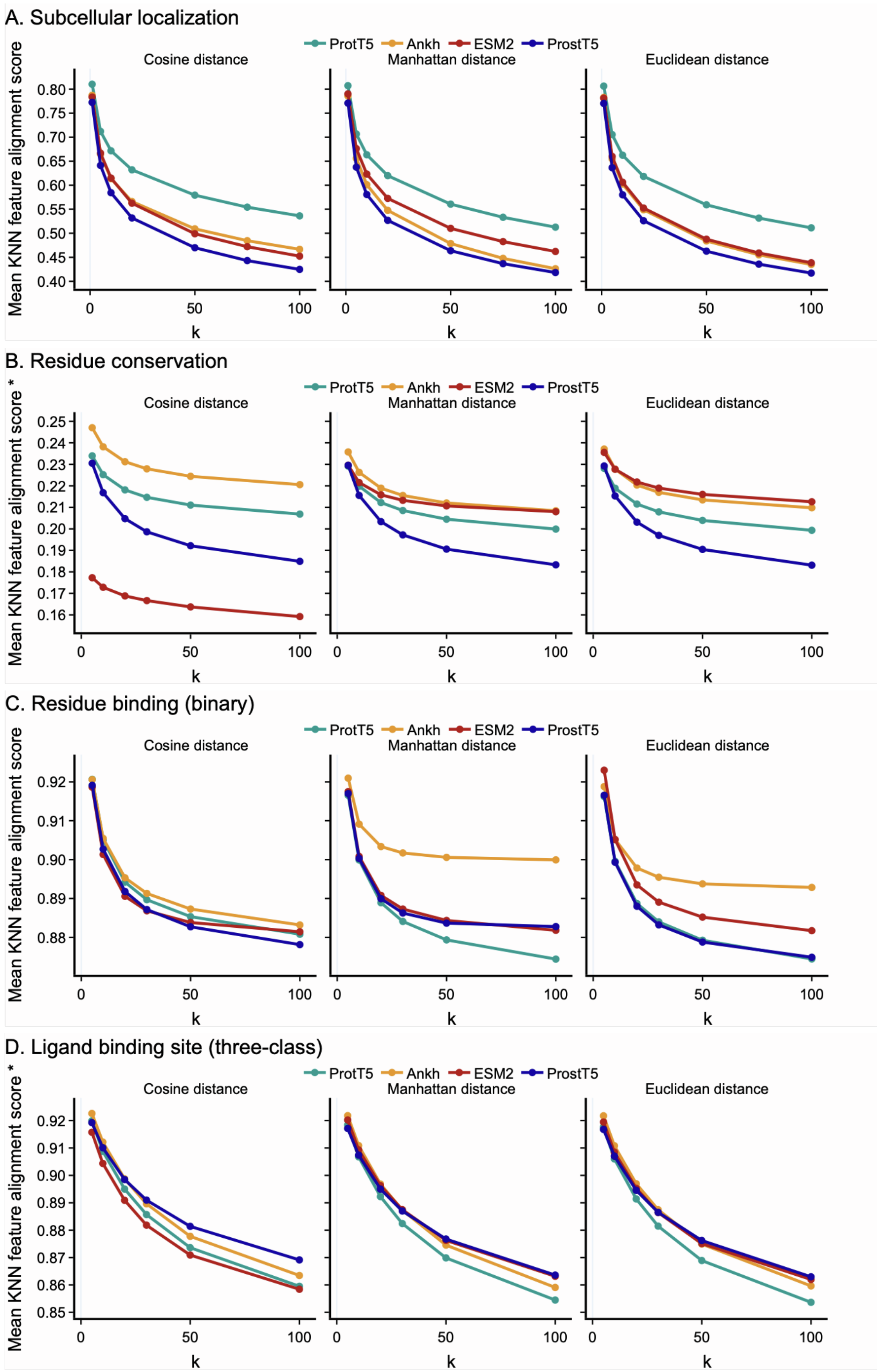

Figure S1: Mean KNN feature alignment scores across distance metrics and k-values without mean centering.

Mean KNN feature alignment scores (y-axis, EmmaEmb) across varying k-values (x-axis) are shown for three distance metrics (columns: cosine, Euclidean, Manhattan) and four PLM models (colors: ProtT5, Ankh, ESM2, ProstT5), prior to mean centering correction. An asterisk on the y-axis label indicates that KNN neighborhoods were approximated using the ANNOY algorithm. Results are shown for the datasets

(A) subcellular localization, (B) residue conservation, (C) residue binding (binary), and (D) ligand binding site (three-class). Comparison with Figure 3 (cosine distance, after mean centering) illustrates the effect of geometric correction on embedding space rankings. Notably for residue conservation and residue binding (binary), where embedding space rankings shift to align with transfer learning performance only after mean centering is applied.

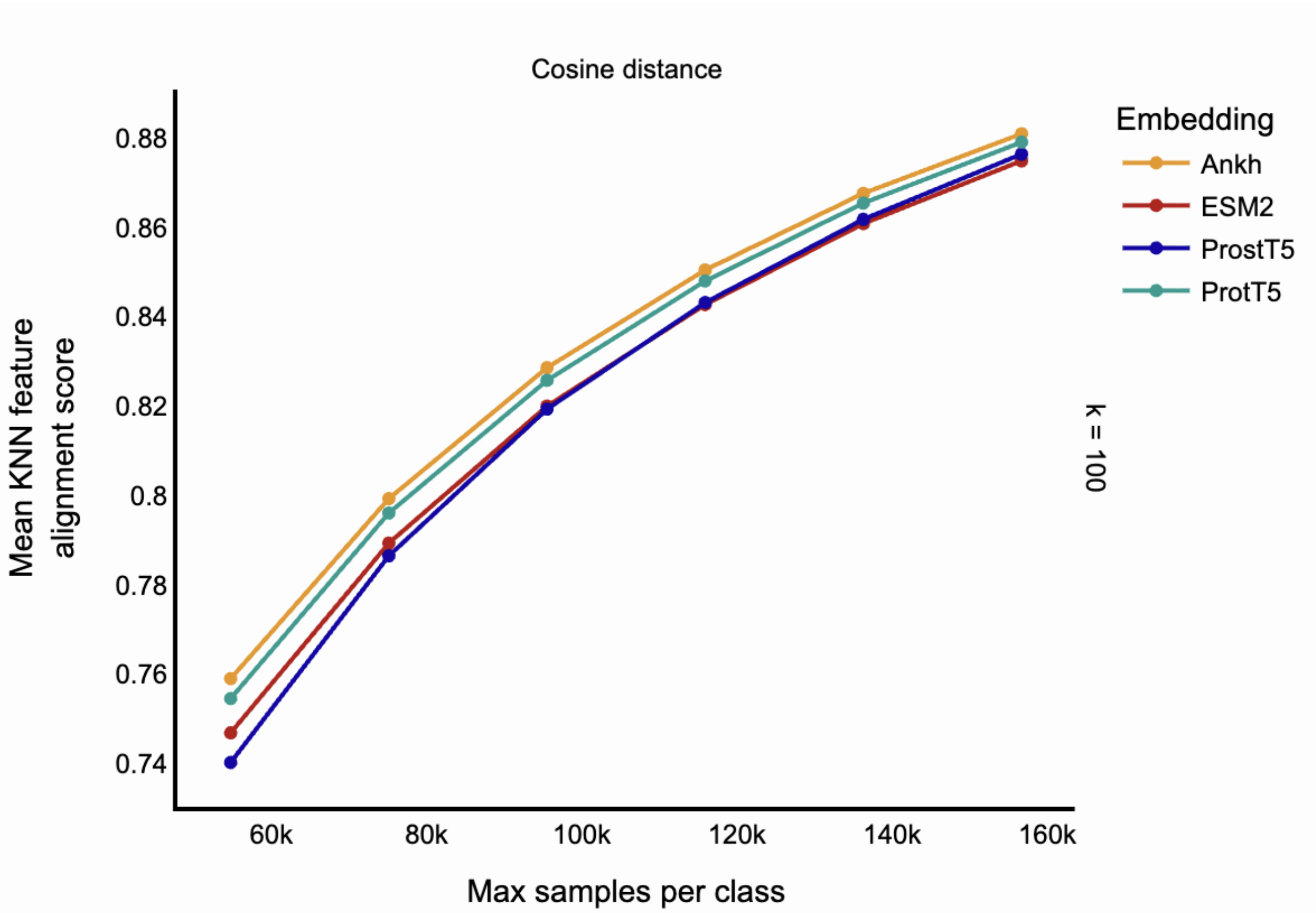

Figure S2: Class rebalancing analysis for residue binding (binary)
Mean KNN feature alignment scores (y-axis, EmmaEmb, cosine distance, k = 100, after mean centering) for four PLMs (Ankh, ESM2, ProstT5, ProtT5) as the maximum number of samples per class is progressively decreasing toward class balance by down sampling the majority class (x-axis). As the majority class is reduced, alignment scores decrease across all models, reflecting the reduced availability of same-class neighbors for the dominant non-binding class.

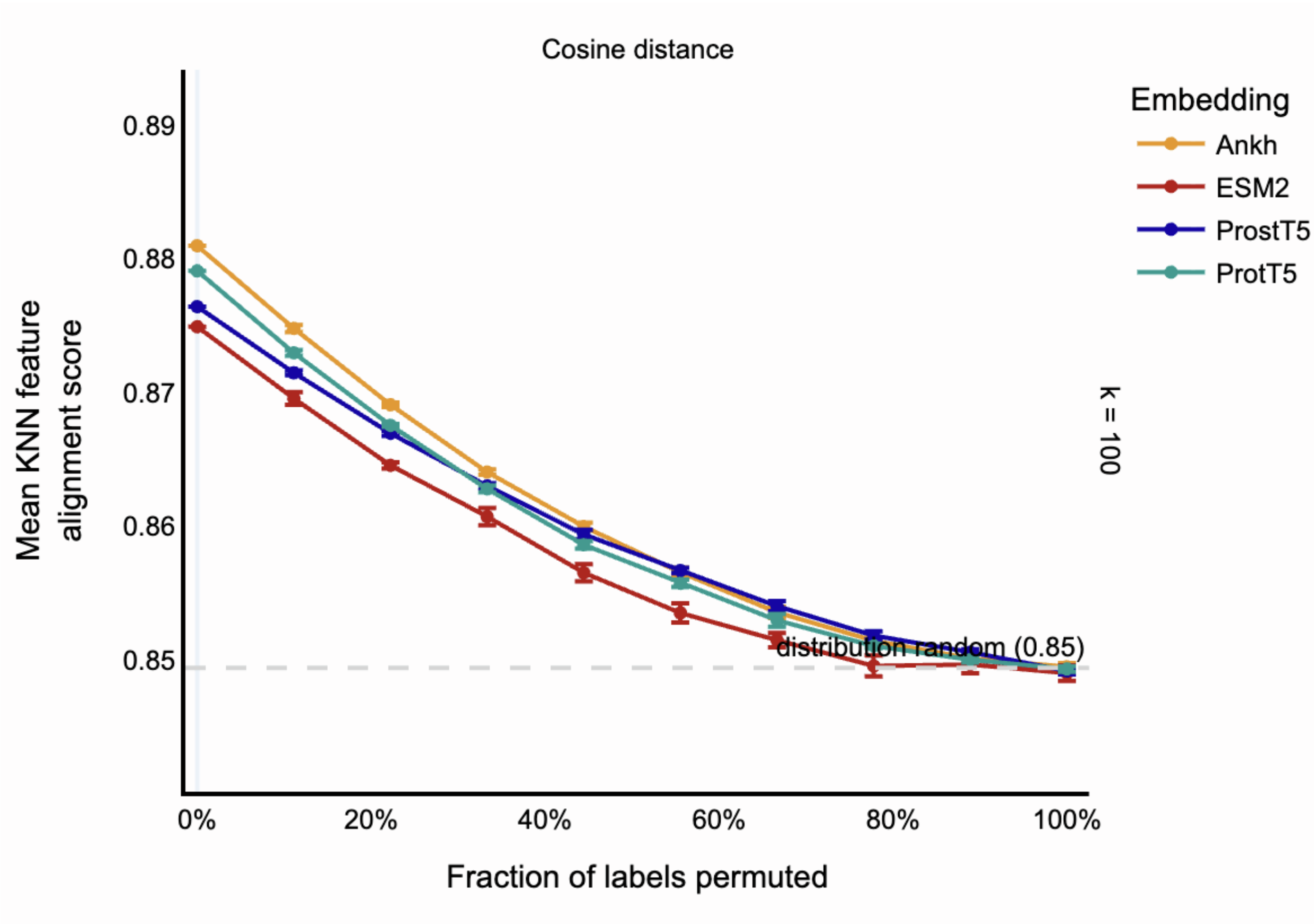

Figure S3: Feature perturbation robustness analysis for residue binding (binary)
Mean KNN feature alignment scores (y-axis; cosine distance and k = 100; after mean centering) for four PLMs (Ankh, ESM2, ProstT5, ProtT5) as an increasing fraction of features is randomly permuted (x-axis). The dashed horizontal line indicates the expected random baseline score (0.85), reflecting the alignment score that would be obtained under fully randomized feature given the class distribution. Error bars indicate variability across 5 repeated permutations. All models' scores decay toward the random baseline as permutation increases, confirming that the KNN feature alignment score captures the alignment of the embedding space with the feature rather than reflecting a property of the class distribution alone.

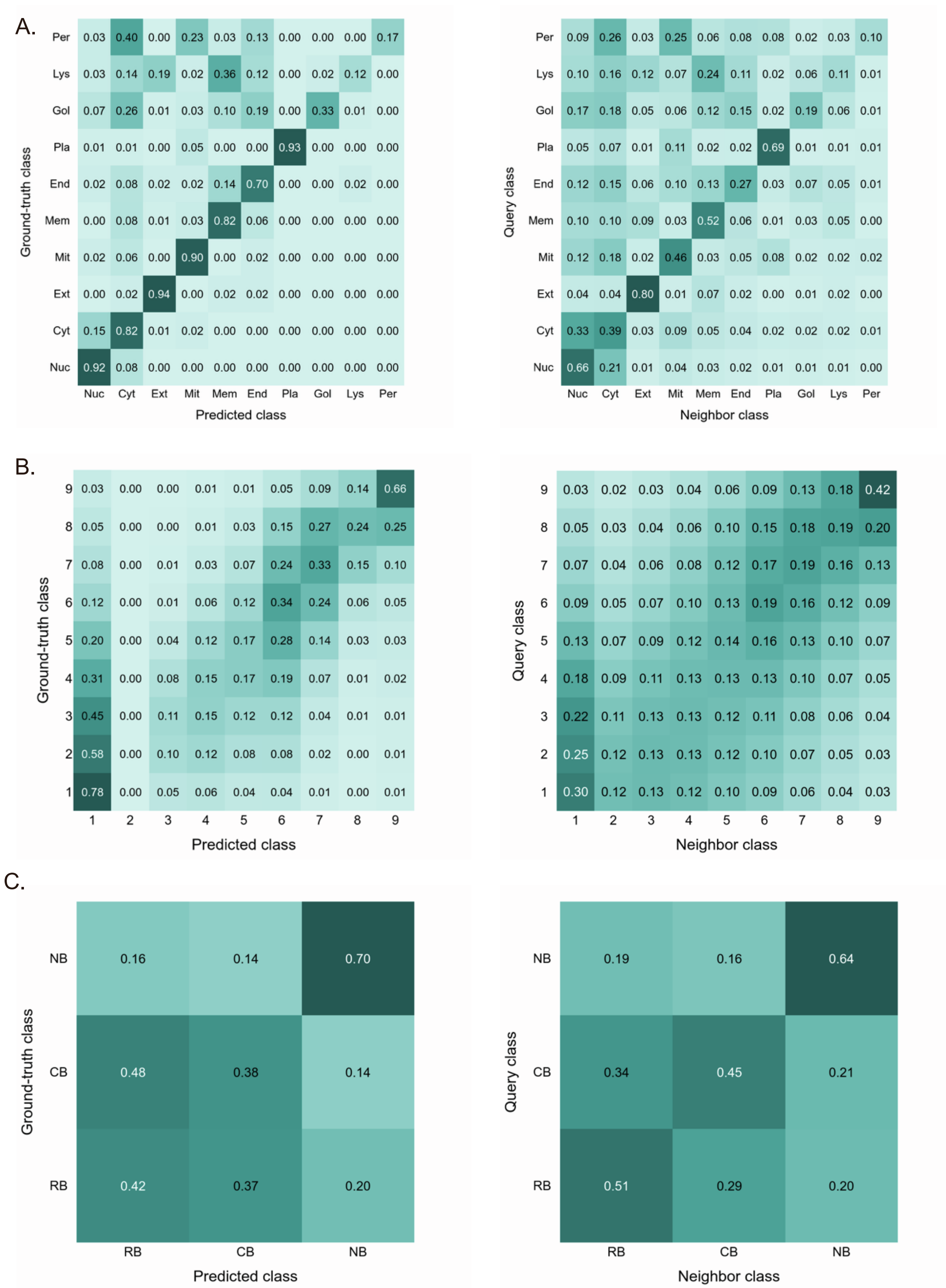

Figure S4: Fractional class distributions from transfer learning and embedding space neighborhood analysis across three tasks.

For each task, the left panel shows the predicted class distribution from the transfer learning model, where values indicate the fraction of predictions falling in each predicted class for each ground-truth class (x-axis: predicted class, y-axis: ground-truth class). The right panel shows the normalized KNN class mixing matrix, where values indicate the proportion of k-nearest neighbors belonging to each neighbor class out of all neighbors of the query class (x-axis: neighbor class, y-axis: query class), normalized per query class row, based on k = 100, cosine distance, after mean centering. **(A) Subcellular localization.** ProtT5 embedding space. Transfer learning performance as reported by Stärk et al. [S2]. Cyt: Cytoplasm; End: Endoplasmic reticulum; Ext: Extracellular; Gol: Golgi apparatus; Lys: Lysosome/vacuole; Mem: Cell membrane; Mit: Mitochondrion; Nuc: Nucleus; Per: Peroxisome; Pla: Plastid. **(B) Residue conservation.** ProtT5 embedding space. Transfer learning performance as reported by Marquet et al. [S4]. Conservation categories 1–9 range from highly variable to highly conserved. **(C) Ligand binding site (three-class).** ProstT5 embedding space, selected as the best-performing embedding space for this task. Transfer learning performance from the MLP classifier trained in this study. NB: non-binding; RB: regular binding; CB: cryptic binding.

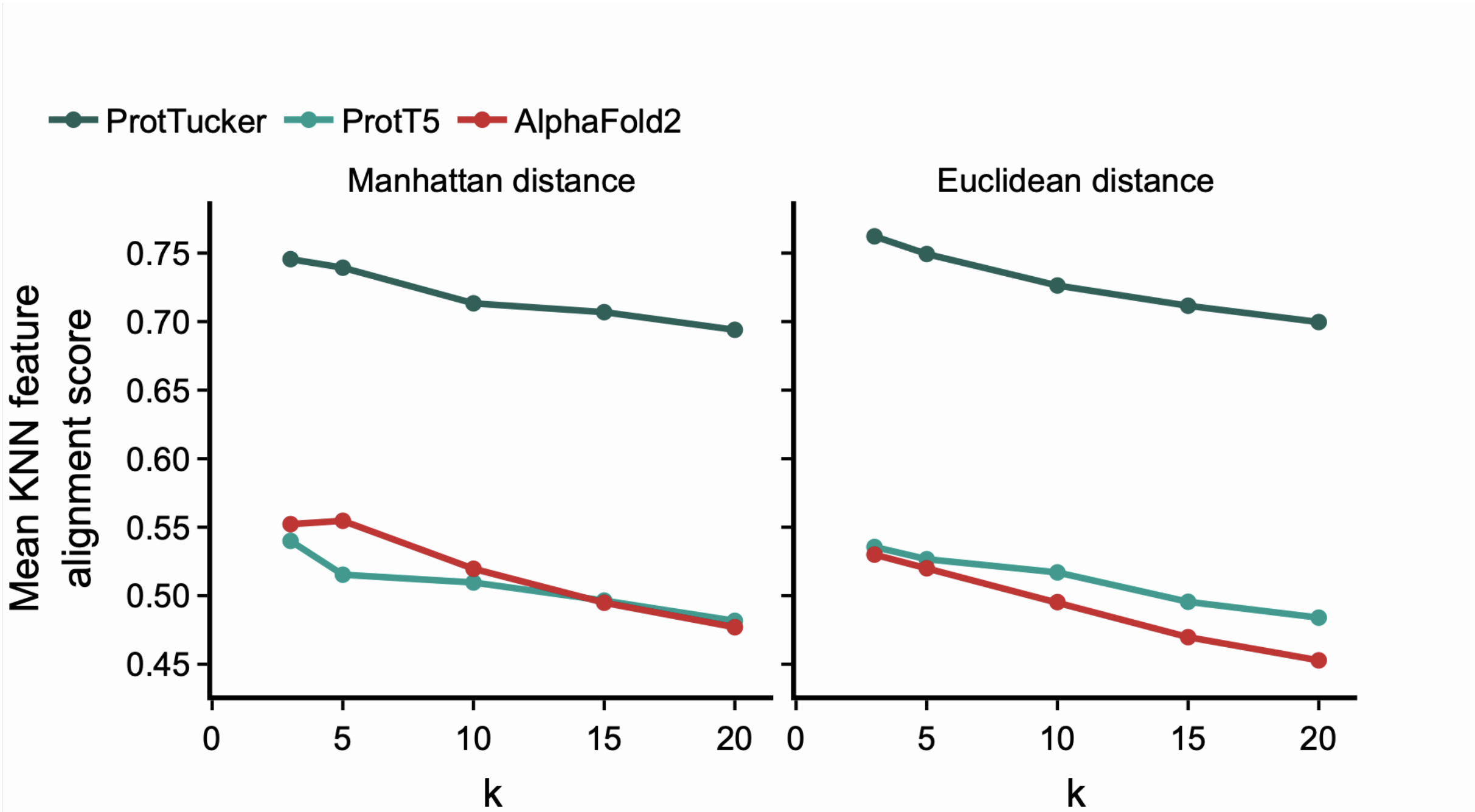

Figure S5: Mean KNN feature alignment scores for the CATH class feature across Manhattan and Euclidean distance metrics.
Mean KNN feature alignment scores (y-axis) for CATH class feature across varying neighborhood sizes (k, x-axis) for ProtTucker, ProtT5, and AlphaFold2, shown for Manhattan (left panel) and Euclidean distance (right panel) after mean centering. ProtTucker consistently ranks highest across both distance metrics, confirming that the ranking pattern observed with cosine distance in Figure 5A is robust to the choice of distance metric.

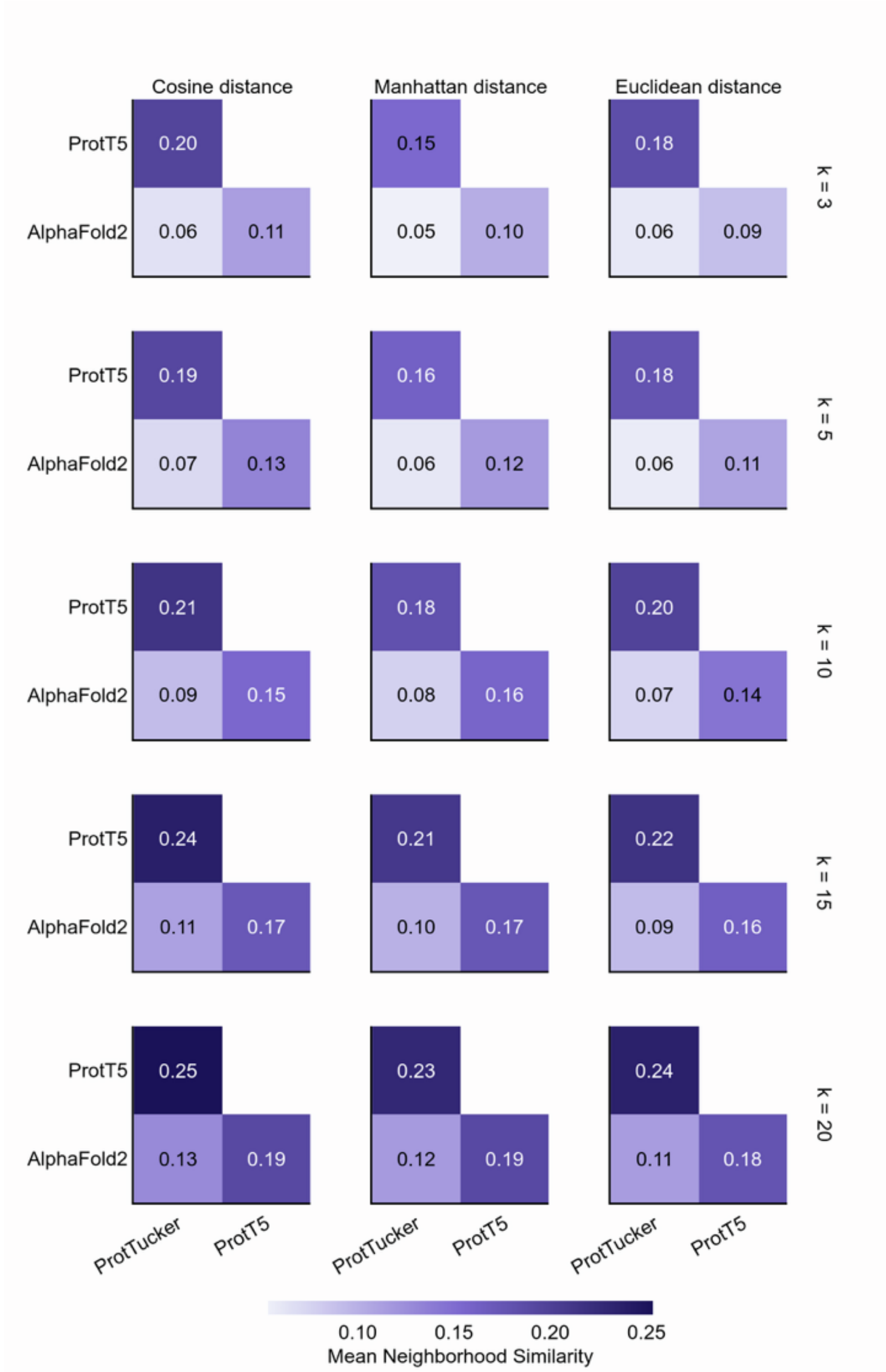

Figure S6: Mean cross-space neighborhood similarity matrices between ProtTucker, ProtT5, and AlphaFold2 across k values and distance metrics.
Mean cross-space neighborhood similarity between all pairs of embedding models (ProtTucker, ProtT5, AlphaFold2), shown as 2×2 matrices for five k values (k = 3, 5, 10, 15, 20; rows) and three distance metrics (cosine, Manhattan, Euclidean; columns). Each cell shows the mean neighborhood similarity for the corresponding model pair at that k value and distance metric; diagonal cells are excluded as self-similarity is uninformative. Across all k values and distance metrics, the ProtTucker–ProtT5 pair consistently shows the highest neighborhood similarity, reflecting the preservation of core representational geometry following contrastive fine-tuning.

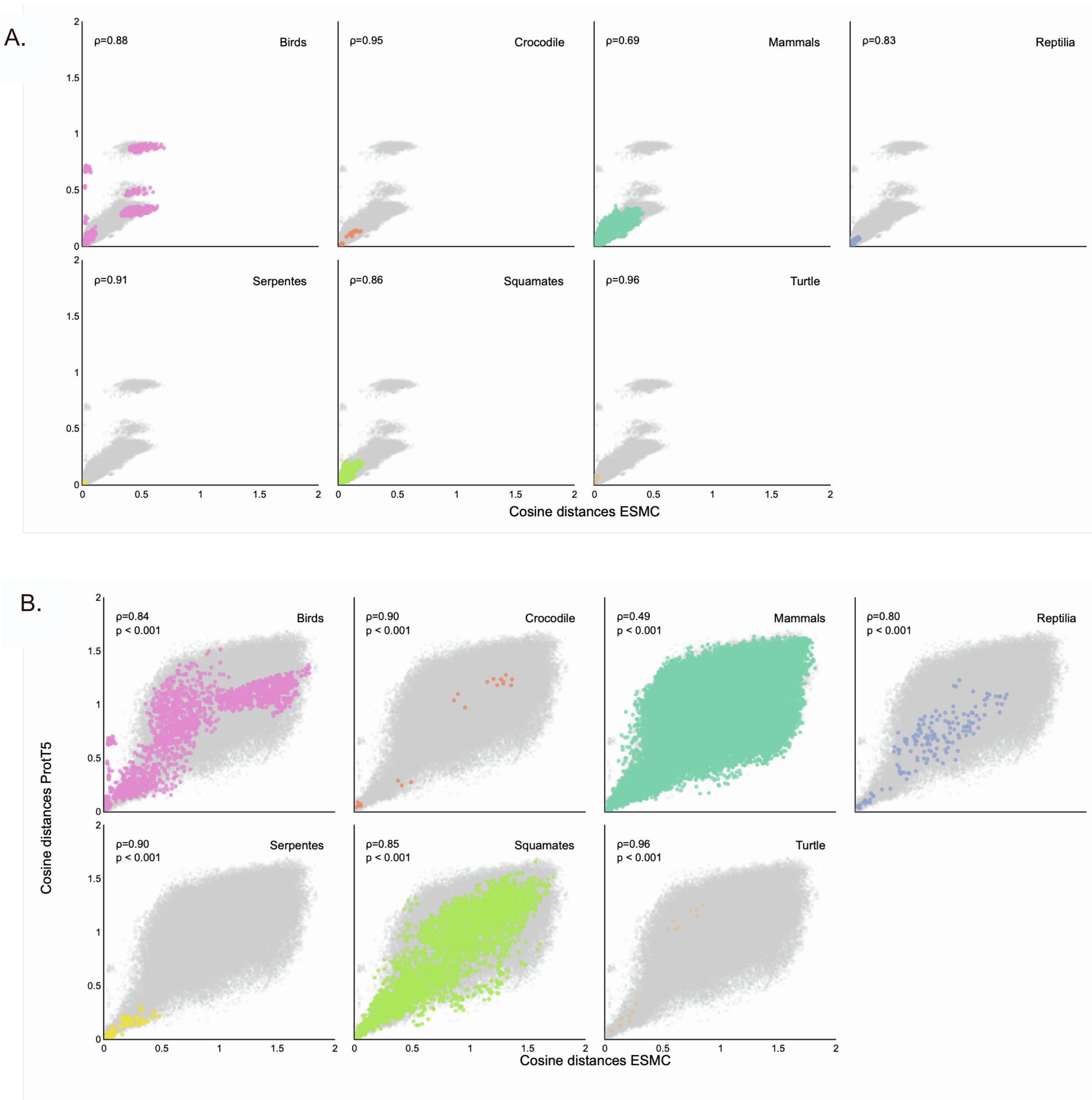

Figure S7: Pairwise cosine distances between PLA2G2 protein embeddings in ESM C and ProtT5, colored by species, before and after mean centering.

Pairwise cosine distances between PLA2G2 protein embeddings in ESM C (x-axis) and ProtT5 (y-axis) for all pairs (grey) and for pairs belonging to the same species (colored points; one panel per species). (A) Before mean centering. A visually distinct divergent cluster is apparent, predominantly composed of pairs from bird species (pink), suggesting a species-level representational difference between the two models. (B) After mean centering. The bird protein cluster is less apparent, indicating that the divergence observed prior to correction is a geometric artifact of anisotropy rather than a genuine representational difference between the models.

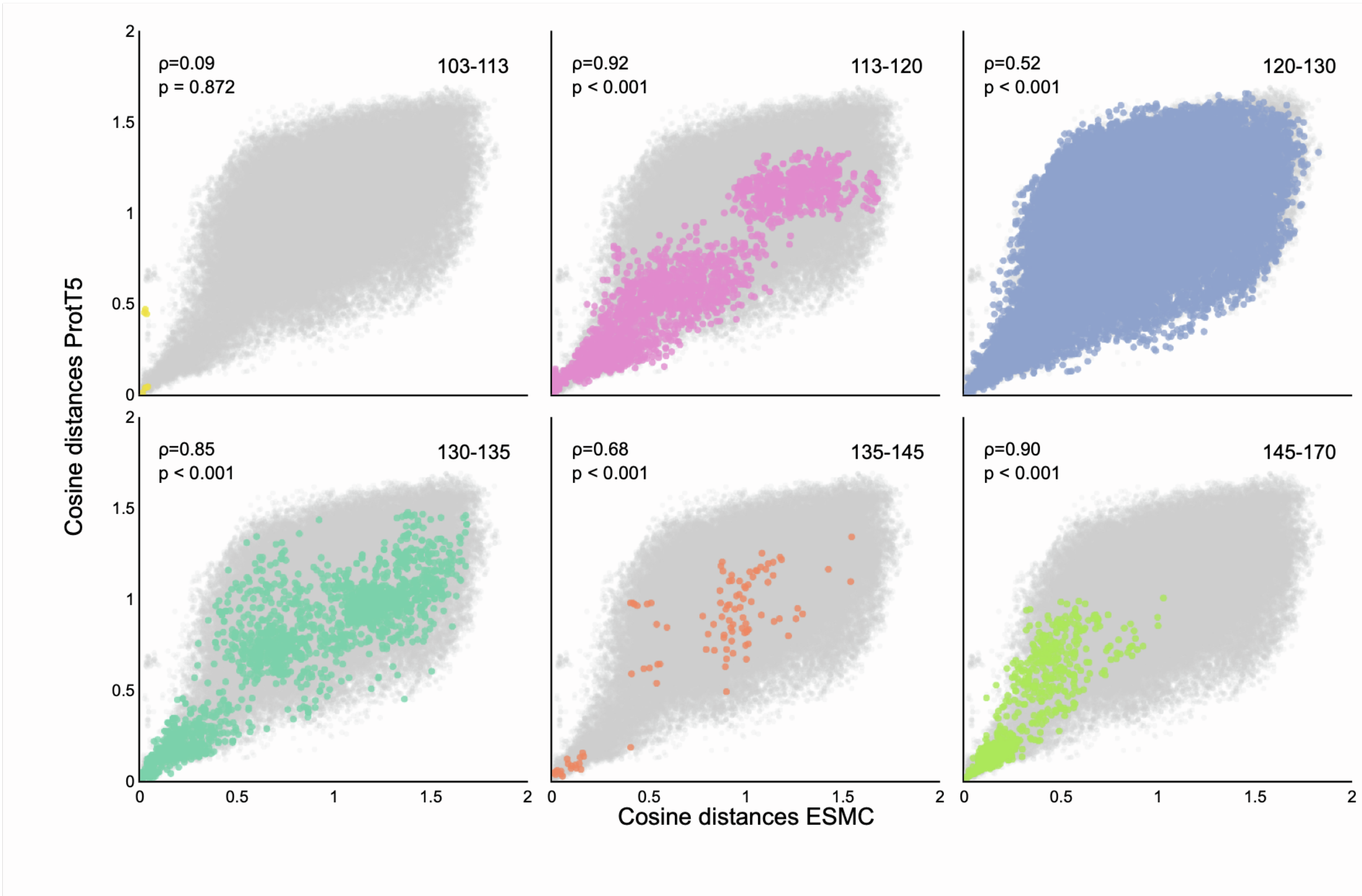

Figure S8: Pairwise cosine distances after mean centering between PLA2G2 protein embeddings in ESM C and ProtT5, colored by binned sequence length.
Pairwise cosine distances after mean centering between PLA2G2 protein embeddings in ESM C (x-axis) and ProtT5 (y-axis) for all pairs (grey) and for pairs falling within the same sequence length bin (colored points; one panel per bin).

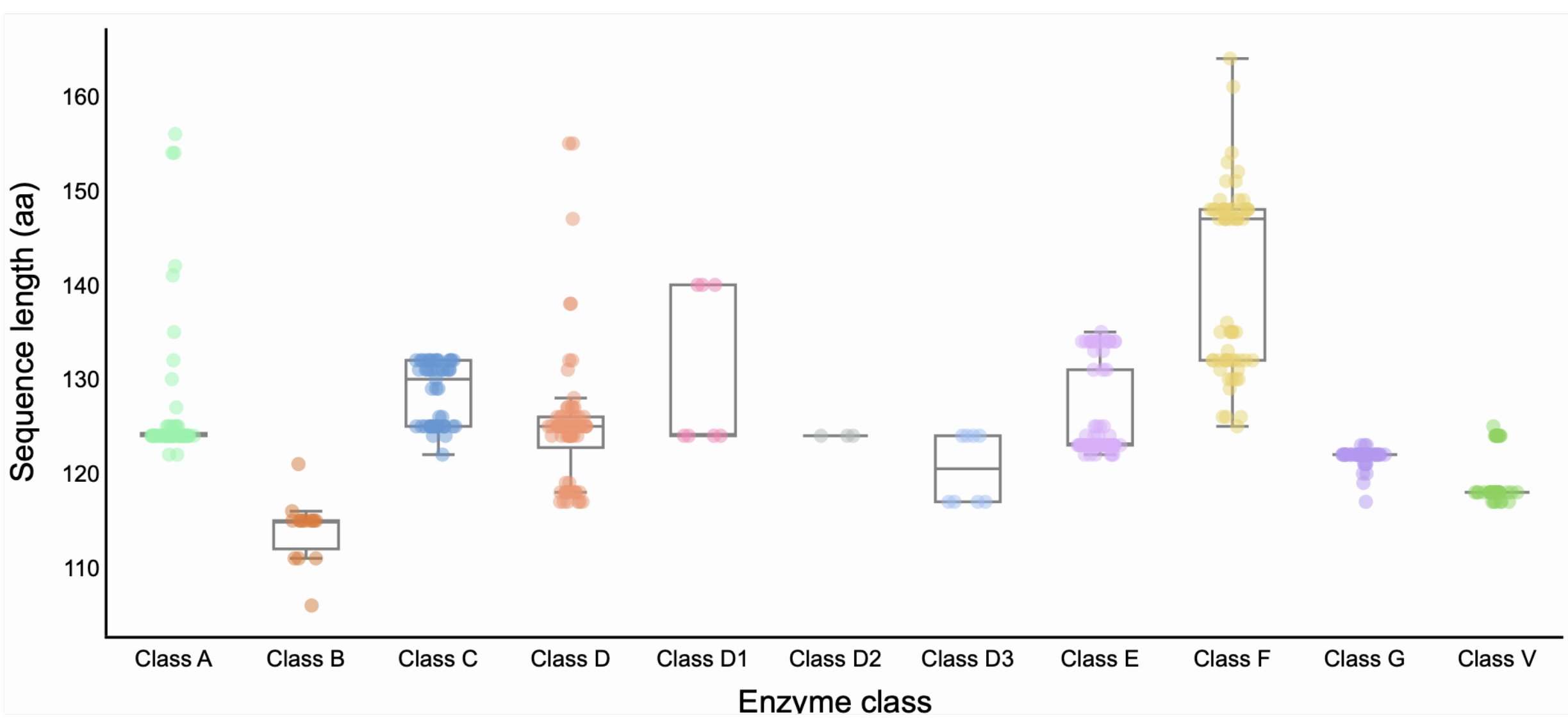

Figure S9: Sequence length distribution per PLA2G2 enzyme class.
Box plots showing the distribution of protein sequence length (y-axis) across PLA2G2 enzyme classes (x-axis).

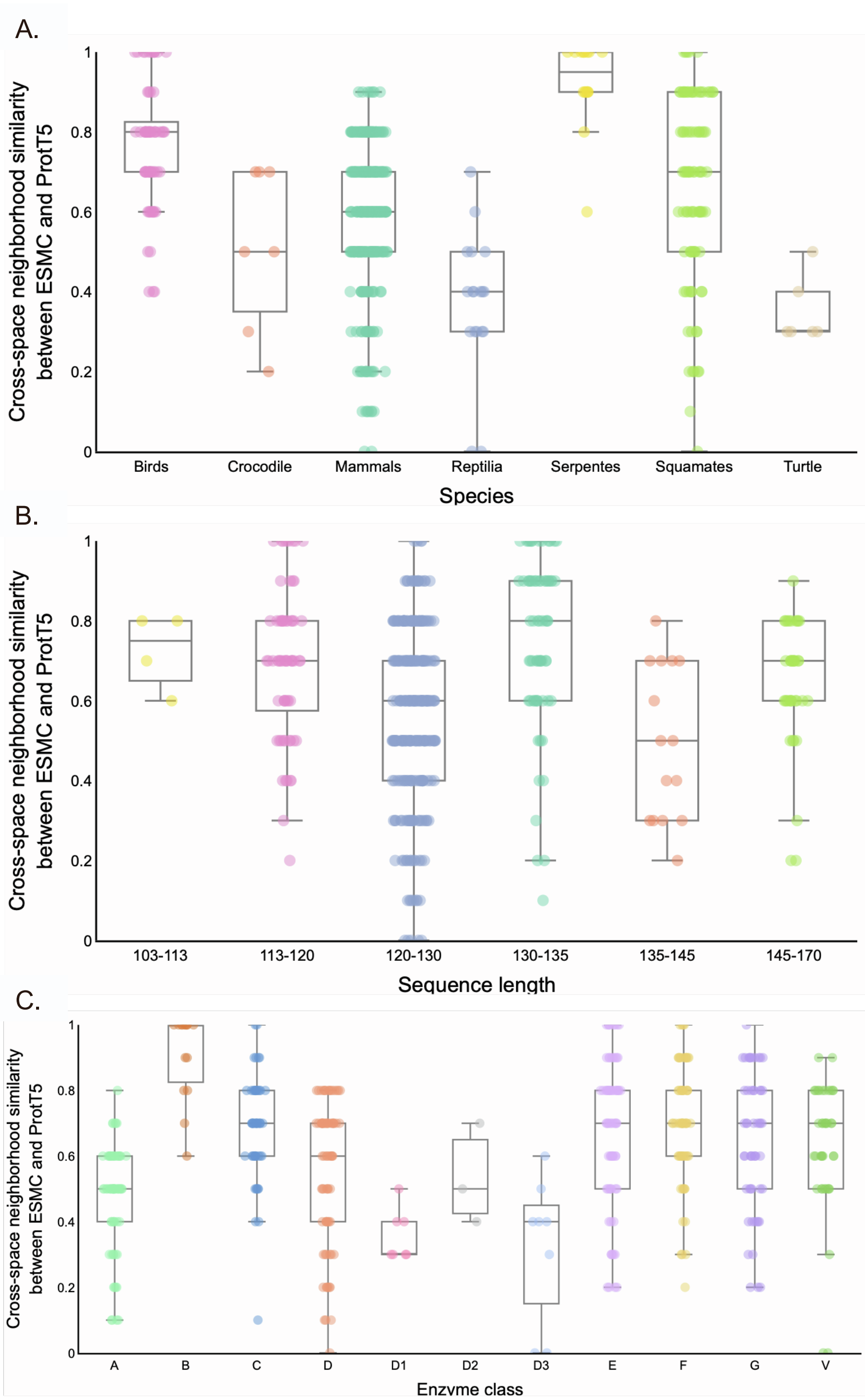

Figure S10: Cross-space neighborhood similarity between ESM C and ProtT5 embeddings of PLA2G2 proteins, stratified by species, sequence length, and enzyme class.
Cross-space neighborhood similarity (y-axis; cosine distance with k = 10) between ESM C and ProtT5 embeddings after mean centering for individual PLA2G2 proteins, shown as box plots with overlaid individual data points, stratified by (A) taxonomic group (Birds, Crocodile, Mammals, Reptilia, Serpentes, Squamates, Turtle), (B) sequence length bin, and (C) enzyme class.

Supplemental Tables

Table S1: Protein counts per subcellular localization class in the subcellular localization dataset

**Class**	**Protein count**	**Percentage**
Nucleus	3,235	28.8%
Cytoplasm	2,180	19.4%
Extracellular	1,580	14.1%
Mitochondrion	1,208	10.8%
Membrane	1,067	9.5%
Endoplasmic reticulum	689	6.1%
Plastid	605	5.4%
Golgi apparatus	286	2.6%
Lysosome/ Vacuole	257	2.3%
Peroxisome	124	1.1%
Total	11,231	

Table S2: Residue counts per conservation class in the residue conservation dataset

**Class**	**Residue count**	**Percentage**
1-conservation	311,365	13.0%
2-conservation	160,915	6.7%
3-conservation	205,712	8.6%
4-conservation	246,379	10.3%
5-conservation	283,649	11.8%
6-conservation	334,004	13.9%
7-conservation	315,853	13.2%
8-conservation	266,929	11.1%
9-conservation	273,931	11.4%
**Total**	**2,398,737**	

Table S3: Residue counts per residue binding class in the residue binding (binary) dataset

**Class**	**Residue count**	**Percentage**
Non-binding	156,687	91.8%
Binding	13,999	8.2%
**Total**	**170,686**	

Table S4: Protein counts per CATH class in the protein structure (CATH) dataset

Class	Protein count	Percentage
A-1	93	31.0%
A-2	77	25.7%
A-3	123	41.0%
A-4	6	2.0%
A-6	1	0.3%
**Total**	**300**	

Table S5: Protein counts per class in the Phospholipase A2 family (PLA2G2) dataset

Feature	Class	Protein count	Percentage
Species	mammals	250	56.1%
	squamates	88	19.7%
	birds	61	13.7%
	reptilia	18	4.0%
	serpentes	16	3.6%
	crocodile	7	1.6%
	turtle	6	1.3%
Enzyme class	D	73	16.4%
	E	70	15.7%
	A	65	14.6%
	F	61	13.7%
	G	53	11.9%
	C	51	11.4%
	V	40	9.0%
	B	15	3.4%
	D3	8	1.8%
	D1	7	1.6%
	D2	3	0.7%
Sequence length	120-130	257	57.6%
	130-135	67	15.0%
	113-120	65	14.6%
	145-170	38	8.5%
	135-145	15	3.4%
	103-113	4	0.9%
Total		446	

Table S6: Residue counts per ligand binding class in the ligand binding site (three-class) dataset

**Dataset**	**Class**	**Residue count**	**Percentage**
Train	Non-binding	12,964	33.3%
	Cryptic binding	12,964	33.3%
	Regular binding	12,970	33.3%
	**Total**	**38,898**	
Test	Non-binding	3,099	33.2%
	Cryptic binding	3,099	33.1%
	Regular binding	3,138	33.6%
	**Total**	**9,336**	

Table S7: Overview of protein language model benchmark tasks, datasets, and reference

Task	Task description	Prediction level	Number of classes	Training and test datasets	Redundancy reduction	Model architecture	Performance measure
Subcellular localization	Predicting the subcellular localization of a protein	Per protein	10	train: DeepLoc training data [S1]; test: setHARD [S2]	Test set was constructed independently from a different data source than the training data and filtered at 20% sequence identity to remove all sequences homologous to training set proteins	Light-attention [S2]	Q10: 10-state per-protein accuracy
Residue conservation	Predicting the conservation of a protein residue	Per residue	9 (1 = highly variable to 9 = highly conserved)	train and test: ConSurf10k [S3, S4]	Redundancy reduction at 25% sequence identity was applied to the full dataset prior to random partitioning into train, validation, and test sets	2-layer CNN [S4]	Q9: nine-state per-residue accuracy
Residue binding (binary)	Predicting binding of a protein to ligands (small molecule, metal ion, or nucleotide)	Per residue	2 (binding vs non-binding)	BioLip [S5]; train and test splits as in [S6]	Redundancy reduction at less than 20% pairwise sequence identity over 250 aligned residues was applied to the full dataset prior to splitting into development and test sets	2-layer CNN [S6]	F1 score
Ligand binding site (three-class)	Predicting cryptic binding residues, regular binding residues and non-binding residues	Per residue	3	Train: scPDB-enhanced [S7], CryptoBench train set [S8]; Test: LIGYSIS [S9, S10], CryptoBench test set [S8]	Controlled sequence similarity down to 10%	3-layer MLP	AUPRC

CNN: convolutional neural network; MLP: multilayer perceptron

Table S8: Performance of transfer learning model for ligand binding site (three-class)

Model	Accuracy	AUC	MCC	F1	AUPRC
Ankh	0.486	0.727	0.231	0.484	0.559
ProtT5	0.486	0.733	0.234	0.486	0.565
ProstT5	0.499	0.739	0.249	0.497	0.585
ESM2	0.448	0.677	0.178	0.425	0.500

Table S9: Embedding space geometry diagnostics for all model-dataset combinations

Dataset	Model	AvgCosine (raw)	AvgCosine (centered)	Robin Hood score (raw)	Robin Hood score (centered)	N90 Dim	Mean centering applied
Subcellular localization	Ankh	0.437	0.003	0.289	0.257	1224 out of 1536	True
	ESM2	0.956	0.005	0.389	0.326	1983 out of 2560	True
	ProstT5	0.330	0.001	0.258	0.248	859 out of 1024	True
	ProtT5	0.663	0.009	0.270	0.270	799 out of 1024	True
Residue conservation	Ankh	0.027	0.001	0.330	0.324	1247 out of 1536	True
	ESM2	0.689	0.003	0.958	0.494	2203 out of 2560	True
	ProstT5	0.037	-0.001	0.292	0.290	825 out of 1024	True
	ProtT5	0.015	-0.000	0.296	0.296	868 out of 1024	True
Residue binding (binary)	Ankh	0.026	0.000	0.264	0.258	1248 out of 1536	True
	ESM2	0.717	0.002	0.560	0.268	2201 out of 2560	True
	ProstT5	0.041	0.001	0.255	0.250	824 out of 1024	True
	ProtT5	0.016	0.000	0.224	0.222	864 out of 1024	True
Ligand binding site (three-class)	Ankh	0.038	0.000	0.265	0.254	1242 out of 1536	True
	ESM2*	0.356	0.003	0.479	0.393	1086 out of 1280	True
	ProstT5	0.045	0.001	0.231	0.228	828 out of 1024	True
	ProtT5	0.021	0.001	0.233	0.228	861 out of 1024	True
CATH	ProtT5	0.278	0.003	0.310	0.220	864 out of 1024	True
	ProtTucker	0.853	0.001	0.423	0.244	113 out of 128	True
	AlphaFold2	0.975	0.011	0.344	0.267	189 out of 256	True
PLA2G2	ESM C	0.920	0.021	0.252	0.264	574 out of 960	True
	ProtT5	0.894	0.005	0.270	0.264	704 out of 1024	True

AvgCosine (raw) = average pairwise cosine similarity before mean-centering; AvgCosine (centered) = average pairwise cosine similarity after mean-centering; N90 Dim = number of dimensions capturing 90% of total variance before mean centering; * For ligand binding sites (three-class), a different ESM-2 model version was used: esm2_t33_650M_UR50D. All other tasks used esm2_t36_3B_UR50D.

Table S10: One-sided Wilcoxon signed-rank tests for KNN feature alignment scores at k = 100 for the subcellular localization dataset after mean centering of the embeddings.
P-values shown as 0.000000e+00 are smaller than machine precision (p < 2.22e-16).

**Distance metric**	**Model 1**	**Model 2**	**p**	**Rank biserial correlation**
Cosine	ProtT5	Ankh	2.045e-284	0.408
Cosine	ProtT5	ESM2	0.000e+00	0.496
Cosine	Ankh	ESM2	9.259e-11	0.072
Cosine	Ankh	ProstT5	3.047e-235	0.371
Cosine	ESM2	ProstT5	9.710e-85	0.219

Table S11: One-sided Wilcoxon signed-rank tests for KNN feature alignment scores at k = 100 for the residue conservation dataset after mean centering of the embeddings.
P-values shown as 0.000000e+00 are smaller than machine precision (p < 2.22e-16).

**Distance metric**	**Model 1**	**Model 2**	**p**	**Rank biserial correlation**
Cosine	Ankh	ESM2	0.000000e+00	0.050
Cosine	ESM2	ProtT5	0.000000e+00	0.228
Cosine	ProtT5	ProstT5	0.000000e+00	0.203

Table S12: One-sided Wilcoxon signed-rank tests for KNN feature alignment scores at k = 100 for the residue binding (binary) dataset after mean centering of the embeddings.

**Distance metric**	**Model 1**	**Model 2**	**p**	**Rank biserial correlation**
Cosine	Ankh	ProtT5	4.374e-95	0.063
Cosine	ProtT5	ProstT5	5.731e-174	0.085
Cosine	ProstT5	ESM2	1.154e-05	0.013

Table S13: One-sided Wilcoxon signed-rank tests for KNN feature alignment scores at k = 100 for the ligand binding site (three-class) dataset after mean centering of the embeddings.

**Distance metric**	**Model 1**	**Model 2**	**p**	**Rank biserial correlation**
Cosine	ProstT5	ProtT5	1.617e-49	0.088
Cosine	ProtT5	Ankh	1.808e-68	0.104
Cosine	Ankh	ESM2	9.189e-182	0.171

Table S14: One-sided Wilcoxon signed-rank tests for KNN feature alignment scores at k = 20 for the protein structure (CATH) dataset after mean centering of the embeddings.

Distance metric	Model 1	Model 2	p	Rank biserial correlation
Cosine	ProtTucker	AlphaFold2	1.541e-36	0.767
Cosine	ProtTucker	ProtT5	6.471e-37	0.871
Cosine	ProtT5	AlphaFold2	0.0009	0.221

Table S15: One-sided Wilcoxon signed-rank tests for neighborhood similarity scores at k = 20 for the protein structure (CATH) dataset after mean centering of the embeddings.

Distance metric	Model pair 1	Model pair 2	p	Rank biserial correlation
Cosine	ProtTucker to ProtT5	ProtT5 to AlphaFold2	5.353e-09	0.405
Cosine	ProtT5 to AlphaFold2	ProtTucker to AlphaFold2	1.411e-23	0.708

Supplemental Notes

S1: EmmaEmb guided diagnostic workflow for embedding space ranking

Steps 1-2 characterize the geometric properties of the embedding space and are run once per embedding space prior to any feature-specific analysis. Steps 3-6 are run separately for each feature of interest, as class distribution, k range, and robustness assessments depend on the specific feature being analyzed.

A worked example of Step 1 and the full workflow is provided as an interactive Jupyter notebook, viewable and executable in Google Colab within the EmmaEmb github repository. The notebook demonstrates each step on an example dataset and can be adapted for user-provided embedding spaces and metadata.

Step 1: Anisotropy assessment

Tests:

- Compute average pairwise cosine similarity between randomly sampled embedding pairs (following [S11]) on raw and mean-centered embeddings
- Compute per-dimension variance to identify dominant dimensions

Decision:

- if average cosine similarity drops substantially after mean centering → mean-offset anisotropy detected → apply mean centering to all subsequent steps after step 2
- if average cosine similarity remains high after mean centering → structural anisotropy → recommend cosine distance; flag results for caution
- A small number of dimensions account for a disproportionate fraction of total variance → dimension dominance detected:
    - avoid Euclidean and Manhattan distances; distances will be driven by dominant dimensions regardless of biological signal in remaining dimensions
    - cosine distance is more robust but not immune; if dominance persists after mean centering, interpret all distance-based results with caution

Output: processed embeddings (raw or mean-centered); recommended distance metric; dimension variance profile

Step 2: Hubness assessment

Tests:

- Compute k-occurrence distribution: count how many times each point appears as a nearest neighbor of others (following [S12]) and visualize skewness of k-occurrence distribution
- Compute Robin Hood index (RHI): fraction of total occurrences that would need to be redistributed from hubs to equalize the distribution [S13], run at k = 5, 10, 20 to confirm stability of hubness estimate - Compare RHI across models, not against absolute thresholds, a large spread in RHI across models on the same dataset indicates that KNN scores are geometrically less comparable between models, which could distort relative rankings independently of biological signal

Note that hubness tends to increase with embedding dimensionality [S12], a high RHI for a high-dimensional model may partly reflect dimensionality rather than learned representation properties; reporting effective rank or intrinsic dimensionality alongside RHI helps disambiguate

Decisions:

- RHI drops substantially after mean centering, and values are low and comparable across models → hubness was primarily driven by anisotropy and has been addressed by mean centering → no contraindicating measures against proceeding with KNN-based analysis
- RHI drops after mean centering, but one or more models remain substantially more hubbed than others → asymmetric residual hubness remains → flag affected models; rankings for those models should be interpreted with additional caution; cross-model comparisons may not be geometrically equivalent
- RHI remains high and comparable across all models after mean centering → structural or dimensionality-driven hubness → no contraindicating measures against proceeding, but results should be interpreted with caution; prefer smaller k values within the range set in Step 3; note that KNN scores may underestimate true representational differences for all models equally, which preserves relative rankings but reduces absolute interpretability
- RHI is high for some models but not others after mean centering, and the spread is large → asymmetric hubness not resolved by centering → flag strongly; relative embedding space rankings are potentially unreliable; consider whether the analysis question can be reframed to reduce dependence on local neighborhood structure
- RHI is low and comparable across all models on raw embeddings with no substantial change after centering → no contraindicating measures against proceeding

Output: RHI before and after centering per model; cross-model spread; flag indicating whether hubness concern is resolved, partially resolved, or unresolved; recommended k ceiling adjustment if applicable

Step 3: Class distribution and k range

Tests:

- Count samples per class; compute class size distribution
- Compute imbalance ratio (largest class / smallest class)
- Compute expected random baseline alignment score per class: $n\_class / (N - 1)$, scores should be interpreted relative to this baseline throughout

Determining k value:

- Hard ceiling: $k < n\_min$ (smallest class size): if $k \geq n\_min$, alignment scores decrease trivially rather than reflecting embedding structure
- Soft ceiling: $k \leq \sqrt{N}$ as a general heuristic; for datasets where $\sqrt{N}$ approaches n_min $k \leq 5\%$ of N
- Tighter ceiling if high hubness detected in Step 2: $k \leq \sqrt{n\_min}$
- Range construction: rather than testing a single k value, the analysis is run across a progressive range of k values from the lower bound to the upper bound, for example k = 1, 2, 5, 10, 20, 50, 100 or similar geometric progression, so that the stability of rankings across neighborhood scales can be assessed directly. The specific steps within the range are chosen to be informative at both small and large scales rather than uniformly spaced, since neighborhood structure tends to change most rapidly at small k values
- If the ANNOY approximate nearest neighbor algorithm was used: account for the fact that ANNOY returns approximate rather than exact neighbors, which introduces a small degree of noise into the k-occurrence counts and alignment scores. The k range used should be reported alongside the number of trees used in the ANNOY index, and results at very small k values ($k < 5$) should be interpreted with additional caution since approximation errors are proportionally larger relative to the neighborhood size

Flag missing data: columns with a high proportion of missing values are flagged; a default threshold of 70% is used as a practical starting point, which users may adjust depending on their dataset.

Decision:

- datasets with severe class imbalance (a default imbalance ratio of 10 is used as a practical indicator, though the appropriate threshold is context-dependent) → flag as highly imbalanced → Step 5 rebalancing analysis required

Output: k range; expected random baseline per class; imbalance flag; missing data flags

Step 4: Parameter sensitivity analysis

Test:

- Ranking stability plot: Run KNN alignment scores across full k range from Step 3. Run across all three distance metrics (cosine, Euclidean, Manhattan) even if one is recommended, sensitivity across metrics is itself informative; Produce ranking stability plot: model scores (y) vs k (x), one line per model, one panel per metric

Decision:

- Consistent embedding space rankings across full k range and all metrics → high confidence in model prioritization
- Rankings consistent across k but sensitive to distance metric → report cosine results as primary; note metric sensitivity
- Rankings crossing or inverting across k range → conclusions are parameter-sensitive → report full range; do not report single k

Output: ranking stability plot; confidence flag (stable / metric-sensitive / parameter-sensitive)

Step 5: Class imbalance robustness (run if flagged in Step 3)

Test:

- Rebalancing stability plot: Progressive rebalancing: down sample majority class toward n_min in steps; repeat each ratio multiple times for stable estimates; Produce rebalancing stability plot: model scores (y) vs imbalance ratio (x)

Decision:

- Consistent rankings across all subsampling ratios → imbalance is not driving the result → report original rankings with confidence
- Rankings change substantially under rebalancing → original ranking was frequency-driven, not structure-driven → balanced sampling gives more trustworthy estimate; report rebalanced rankings as primary
- Rankings inconsistent across subsampling levels → neighborhood-based model prioritization is not reliable for this dataset → flag; do not report a embedding space ranking

Step 6: Feature noise robustness

Test:

- Noise stability plot: Progressively corrupt a fraction of annotations randomly (0% to 100% in steps); keep embeddings fixed; recompute embedding space rankings at each noise level; produce noise stability plot: embedding space rankings (y) vs noise level (x)
- Report crossing point: the noise level at which embedding space rankings first become unreliable

Decision:

- Rankings are consistent across all noise levels up to the point where annotations are substantially randomized → strong evidence that conclusions are robust to annotation uncertainty; the crossing point can be reported as a lower bound on the annotation quality required for the ranking to hold

- Rankings change at low noise levels → conclusions are sensitive to metadata quality; the crossing point gives a concrete, interpretable statement about required annotation quality: rankings are only reliable if annotation error rate is below the crossing point

References

S1. Almagro Armenteros, J.J., Sønderby, C.K., Sønderby, S.K., Nielsen, H., and Winther, O. (2017). DeepLoc: prediction of protein subcellular localization using deep learning. Bioinformatics *33*, 3387–3395. https://doi.org/10.1093/bioinformatics/btx431.

S2. Stärk, H., Dallago, C., Heinzinger, M., and Rost, B. (2021). Light attention predicts protein location from the language of life. Bioinformatics Advances *1*, vbab035. https://doi.org/10.1093/bioadv/vbab035.

S3. Ben Chorin, A., Masrati, G., Kessel, A., Narunsky, A., Sprinzak, J., Lahav, S., Ashkenazy, H., and Ben-Tal, N. (2020). ConSurf-DB: An accessible repository for the evolutionary conservation patterns of the majority of PDB proteins. Protein Sci *29*, 258–267. https://doi.org/10.1002/pro.3779.

S4. Marquet, C., Heinzinger, M., Olenyi, T., Dallago, C., Erckert, K., Bernhofer, M., Nechaev, D., and Rost, B. (2022). Embeddings from protein language models predict conservation and variant effects. Hum Genet *141*, 1629–1647. https://doi.org/10.1007/s00439-021-02411-y.

S5. Yang, J., Roy, A., and Zhang, Y. (2012). BioLiP: a semi-manually curated database for biologically relevant ligand–protein interactions. Nucleic Acids Research *41*, D1096–D1103. https://doi.org/10.1093/nar/gks966.

S6. Littmann, M., Heinzinger, M., Dallago, C., Weissenow, K., and Rost, B. (2021). Protein embeddings and deep learning predict binding residues for various ligand classes. Sci Rep *11*, 23916. https://doi.org/10.1038/s41598-021-03431-4.

S7. Vít Škrhák (2026). scPDB-Enhanced: An expansion of scPDB including all experimentally observed binding sites. https://doi.org/https://doi.org/10.5281/zenodo.18271517 https://doi.org/https://doi.org/10.5281/zenodo.18271517.

S8. Škrhák, V., Novotný, M., Feidakis, C.P., Krivák, R., and Hoksza, D. (2025). CryptoBench: cryptic protein–ligand binding sites dataset and benchmark. Bioinformatics *41*, btae745. https://doi.org/10.1093/bioinformatics/btae745.

S9. Utgés, J.S., and Barton, G.J. (2024). Comparative evaluation of methods for the prediction of protein–ligand binding sites. J Cheminform *16*, 126. https://doi.org/10.1186/s13321-024-00923-z.

S10. Utgés, J.S., MacGowan, S.A., and Barton, G.J. (2025). LIGYSIS-web: a resource for the analysis of protein-ligand binding sites. Nucleic Acids Res *53*, W351–W360. https://doi.org/10.1093/nar/gkaf411.

S11. Ethayarajh, K. (2019). How Contextual are Contextualized Word Representations? Comparing the Geometry of BERT, ELMo, and GPT-2 Embeddings. In Proceedings of the 2019 Conference on Empirical Methods in Natural Language Processing and the 9th International Joint Conference on Natural Language Processing (EMNLP-IJCNLP), K. Inui, J. Jiang, V. Ng, and X. Wan, eds. (Association for Computational Linguistics), pp. 55–65. https://doi.org/10.18653/v1/D19-1006.

S12. Radovanovic, M., Nanopoulos, A., and Ivanovic, M. (2010). Hubs in space: Popular nearest neighbors in high-dimensional data. Journal of machine learning research *11*, 2487–2531.

S13. Feldbauer, R., Leodolter, M., Plant, C., and Flexer, A. (2018). Fast Approximate Hubness Reduction for Large High-Dimensional Data. In 2018 IEEE International Conference on Big Knowledge (ICBK), pp. 358–367. https://doi.org/10.1109/ICBK.2018.00055.
