# Supplementary material for: EmmaEmb: A quantitative framework for analyzing embedding spaces in molecular biology
Source: Patterns (N Y). 2026 Aug 12;7(9):101635. doi: 10.1016/j.patter.2026.101635 (PMC13576657; doi:10.1016/j.patter.2026.101635)
Supplement: Supplementary file 2 [file mmc2.pdf]

Article

Patterns

EmmaEmb: A quantitative framework for analyzing embedding spaces in molecular biology

Highlights

- EmmaEmb is a computational framework to analyze and compare biological embedding spaces
- Embedding analyses rank protein model representations by task relevance without training
- Embedding-space neighborhoods reveal misclassification patterns in transfer learning
- EmmaEmb quantifies representational changes induced by model fine-tuning

Authors

Pia Francesca Rissom, Vít Škrhák, Paulo Yanez Sarmiento, ..., Bernhard Y. Renard, Henrike O. Heyne, Sumaiya Iqbal

Correspondence

francesca.rissom@hpi.de (P.F.R.), henrike.heyne@hpi.de (H.O.H.), sumaiya@broadinstitute.org (S.I.)

In brief

Foundation models generate embeddings that capture complex biological information, but interpreting and comparing these representations remains challenging. Rissom et al. introduce EmmaEmb, a model-agnostic framework for the analysis of embedding spaces. Applied to protein language models and high-stakes molecular biology and drug-discovery tasks, EmmaEmb ranks representations by task relevance, reveals patterns underlying prediction errors, and tracks representational changes during model adaptation, providing a quantitative approach to understanding learned biological representations. All methods are available as an open-source Python library.

Rissom et al., 2026, Patterns 7, 101635
September 11, 2026 © 2026 The Authors. Published by Elsevier Inc.
https://doi.org/10.1016/j.patter.2026.101635

Article

EmmaEmb: A quantitative framework for analyzing embedding spaces in molecular biology

Pia Francesca Rissom,[1,2,*] Vít Škrhák,[2,3,11] Paulo Yanez Sarmiento,[1,11] Jordan F. Safer,[2,4] Connor W. Coley,[5,6] Bernhard Y. Renard,[1,7] Henrike O. Heyne,[1,7,8,9,10,12,*] and Sumaiya Iqbal[2,10,12,13,*]
[1]Hasso Plattner Institute, Digital Engineering Faculty, University of Potsdam, Potsdam, Germany
[2]Bioinformatics and Machine Learning, Broad Institute of MIT and Harvard, Cambridge, MA, USA
[3]Department of Software Engineering, Charles University, Prague, Czech Republic
[4]Faculty of Biology, Technion-Israel Institute of Technology, Haifa, Israel
[5]Department of Chemical Engineering, Massachusetts Institute of Technology, Cambridge, MA, USA
[6]Department of Electrical Engineering, Massachusetts Institute of Technology, Cambridge, MA, USA
[7]Hasso Plattner Institute for Digital Health at Mount Sinai, Icahn School of Medicine, New York, NY, USA
[8]Finnish Institute for Molecular Medicine (FIMM), University of Helsinki, Helsinki, Finland
[9]Program in Medical and Population Genetics, Broad Institute of MIT and Harvard, Cambridge, MA, USA
[10]Cancer Data Science, Dana-Farber/Harvard Cancer Center, Cambridge, MA, USA
[11]These authors contributed equally
[12]Senior author
[13]Lead contact
*Correspondence: francesca.rissom@hpi.de (P.F.R.), henrike.heyne@hpi.de (H.O.H.), sumaiya@broadinstitute.org (S.I.)
https://doi.org/10.1016/j.patter.2026.101635

THE BIGGER PICTURE Foundation models are large AI models that learn from the structure and patterns of large datasets without being trained for a specific task. They are increasingly trained on biological molecules and processes and can then be used to solve biological problems, such as predicting protein function or advancing drug discovery. This works because these models convert complex data into lists of numbers, called embeddings. Each embedding represents a protein, gene, or cell as a point in a high-dimensional space. Much of what a model learns is captured in the geometry of this embedding space: which samples lie close together, which lie far apart, and how biological categories are arranged. To assess what an embedding space has captured, or which one is best suited to a particular question, researchers today typically train additional predictive models using the foundation model's embeddings. This gives an informative answer for one specific task. However, it becomes computationally costly to scale across many tasks and embedding spaces and reveals little about how the embedding space is organized overall.

EmmaEmb offers a complementary approach. Instead of evaluating one task at a time, it examines how biological samples are arranged within a foundation model's embedding space as a whole. Using information researchers already have, such as functional annotations, taxonomic labels, or clinical metadata, it measures how different biological categories are represented and enables comparison between models. It also flags technical artifacts (so-called geometric distortions), that can mislead such comparisons. Although demonstrated here using protein models, we hope the EmmaEmb framework presented in this study will find use across other biological data, including gene expression, multi-omics, or cell imaging. As foundation models spread across biology, tools that help researchers understand and compare what they have learned will be increasingly valuable before investing in more resource-intensive training and evaluation.

SUMMARY

Embeddings, numerical vectors learned by deep-learning models, are increasingly used to represent complex molecular biology data and support predictive tasks and generative design. There is a growing need for systematic approaches to interpret and explain the information encoded in high-dimensional embedding spaces. Here, we introduce EmmaEmb, a quantitative, model-agnostic framework for geometric correction, direct analysis, and comparison of embedding spaces. Our framework encompasses local and global analysis methods to quantify data distribution within an embedding space and enable comparisons of representations across spaces in relation to known biological features. Through

This is an open access article under the CC BY license (http://creativecommons.org/licenses/by/4.0/).

experiments with seven embedding models across six molecular biology tasks, we demonstrate that our methods reveal insights from embedding spaces that align with downstream predictive tasks, uncover misclassification patterns, and contextualize differences in biological information captured by ProtT5, AlphaFold2, and ESM C. We provide an open-source Python library implementing all analysis methods and a guided diagnostic workflow.

INTRODUCTION

With the growing availability of data and computing power, foundation models have become increasingly relevant in molecular biology for encoding high-dimensional, multi-modal data. These models are trained to capture inherent relationships within large datasets, uncovering complex patterns and placing individual data points into a broader biological context. Protein language models (PLMs), for example, are a type of foundation model based on large language model architectures, trained on extensive protein sequence data from diverse species.[1–4] PLMs capture rich, biologically relevant, contextual information about protein structure, function, and evolution. Reportedly, PLMs are transforming many molecular and protein biology tasks, from structure and function prediction[3–7] to protein design and engineering.[8,9]

Challenges exist, however, in interpreting the information learned by foundation models due to their "black-box" nature, hidden biases, and dataset-specific artifacts.[10] Moreover, the proliferation of diverse architectures (e.g., layers and parameters) and training regimes (e.g., data modalities and training objectives) of foundation models has made it challenging to systematically assess which models are most suitable for specific biological questions. Such explainability and interpretability of foundation models are especially crucial in high-stakes applications in molecular biology and drug discovery.[11–13] As foundation models become tools for biological discovery, there is a pressing need for methods that can elucidate information captured by these models and guide their applications in a principled manner.

Foundation models encode the learned information from input data in numerical vectors, known as embeddings (i.e., latent spaces), and are therefore also referred to as embedding models. It has been demonstrated that embeddings are powerful in downstream prediction tasks[14–16] and as representational spaces for generative design.[9,17,18] However, because these vectors are high-dimensional, the encoded information is difficult to interpret directly. Probing embedding spaces offers a window into model learning, shedding light on how the model internally represents biological data. Current strategies for comparing embedding spaces of multiple models usually leverage machine learning (ML), such as training task-specific ML classifiers using embeddings to evaluate their predictive power[6] and correlating the embeddings with biological features to identify where specific feature information is encoded.[19,20] While effective, these approaches require additional training and are resource intensive.

A complementary method is to analyze the embedding spaces directly: in-space feature distribution and cross-space comparison. In natural language processing (NLP), the direct analysis of embeddings has revealed semantic relationships and biases within the data,[21,22] and comparison of embedding spaces has enabled knowledge transfer from high-resource to low-resource languages.[23] In molecular biology, the analysis of single embedding spaces has reportedly enhanced the understanding of continuous prediction tasks,[24,25] multi-modal data integration,[26] informed model uncertainty,[27] and improved interpretability by visualizing embeddings.[28] However, comparative analysis across embedding spaces remains underexplored in molecular biology. Although tools for embedding-space comparison exist (Table 1), their application to molecular biology remains limited, as most tools rely on visual inspection, providing few quantitative measures. Moreover, existing tools focus on individual data points, which are rarely meaningful in isolation for genes or proteins, where biological insight often emerges from patterns across functional groups.

To bridge this gap, we present a quantitative, model-agnostic framework, EmmaEmb (Embedding, Metadata, and Multimodel Analyzer for Exploration in Molecular Biology), for direct analysis and comparison of embedding spaces in molecular biology. We propose methods to analyze the distribution of data (i.e., biological features or metadata) within an embedding space and compare multiple embedding spaces using local neighborhood analyses and global distance metrics. We illustrate the utility of EmmaEmb through a series of experiments on PLMs, including embedding-space ranking for downstream tasks, interpreting misclassification patterns, and providing insights into how different model architectures encode data. Furthermore, we implemented a suite of diagnostic tools in EmmaEmb to uncover and adjust for geometric properties of embedding spaces (e.g., anisotropy and hubness) and to assess the robustness of conclusions with regard to data biases such as feature noise and class label imbalance. While we focus on molecular biology applications in this paper, our framework is generalizable to other data modalities. We publish a Python library that implements all components of EmmaEmb for open accessibility and reproducibility (https://github.com/broadinstitute/EmmaEmb).

In the next section, we detail the EmmaEmb framework and its analysis methods. We also describe the datasets and embeddings used in the subsequent results section to benchmark EmmaEmb in different applications. We end with the discussion, where we provide a summary along with further insights from the experiments, the limitations of EmmaEmb, and future directions.

METHODS

This section describes the EmmaEmb framework and its analysis methods, metrics, and visualization functions. Additionally, the datasets and embeddings utilized to validate EmmaEmb and its Python implementation are detailed.

Table 1. Comparison of published tools for analysis of multiple embedding spaces

Tool name	Dimensionality reduction techniques for visualization[a]	Feature distribution: visualization	Feature distribution: quantification	Feature distribution: neighbors	Space comparison: visualization	Space comparison: global analysis	Space comparison: neighborhood	Space comparison: feature integration	Code available	Tool description
EmmaEmb	PCA, t-SNE, UMAP	✔	✔	✔	✔	✔	✔	✔	✔	the framework and library introduced in this paper
repcomp[29]	–	–	–	–	–	✔	✔	–	✔	computation of similarities between two embedding spaces; nearest neighbors, canonical correlation, unit match; no visualization of results
embComp[30]	IsoMap, LLE, MDS, PCA, t-SNE, UMAP	–	–	–	✔	–	✔	–	–	visual local neighborhood analysis, based on KNN; distribution of similarities; cosine or Euclidean distance
Embedding comparator[31]	PCA, t-SNE, UMAP	–	–	–	✔	–	✔	–	✔	visual local neighborhood analysis, based on KNN; distribution of similarities; highlighting least similar samples and their nearest neighbors; cosine or Euclidean distance
Vectory[32]	PCA, UMAP	–	–	–	✔	✔	–	–	✔	aggregated similarity of local neighborhoods based on KNN; cosine or Euclidean distance
Emblaze[33]	PCA, t-SNE, UMAP	✔	–	–	✔	–	✔	–	✔	visual local neighborhood analysis, based on KNN; visualizing trajectories of points between embedding spaces; highlighting nearest neighbors of individual embeddings and user-selected sets of embeddings; cosine or Euclidean distance
Emb Compare[34]	PCA	✔	–	–	✔	✔	✔	–	✔	visual local neighborhood analysis, based on KNN; distribution of similarities; highlighting least similar samples; cosine distance

(*Continued on next page*)

Table 1. *Continued*

Tool name	Dimensionality reduction techniques for visualization[a]	Feature distribution: visualization	Feature distribution: quantification	Feature distribution: neighbors	Space comparison: visualization	Space comparison: global analysis	Space comparison: neighborhood	Space comparison: feature integration	Code available	Tool description
Latentverse[35]	PCA	✓	✓	–	✓	–	–	✓	✓	evaluation of latent representations, by assessing clustering, disentanglement, generalization, expressiveness, and robustness

[a]PCA, principal-component analysis; t-SNE, t-stochastic neighbor embedding; UMAP, uniform manifold approximation and projection; IsoMap (Tenenbaum et al.[36]); LLE, local linear embedding (Roweis and Saul[37]); MDS, multi-dimensional scaling (Kruskal[38]).

EmmaEmb framework

Figure 1 provides an overview of EmmaEmb and the proposed workflow. We begin by generating embeddings of data samples (Figure 1A), denoted as $s_1,\ldots,s_{N_S}$, where N_S is the total number of samples (e.g., proteins or genes). Embeddings of all samples from an embedding model are referred to as an embedding space, denoted by V. The hth model takes each sample s_i and maps it onto its corresponding embedding space $V_h \in \mathbb{R}^{d_h}$, producing the vector representation of embedding, $v_i^{(h)}$, where d_h is the dimensionality of the embedding vectors for model h, and N_V is the total number of embedding spaces compared.

$$s_i \longmapsto v_i^{(h)} \in V_h, \forall\, i = 1,\ldots,N_S, h = 1,\ldots,N_V, N_V \geq 2\ . \qquad \text{(Equation 1)}$$

Next, we integrate features (Figure 1B) of samples into EmmaEmb to analyze the information captured in the embedding spaces. The feature vectors can include biological groupings as metadata, such as protein classes, experimental annotations, or functional labels. Each feature vector of length N_S is denoted by f_p, where $p = 1,\ldots,N_f$, and N_f is the total number of feature vectors. We denote the value of the pth feature for sample s_i as $f_p[i]$.

Finally, EmmaEmb includes two complementary analysis approaches. The first approach, "feature distribution across spaces," examines how specific biological features are represented within and across embedding spaces (Figure 1C). The second approach, "pairwise space comparison," quantifies differences between embedding spaces directly (Figure 1D). The methods are modular and can be applied independently or in combination, depending on the task.

In both analysis approaches, we compare pairwise distances between samples across embedding spaces. For example, the distance between embeddings of samples s_i and s_j in space V_h is $\mathrm{dist}\left(v_i^{(h)}, v_j^{(h)}\right), h = 1,\ldots,N_V$. Because pairwise distances between samples are intrinsic properties of the embedding space that do not depend on dimensionality or axis alignment, they provide a coordinate-free basis for comparing how different models represent the same set of samples. These analyses assume that distances between embedding spaces are meaningful, that is, that the embedding space does not exhibit strong geometric biases such as anisotropy or hubness that can distort distance-based comparisons. Guidance on verifying and correcting for these properties is provided in the guided diagnostic workflow (see "EmmaEmb Python library" and Note S1). EmmaEmb considers multiple distance metrics, such as Euclidean, cosine, or Manhattan distances, capturing complementary aspects of proximity in high-dimensional spaces.[39]

The proposed analysis approaches can be performed on the local and global structures of samples in an embedding space. The local structure is the geometric relationship between each sample and its immediate neighbors in the embedding space. In contrast, the global structure represents the overall sample distribution and organization within the full space. For local structure analysis, we consider a subset of pairwise distances between each sample and its k-nearest neighbors (KNNs) within an embedding space. For a sample s_i in embedding space

CellPress
OPEN ACCESS

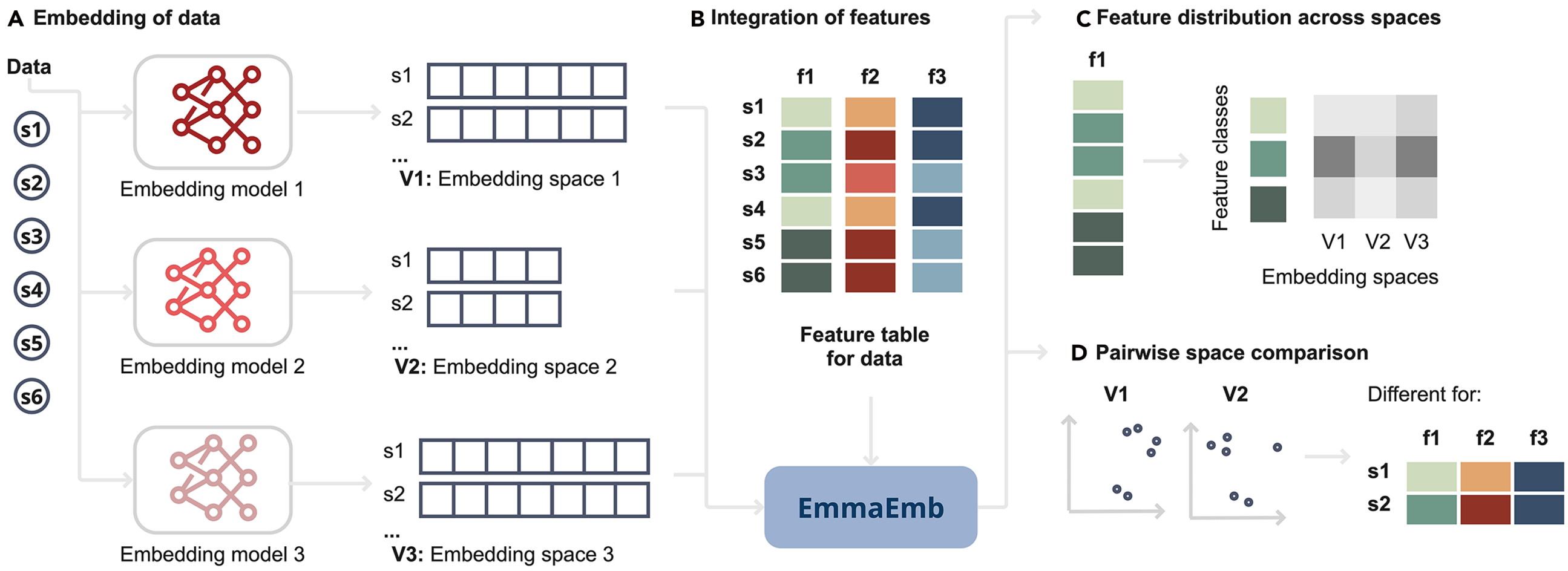

Figure 1. Schematic of the EmmaEmb framework and proposed workflow

(A) Starting with a set of data samples (e.g., genes or proteins, here s_1–s_6), embeddings are derived from different embedding models (here, embedding models V_1–V_3). For protein biology applications, embeddings can be derived from protein language models that vary in their architecture or training.

(B) Categorical features (here, f_1–f_3) associated with the samples are integrated into the analysis.

(C) Analysis approach 1. Feature distribution across spaces focuses on examining how samples with specific features, such as protein families, are grouped within the neighborhoods within an embedding space as well as across spaces. Multiple k-nearest-neighbor (KNN)-based methods can be applied within this approach to quantify the proximity of samples with the same feature as well as the proximity of groups of samples with the same feature.

(D) Analysis approach 2. Pairwise space comparison involves the identification of similarities and differences in distances between embeddings or KNNs between two embedding spaces. Regions of notable difference can be further analyzed using the features to characterize samples that are represented differently across the models.

Methods in (C) and (D) are modular and can be applied in any order, either independently within each approach or in combination between the approaches, depending on the analysis goals.

V_h, this neighborhood is denoted as $\mathcal{N}_{s_i}^{(h)}$. Following prior works,[27,40] we adapt KNN-based metrics and introduce several scores based on embedding neighborhoods within the EmmaEmb framework (Table 2; detailed below). We suggest analyzing embeddings and datasets using multiple distance metrics and a range of k values to capture local structures robustly and at different scales.

EmmaEmb analysis approach, 1: Feature distribution across spaces

This analysis approach enables the quantitative evaluation of how a selected feature (e.g., the functional class for a protein or gene; Figure 2A) is distributed within the neighborhoods of an embedding space. We propose the following two KNN-based methods and scores for quantitative assessments (Table 2).

KNN feature alignment score. The KNN feature alignment score quantifies how closely related a sample is to its embedding neighbors with respect to the feature f_p.

For sample s_i, the KNN feature alignment score in embedding space V_h is the proportion of its neighbors $\mathcal{N}_{s_i}^{(h)}$ with the same value of f_p:

$$\mathrm{Align}_p^{(h)}(s_i) := \frac{1}{k}\left|\left\{s_j \in \mathcal{N}_{s_i}^{(h)} : f_p[j] = f_p[i]\right\}\right|. \quad \text{(Equation 2)}$$

The score ranges from 0 to 1, with higher values indicating a greater representation of the feature class in the sample's neighborhood.

KNN feature alignment scores can be grouped by feature labels to examine how each feature class is captured in an embedding space. Furthermore, KNN feature alignment scores of one embedding space can be directly compared to those from another embedding space, enabling an assessment of how well different models capture relationships for the same feature.

KNN class matrix. To gain a broader view on how different feature classes relate to one another in an embedding space, we analyze the distribution of neighbors across classes. Specifically, for each embedding space V_h, we define a "KNN class mixing matrix" that captures how frequent samples from one class have neighbors from another class.

Let $\mathcal{C}_p = \{c_1, \ldots, c_{|\mathcal{C}_p|}\}$ denote the set of $|\mathcal{C}_p|$ unique categorical values (classes) of a feature f_p. For each class $c_a \in \mathcal{C}_p, \mathcal{S}_{c_a} = \{s_i : f_p[i] = c_a\}$ is the set of all samples belonging to class c_a. For a specific embedding space V_h and each pair of classes $(c_a, c_b) \in \mathcal{C}_p \times \mathcal{C}_p$, we count the number of neighbors of samples in class c_a that belong to class c_b to compute the KNN class mixing matrix, $M^{(h,p)} \in \mathbb{N}^{|\mathcal{C}_p| \times |\mathcal{C}_p|}$.

$$M_{a,b}^{(h,p)} := \sum_{s_i \in \mathcal{S}_{c_a}} \left|\left\{s_j \in \mathcal{N}_{s_i}^{(h)} : f_p[j] = c_b\right\}\right|. \quad \text{(Equation 3)}$$

A value of 0 indicates that none of the k-nearest neighbors of any sample in class c_a belong to class c_b, indicating that these two classes are separated within the embedding space according to the target feature. In contrast, the maximum value of $k \cdot N_{c_a}$ corresponds to when all neighbors of all samples in class c_a belong exclusively to class c_b. The KNN class mixing matrix, therefore, captures the extent of neighborhood proximity between classes. However, high neighborhood proximity between two classes does not necessarily imply a single large cluster; it could indicate the presence of multiple distinct subclusters within or between those classes.

Table 2. Overview of EmmaEmb's neighborhood-based formulas

Formula name	Formula	Description
KNN feature alignment score	$\text{Align}_p^{(h)}(s_i) := \frac{1}{k}\left\|\left\{s_j \in \mathcal{N}_{s_i}^{(h)} : f_p[j] = f_p[i]\right\}\right\|$	measures, for sample s_i, how well a feature is preserved within its local neighborhood $\mathcal{N}_{s_i}^{(h)}$ in embedding space h. Here, f denotes a feature vector over all samples, $f_p[i]$ the value of the pth feature for sample s_i, and s_j ranges over all k-nearest neighbors of s_i in $\mathcal{N}_{s_i}^{(h)}$. The score lies in [0,1], with 1 indicating that all k neighbors share the same feature value as s_i
KNN class mixing matrix	$M_{a,b}^{(h,p)} := \sum_{s_i \in S_{c_a}} \left\|\left\{s_j \in \mathcal{N}_{s_i}^{(h)} : f_p[j] = c_b\right\}\right\|$	summarizes how samples from class c_a are locally connected to samples from class c_b in embedding space h. For each sample s_i with class label $f_p[i] = c_a$, the formula counts neighbors $s_j \in \mathcal{N}_{s_i}^{(h)}$ whose feature value $f_p[j]$ equals c_b. The full matrix $M^{(h,p)}$ captures cross-class neighborhood patterns, providing a structured overview of how biological classes relate to one another in embedding space h
KNN class ranking matrix	$R_{a,b}^{(h,p)} := \text{rank}\left(M_{a,\cdot}^{(h,p)}\right)_b$	derived from the KNN class mixing matrix $M^{(h,p)}$. For each query class c_a, neighbor classes c_b are ranked according to their frequency in $M_{a,b}^{(h,p)}$, with rank 1 assigned to the most frequent neighbor class. The entries of $R^{(h,p)}$ range from 1 to $\|\mathcal{C}_p\|$, providing an order-based view of class mixing patterns in embedding space h
Cross-space neighborhood similarity	$\text{Sim}^{(h,h')}(s_i) := \frac{\left\|\mathcal{N}_{s_i}^{(h)} \cap \mathcal{N}_{s_i}^{(h')}\right\|}{k}$	quantifies how similar the local neighborhoods of sample s_i are across embedding spaces h and h'. The measure counts how many of the k nearest neighbors of s_i appear in both $\mathcal{N}_{s_i}^{(h)}$ and $\mathcal{N}_{s_i}^{(h')}$. The score lies in [0,1], where 1 indicates identical neighborhoods and 0 indicates no shared neighbors

To obtain a simplified summary of the proximity relationships, we further define a KNN class ranking matrix, $R^{(h,p)} \in \mathbb{N}^{|\mathcal{C}_p| \times |\mathcal{C}_p|}$, derived from $M^{(h,p)}$. For each query class c_a, the ath row of $R^{(h,p)}$ contains the rank indices of the neighbor classes sorted in descending order of $M^{(h,p)}$. In other words, $R_{a,b}^{(h,p)} = r$ means that class c_b is the rth most frequent neighbor class for query class c_a. The entries of $R^{(h,p)}$ therefore range from 1 to $|\mathcal{C}_p|$, with a value of 1 corresponding to the most frequent neighbor class and $|\mathcal{C}_p|$ to the least frequent.

EmmaEmb analysis approach, 2: Pairwise space comparison

Here, we compare two embedding spaces directly by analyzing differences in distances between samples or neighborhood structures and use feature information to interpret divergent regions (Figure 2B).

Global comparison of pairwise distances. To perform a global comparison of pairwise distances across embeddings, we compute Spearman's rank correlation coefficient (ρ) of pairwise distance values. This statistical measure allows us to evaluate how well the pairwise relationships between embeddings are preserved across different embedding spaces by comparing their ranking across different spaces. The pairwise distances in the two embedding spaces can be visualized in a scatterplot.

Cross-space neighborhood similarity. To quantify and analyze local differences between two embedding spaces V_h and $V_{h'}$, we first identify the k-nearest-neighbor sets $\mathcal{N}_{s_i}^{(h)}$ and $\mathcal{N}_{s_i}^{(h')}$ previously defined for each sample s_i. Following prior works,[30,31,33,34] we define the neighborhood similarity score for sample s_i as the fraction of overlapping neighbors between the two embedding spaces:

$$\text{Sim}^{(h,h')}(s_i) := \frac{\left|\mathcal{N}_{s_i}^{(h)} \cap \mathcal{N}_{s_i}^{(h')}\right|}{k}. \qquad \text{(Equation 4)}$$

This score ranges from 0 (no shared neighbors) to 1 (identical neighborhoods), capturing how well local neighborhood structures align across embedding spaces.

In addition to quantifying neighborhood overlap, EmmaEmb includes methods to analyze how the shared (i.e., overlapping)

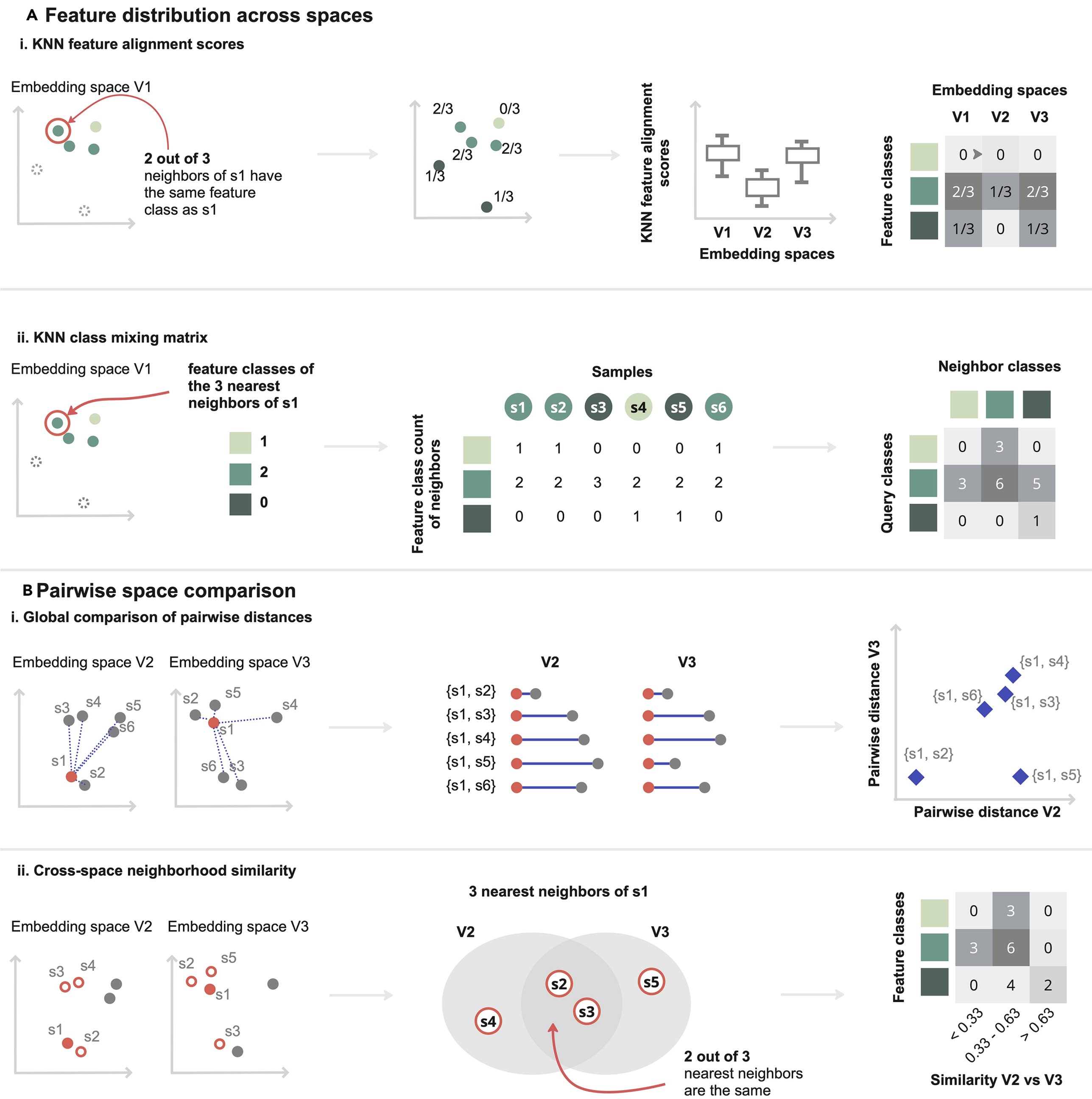

Figure 2. Graphical illustration of embedding-space analysis and comparison methods in EmmaEmb

(A) For the analysis of the feature distribution of a specific feature (here, feature f_1) for a set of samples (here, s_1–s_6) across embedding spaces (here, features V_1–V_3), we propose two methods. (i) KNN feature alignment scores. The KNN feature alignment scores are calculated for each embedding within its embedding space, as a fraction of its *k*-nearest-neighboring embeddings that share the same class label as the embedding itself. By comparing the distribution of KNN feature alignment scores across different embedding spaces, we can assess how closely embeddings of the same f_1 class are to other embeddings of the same class. The score provides insights into how well an embedding model has learned to group embeddings with similar characteristics. (ii) KNN class mixing matrix. The matrix is calculated separately for each embedding space (here, for V_1). For each embedding, the class distribution of class labels of its *k*-nearest neighbors is computed. The class distributions are then summed for all embeddings of the same class. The resulting matrix shows, for each class, the number of *k*-nearest neighbors of embeddings from this class for another class. This allows insights into which classes are close to each other, and which are more differentiated within the embedding space.

(B) We also propose methods for pairwise embedding-space comparison. (i) Global comparison of pairwise distances (here, between embedding spaces V_2 and V_3). For this method, we first calculate all pairwise distances between embeddings within each embedding space. We then compare the correlation of pairwise distances, e.g., distance between $\{s_1, s_2\}$, across the two embedding spaces. This method provides an overview of the global similarity between embedding spaces and reveals groups of samples that are differently represented. (ii) Cross-space neighborhood similarity, in contrast, quantifies local differences between two embedding spaces. It is calculated for each sample as the number of *k*-nearest neighbors shared between V_2 and V_3. Identifying and characterizing samples with high divergence in their local neighborhood can help to uncover structural differences between the embedding spaces.

neighbors are distributed across classes in relation to a categorical feature f_p, offering insights into whether neighborhood similarity reflects meaningful biological groupings.

EmmaEmb Python library

We implemented EmmaEmb as a lightweight, modular Python library,[41] centered around the Emma object that handles embedding spaces, feature data, and intermediate computational results. Multiple functions on the Emma object enable analysis of feature distributions across embedding spaces, including feature alignment and neighborhood analysis (Figure 2A), as well as pairwise space comparisons between different embedding spaces (Figure 2B). EmmaEmb integrates functionalities from established libraries such as scikit-learn[42] and Plotly[43] to calculate the scores (Table 2) and offer intuitive visualizing functions. For distance measurement, EmmaEmb uses a variety of metrics, including Manhattan, Euclidean, and cosine distances, leveraging standard implementations from scikit-learn.[42] To ensure scalability for very large datasets, we provide the option to approximate neighborhood searches using the ANNOY algorithm,[44] which allows efficient identification of approximate nearest neighbors in high-dimensional spaces.

Geometric diagnostics and mean centering

EmmaEmb's local and global methods assume that distances between embeddings are meaningful. This assumption can be violated when embedding spaces exhibit anisotropy or hubness. Anisotropy refers to the tendency of embedding vectors to cluster in a narrow directional cone rather than spreading uniformly, which inflates cosine similarity between all pairs and reduces the discriminative power of distance metrics.[45] Hubness refers to the phenomenon whereby a small number of points become the nearest neighbors of a disproportionately large fraction of other points—a mathematical consequence of high dimensionality that makes KNN-based scores unreliable.[46] To allow users to empirically verify these assumptions before proceeding, EmmaEmb includes geometric diagnostic functions covering anisotropy (average cosine similarity and per-dimension variance, following Ethayarajh[45]) and hubness (k-occurrence distribution skewness and Robin Hood index [RHI], following Radovanovic et al.[46] and Feldbauer et al.[47]). Where anisotropy is detected, EmmaEmb provides a function to apply mean centering, subtracting the mean embedding vector from all embeddings, as a correction prior to all subsequent analysis steps.

Metadata requirements and feature integration

EmmaEmb is primarily designed for categorical metadata, although continuous features can be incorporated via binning or used directly for visual exploration after dimensionality reduction. Neighborhood-based scores require at least k samples per group to be interpretable, and EmmaEmb automatically flags when this constraint is violated. Further, EmmaEmb provides missing data flagging when loading a feature dataframe, with columns exceeding a configurable threshold of missing values flagged before analysis proceeds.

Guided diagnostic workflow for embedding-space ranking

To support systematic and reproducible analysis of how well different embedding spaces encode task-relevant biological features, EmmaEmb provides a six-step guided diagnostic workflow (see Note S1 for details) that structures the analysis from geometric verification through to robustness analysis. The geometric diagnostics in steps 1 and 2 characterize properties of the embedding space itself and are recommended prior to any EmmaEmb analysis, including pairwise space comparison. Steps 3 through 6 are specific to feature distribution analysis and the ranking of embedding spaces by their alignment with a given biological feature and are run separately for each feature of interest.

Step 1. Anisotropy assessment: computes average cosine similarity on raw and mean-centered embeddings and per-dimension variance to detect and correct for geometric directional bias; determines the recommended distance metric and whether mean centering should be applied

Step 2. Hubness assessment: computes the k-occurrence distribution and RHI on both raw and mean-centered embeddings (evaluated at neighborhood sizes k = 5, 10, 20); the severity of hubness informs the ceiling on the neighborhood size k used in the analysis steps 3–6

Step 3. Class distribution and neighborhood size range: determines the appropriate range of neighborhood sizes (k) for analysis in steps 4–6, based on class sizes, dataset size, and hubness severity from step 2; computes the expected random baseline alignment score per class; flags missing data and class imbalance

Step 4. Parameter sensitivity analysis: evaluates ranking stability of mean KNN feature alignment scores across the determined k range and selected distance metrics, producing a ranking stability plot

Step 5. Class imbalance robustness (run if flagged in step 3): progressively downsamples the majority class and recomputes mean KNN feature alignment scores at each subsampling ratio, assessing whether embedding-space rankings are stable across different class balance conditions

Step 6. Feature noise robustness: progressively permutes a fraction of annotations while keeping embeddings fixed, recomputing mean KNN feature alignment scores at each noise level to assess whether embedding-space rankings are robust to annotation uncertainty

Based on the diagnostic results, the workflow provides guided recommendations for distance metric selection and k range, ensuring that parameter choices are appropriate for the embedding spaces and datasets analyzed. A step-by-step walkthrough example can be accessed as an interactive Google Colab notebook in the EmmaEmb repository.

Library design and extensibility

The EmmaEmb library is designed as a modular framework that can be extended for further types of analysis, such as clustering approaches or topological analysis. Additionally, the repository includes a script that facilitates the extraction of embeddings from five PLMs listed in Table 3, including sequence chopping and embedding rejoining algorithms to process long sequences.

Experiments

We applied EmmaEmb to the following six molecular biology applications and datasets. A summary of each dataset, including the distributions of samples per class, is provided in

CellPress
OPEN ACCESS

Table 3. Overview of the protein language models used in this study

Model name	Reference	Version	No. of parameters	Embedding length	Training
Ankh	Elnaggar et al.[48]	large	1.15 billion	1,536	only trained on amino acid sequence data
ProtT5	Elnaggar et al.[2]	prot_t5_xl_uniref. 50	3 billion	1,024	only trained on amino acid sequence data
ProstT5	Heinzinger et al.[6]	–	3 billion	1,024	ProtT5 fine-tuned for translation between sequence and structure
ESM-2	Lin et al.[3]	esm2_t36_3B_UR50D[a]	3 billion	2,560	only trained on sequence data
ESM C	ESM Team[49]	esmc-300m-2024-12	300 million	960	related to the multi-modal model ESM3 (Hayes et al.[4]), but optimized for representation learning

[a]For ligand-binding sites (three-class), a different ESM-2 model version was used: esm2_t33_650M_UR50D. All other tasks used esm2_t36_3B_UR50D.

Tables S1–S6. We performed experiments with seven different embedding models: five PLMs[2–4,6,48] (Table 3), a representation-adapted fine-tuned embedding model (ProtTucker),[50] and a supervised deep-learning model (AlphaFold2).[1]

Datasets

Subcellular localization. We utilize the training set of version 1.0 of the DeepLoc dataset, which consists of 11,231 protein sequences from eukaryotes annotated with subcellular localization information across ten distinct classes.[51]

Residue conservation. We obtain residue conservation scores from the ConSurf10k datasets provided by Marquet et al.,[14] which classifies each residue into one of nine conservation categories ranging from 1 (highly variable) to 9 (highly conserved) based on ConSurf-DB.[52] Due to computational constraints, we excluded proteins with lengths >1,022 residues, resulting in a dataset of 2,398,737 residue annotations.

Residue binding (binary). The residue-binding dataset comprises 170,686 annotated residues from the *DevSet1014*,[16] including binding to small molecules, metal ions, and DNA/RNA (excluding protein-protein interactions). Each residue is labeled as either binding or non-binding.

Ligand-binding site (three-class). We constructed a new dataset[53] combining three sources for multi-class residue-level annotation of ligand-binding sites, distinguishing between regular binding, cryptic binding, and non-binding residues. The training set was taken from the CryptoBench train split (cryptic)[54,55] and sc-PDB enhanced (regular), an augmented version of sc-PDB in which additional pockets identified in alternative protein conformations were added using AHoJ-DB.[56–62] A test set was compiled from the LIGYSIS benchmark (regular),[63,64] a recently published dataset of ligand-binding human proteins, and the CryptoBench test split (cryptic). Data leakage was controlled separately for each source: the CryptoBench train/test split was used as provided, and scPDB-enhanced was filtered to a maximum of 10% pairwise sequence identity against the LIGYSIS test set using MMseqs2.[65] To address class imbalance, all three classes were randomly downsampled to match the smallest class in both the training and test sets, yielding a balanced training set of 38,898 residues (∼12,966 per class) across 1,082 proteins and a balanced test set of 9,336 residues (∼3,112 per class) across 282 proteins. Therefore, each residue was annotated as one of three classes: regular binding, cryptic binding, or non-binding.

Protein structure (CATH). We use the *test300* dataset from CATH version 4.3,[66] as defined by Heinzinger et al.[50] This set contains 300 proteins. For our analysis, we focus on the highest hierarchical level, class C, which groups proteins based on their secondary structure composition into five classes: mainly alpha, mainly beta, alpha beta, few secondary structures, and special.

Phospholipase A2 family. This dataset consists of proteins from the phospholipase A2 family, specifically the phospholipase A2 isozyme group II (PLA2G2).[67,68] The dataset includes protein sequences annotated across seven different species and categorized into 11 different enzyme classes. Only the proteins with both species and enzyme class information were included, resulting in a final set of 446 proteins for our analysis.

Embeddings

PLM embeddings. We validated EmmaEmb analysis methods using five transformer-based PLMs: Ankh,[48] ProtT5,[2] ProstT5,[6] ESM-2,[3] and ESM C[49] (Table 3). For residue-level prediction tasks, we use the embedding corresponding to the respective residue position. For all models, embeddings were extracted from the final hidden layer of each respective model. As some of these models have restrictions on the maximum sequence length, protein sequences longer than 1,022 amino acids were excluded from residue-level analyses, which affected 34 genes in the residue conservation task. For protein-level prediction tasks, we compute the mean of residue-level embeddings to obtain a single vector per protein. For sequences exceeding the maximum input length, protein sequences are divided into smaller chunks, and each chunk is processed with a minimum overlap of 300 amino acids to maintain sequence continuity. As proposed by Brandes et al.,[69] to ensure that the entire sequence is effectively represented, chunks were taken with overlapping windows from both sides, and, if necessary, an additional 1,022-amino-acid window was included in the middle to ensure full coverage of the input sequence. The embeddings for each chunk are then aggregated using the mean of all chunk embeddings, resulting in a single unified representation for the full-length protein. For convenience, this embedding pipeline, including chunking and aggregation, is implemented in the EmmaEmb library.

ProtTucker embeddings. We obtain ProtTucker embeddings using the code and pretrained models provided by the authors.[50] We follow their default settings without modifications to ensure consistency with the original work. For protein-level analyses, we compute the mean of the residue-level embeddings to obtain a single vector representation per protein.

AlphaFold embeddings. AlphaFold2 embeddings were generated using ColabFold[70] (v.1.5.2) with default parameters, including MMseqs2[65] for generation of multiple sequence alignments. Specifically, we extract the single representation, a residue-level embedding produced during the structure prediction process, and perform mean pooling across the residue embeddings to retrieve protein-level embeddings.

Geometric diagnostics were run for all embedding spaces and datasets used in this study. Where anisotropy was detected, mean centering was applied to the embedding space as a correction before all subsequent analysis steps including hubness computation, distance calculation, and neighborhood scoring. Diagnostic outputs and whether mean centering was applied are reported for each embedding space and dataset in Table S9.

Transfer model for ligand-binding site (three-class) prediction

For the ligand-binding-site task, we trained a shallow three-layer multi-layer perceptron (MLP) with 1,024 neurons for each layer.[53,71] The model was trained for 10 epochs. A dropout of 0.3 was utilized, together with AdamW optimizer and learning rate of 5×10^{-6}, batch size equal to 2,048, and focal loss with gamma of 7. A single model was trained for each embedding space. The hyperparameters were determined using a random 80/20 split optimizing for area under the precision-recall curve (AUPRC). An overview of model performance across a range of evaluation metrics (accuracy, area under the curve [AUC], Matthews correlation coefficient, F1 score, and AUPRC) is provided in Table S8.

Statistical tests

All statistical comparisons between KNN feature alignment score distributions of models were performed using one-sided Wilcoxon signed-rank tests, testing whether model 1 had greater values than model 2. Rank-biserial correlations are reported as effect-size measures. p values and rank-biserial correlations were computed with the Python package Pingouin.[72]

RESULTS

In this section, we report the results and insights of embedding analysis experiments conducted using EmmaEmb on a diverse set of molecular biology applications and seven different embedding models.

Embedding-space analysis using EmmaEmb ranks PLM representations by their relevance for downstream tasks

We investigated whether direct analysis of the embedding-space structure using EmmaEmb can serve as a proxy for evaluating the task-relevant information captured by different PLMs. Specifically, we assessed whether the local organization of embeddings of samples, measured through the distribution of task-specific labels within their neighborhoods, can be used to rank embedding spaces in a manner consistent with their performance on the downstream task. In this study, transfer learning refers to an approach in which representations learned by a large pretrained model are used as input features for a smaller task-specific model.

We evaluated four PLMs—Ankh,[48] ProtT5,[2] ProstT5,[6] and ESM-2[3]—across four benchmark classification tasks for proteins: subcellular localization (i.e., protein location within a cell),[51] residue conservation (evolutionary persistence of amino acids),[14,52] residue binding (binary annotation of amino acids as binding or non-binding),[16] and ligand-binding site (three-class annotation of residues as non-binding, binding, or cryptic; where cryptic binding sites are absent or occluded in the ligand-free protein conformation but become accessible upon conformational change, making them of particular interest as drug targets). For the first three tasks, we benchmark the ranking of embedding spaces using EmmaEmb against published transfer learning performance from Heinzinger et al.,[6] including a light-attention model for subcellular localization and two-layer convolutional neural networks for residue conservation and binary residue binding, providing an independent comparison that does not rely on classifiers trained in this study. For the ligand-binding site (three-class) task, we trained a dedicated MLP classifier on a newly constructed dataset (see methods). This enables direct comparison of EmmaEmb rankings against transfer learning performance generated in this study. For each task, we computed the mean KNN feature alignment scores (Align, Equation 2) using EmmaEmb across multiple neighborhood sizes (k values) and distance metrics (cosine, Manhattan, and Euclidean). To handle the large-scale residue conservation and ligand-binding site datasets (~2.4 million embeddings), we used the ANNOY algorithm[44] with 100 trees to efficiently approximate KNNs, demonstrating the scalability of EmmaEmb to millions of samples. Table S7 summarizes the transfer model training and test datasets, model architectures, and performance metrics and measures applied to limit homology between training and test sets for the four tasks. Importantly, EmmaEmb KNN feature alignment scores were computed on training set embeddings and compared against transfer learning performance evaluated on held-out test sets, ensuring that the correspondence between the two measures reflects genuine generalization of embedding structure.

For subcellular localization, mean KNN feature alignment scores consistently ranked ProtT5 highest across all k values (range 1–100) and ProstT5 lowest, while the relative ranking of Ankh and ESM-2 varied slightly across k values (Figure 3A). This ranking pattern was consistent before and after mean centering and across distance metrics (Figure S1A). Statistical testing confirmed these trends: ProtT5 scores were significantly higher than those of Ankh and ESM-2 (one-sided Wilcoxon signed-rank test at $k = 100$: rank biserial correlations 0.41–0.50; all $p < 10^{-10}$), and both Ankh and ESM-2 significantly outperformed ProstT5 (rank biserial correlations 0.22–0.37; all $p < 10^{-10}$; Table S10). This embedding-based ranking using EmmaEmb reflects overall reported model performance on this task, with ProtT5 achieving the highest transfer accuracy (10-state per-protein accuracy = 64.9%), ahead of Ankh (63.7%), ESM-2 (62.9%), and ProstT5 (57.3%).[6]

CellPress
OPEN ACCESS

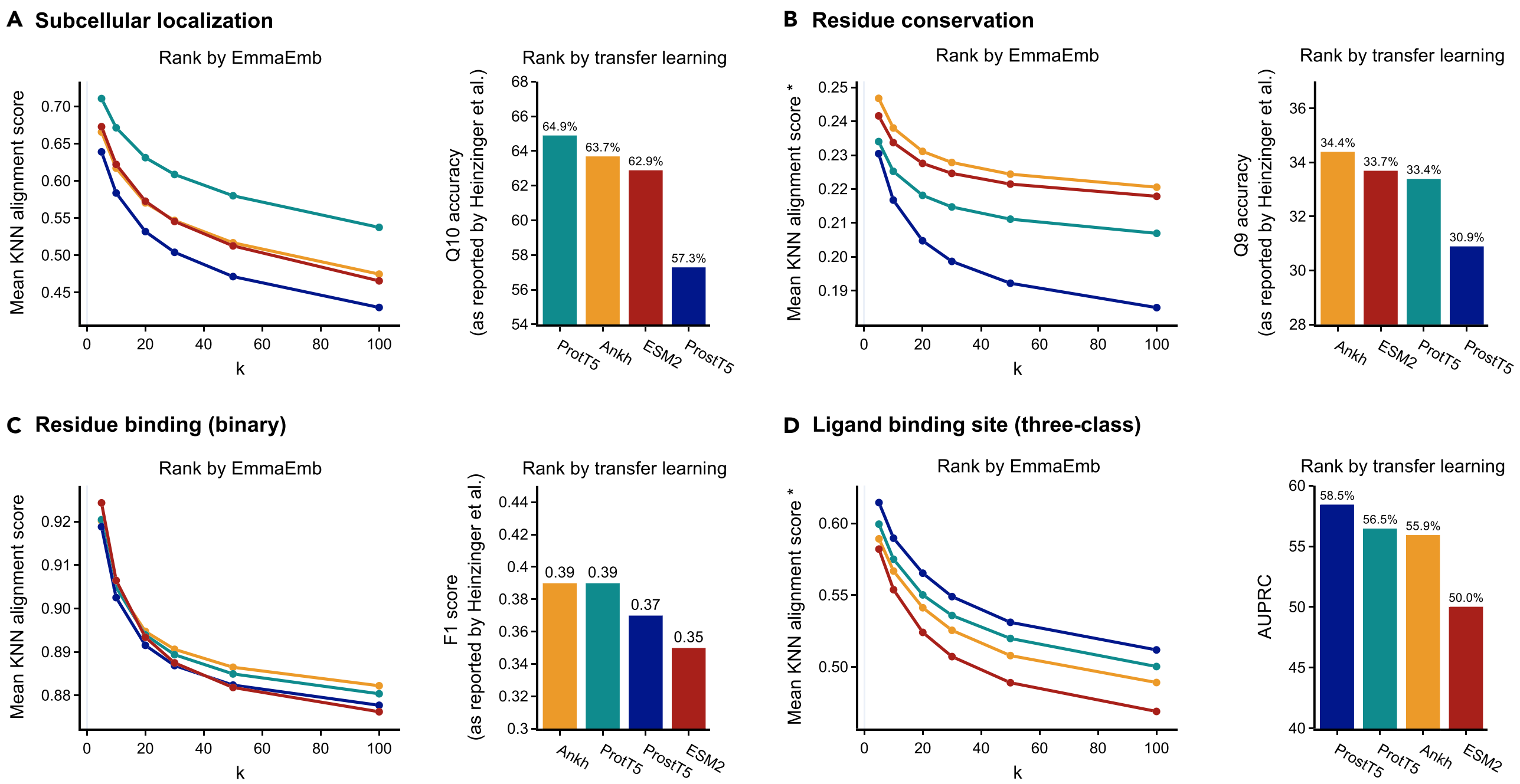

Figure 3. Representational relevance of protein language models assessed by embedding-space analysis vs. transfer learning

Mean KNN feature alignment scores (*y* axis, EmmaEmb, cosine distance) across varying *k* values (*x* axis) are shown along downstream transfer learning performance (bar charts) for the four PLMs Ankh,[48] ProtT5,[2] ProstT5,[6] and ESM-2[3] across four tasks. Embedding-space analysis was performed on training set embeddings; transfer learning performance was evaluated on held-out test sets.

(A) Protein subcellular localization (transfer learning performance: Q10, 10-state-per-protein accuracy, as reported by Heinzinger et al.[6]).

(B) Residue conservation (transfer learning performance: Q9, 9-state-per-residue accuracy, as reported by Heinzinger et al.[6]; KNN approximated using the ANNOY algorithm[44]).

(C) Residue binding, binary (transfer learning performance: F1 score, as reported by Heinzinger et al.[6]).

(D) Ligand-binding site, three-class (transfer learning performance: AUPRC, generated in this study using an MLP classifier trained on combined CryptoBench and scPDB-enhanced training sets and evaluated on the combined LIGYSIS and CryptoBench test sets; see methods).

For all four tasks, the ranking of embedding spaces by mean KNN feature alignment score is consistent with their ranking by downstream transfer learning performance, i.e., the line order at large *k* mirrors the bar-chart order. For tasks (B)–(D), this consistency is observed only after mean centering correction and with cosine distance; results prior to mean centering and across all three distance metrics (cosine, Manhattan, Euclidean) are provided in Figure S1.

For the residue conservation task (Figure 3B), geometric diagnostics revealed strong hubness for ESM-2 embeddings prior to correction (RHI = 0.958), which substantially resolved after mean centering (RHI = 0.494; Table S9). The geometric correction impacted the observed embedding-space rankings: prior to mean centering, the ranking of ESM-2 relative to other models was inconsistent with downstream transfer learning performance (Figure S1B). After mean centering, EmmaEmb ranked Ankh, ESM-2, ProtT5, and ProstT5 (high to low) across *k* values (1–100), consistent with the downstream transfer learning performance (9-state per-residue accuracy = 34.4%), followed by ESM-2 (33.7%), ProtT5 (33.4%), and ProstT5 (30.9%).[6] This demonstrates that correcting for geometric biases before analysis is not merely a technical formality but can directly affect conclusions about model prioritization. Statistical testing confirmed this ordering: Ankh scored significantly higher than ESM-2 (one-sided Wilcoxon signed-rank test at $k = 100$: rank biserial correlation 0.05, $p < 10^{-10}$), ESM-2 was higher than ProtT5 (rank biserial correlation 0.23, $p < 10^{-10}$), and ProtT5 outperformed ProstT5 (rank biserial correlation 0.20, $p < 10^{-10}$) (Table S11).

For binary residue binding (Figure 3C), geometric diagnostics again revealed strong hubness for ESM-2 embeddings prior to correction (RHI = 0.560), which was substantially reduced after mean centering (RHI = 0.268; Table S9). Prior to mean centering, embedding-space rankings fluctuated substantially across distance metrics (Figure S1C). Following correction, EmmaEmb consistently ranked Ankh above ProtT5 and ProtT5 above ProstT5 across *k* values. ESM-2 ranked highest at small *k* values but lowest at larger *k* values ($k = 50$–100), preventing a reliable ranking for this model. Statistical testing confirmed the ordering among the three stable models: Ankh scores were significantly higher than those of ProtT5, and ProtT5 scores were significantly higher than those of ProstT5 (one-sided Wilcoxon signed-rank test at $k = 100$: rank biserial correlations 0.06–0.09; all $p < 10^{-10}$; Table S12). The ranking of Ankh, ProtT5, and ProstT5 is broadly consistent with their downstream transfer learning performance (F1 score: Ankh = 0.39, ProtT5 = 0.39, ProstT5 = 0.37, and ESM-2 = 0.35).

A key challenge for this dataset is its severe class imbalance (8.2% binding vs. 91.8% non-binding), which may affect neighborhood-based metrics, as the proportion of same-class neighbors a binding residue can have is inherently constrained by the rarity of the class. To assess the robustness of embedding-space rankings under different balancing conditions, we

performed progressive class rebalancing by progressively downsampling the majority (Figure S2). Rankings for Ankh, ProtT5, and ProstT5 remained consistent across subsampling ratios, while ESM-2 showed unstable ranking relative to the other models, indicating that its ranking is sensitive to class distribution and should be interpreted with caution. We additionally performed progressive feature perturbation ablations, where an increasing fraction of annotations was randomly shuffled while embeddings were held fixed (Figure S3). Despite the strong class imbalance, all four models' mean KNN feature alignment scores declined progressively with increasing permutations, converging toward the expected random baseline.

For ligand-binding sites (three-class) (Figure 3D), geometric diagnostics revealed anisotropy resolved by mean centering, especially for ESM2 embeddings prior to correction (average cosine similarity 0.356 before and 0.003 after mean centering; Table S9). As observed for residue conservation, embedding-space rankings prior to mean centering were inconsistent with downstream transfer learning performance across distance metrics (Figure S1D). Following correction, EmmaEmb ranked ProstT5 highest, followed by ProtT5, Ankh, and ESM-2 across k values, consistent with their downstream transfer learning performance (AUPRC: ProstT5 = 58.5%, ProtT5 = 56.5%, Ankh = 55.9%, and ESM-2 = 50.0%). Statistical testing confirmed the EmmaEmb ordering (one-sided Wilcoxon signed-rank test at $k = 100$: rank biserial correlations 0.09–0.17; all $p < 10^{-10}$; Table S13).

Together, these results demonstrate that EmmaEmb can quantify how readily task-relevant information is encoded and accessible in an embedding space, offering a fast, training-free proxy for transfer learning performance and model prioritization, as shown here against both published transfer learning benchmarks and a classifier trained in this study. Correcting for geometric biases and evaluating rankings across k values, class balance, and feature noise conditions allows users to assess not just the ranking itself but the confidence with which it can be reported.

EmmaEmb analysis captures class-wise transfer learning performance and provides insights into misclassification patterns

Building on the model-level comparisons described above, we next examined whether embedding-space neighborhoods could capture class-specific transfer learning performance and misclassification tendencies. Per-class transfer learning performance was available for subcellular localization and ProtT5 from Stärk et al.[15] for residue conservation and ProtT5 from Marquet et al.,[14] as well as for the ligand-binding site (three-class) task from the MLP classifier trained in this study, enabling class-level analysis across all multi-class tasks examined and across both literature-reported and study-generated transfer learning benchmarks.

For all three tasks, we computed mean KNN feature alignment scores (Align, Equation 2, $k = 100$, cosine distance) for each class and compared them with per-class transfer learning recall. A strong Spearman correlation between per-class EmmaEmb alignment scores and per-class transfer learning recall was observed across all three tasks: $\rho = 0.97$ ($p < 0.001$) for subcellular localization, $\rho = 0.92$ ($p < 0.001$) for residue conservation, and $\rho = 1.00$ ($p < 0.001$) for ligand-binding site (three-class) (Figures 4A.1–4C.1). Notably, this also held for smaller class sizes; for example, in the subcellular localization task, embeddings for proteins in the plastid, despite their relatively low representation (605 proteins, approximately 5% of the overall dataset), showed the second highest prediction recall in transfer learning (0.92, Figure 4A.1), and also second highest mean KNN feature alignment scores (0.69, Figure 4A.1) in the embedding space.

To better understand the relationship between different feature classes in the embedding space, we calculated the KNN class ranking matrix (R, derived from M, Equation 3), which analyzes the proximity of different feature classes with other feature classes in the embedding space. When comparing these distributions with the predicted class distributions from the transfer learning models, we found a close structural similarity across all three tasks (Figures 4A.2–4C.2; full fractional distributions in Figure S4). For subcellular localization, the most frequently misclassified class is also the most common neighboring class in the embedding space for nine of ten classes. For residue conservation, this holds for six of nine classes, with the most frequently misclassified class appearing as the second most common neighbor for two further classes. For ligand-binding site (three-class), all classes show this pattern. For classes that are not yet well differentiated in the embedding space, such as "cytoplasm," our approach allows a deeper investigation into which neighboring feature classes they tend to mix with. For example, proteins labeled as "cytoplasm" were frequently embedded near proteins from the "nucleus" and "mitochondrion" classes, both of which are common sources of misclassification by the transfer learning model. The insights expose the structural roots of model errors and can guide more targeted approaches to model refinement and domain-specific fine-tuning.

EmmaEmb supports analysis for diverse embedding models and assessment of model learning

To examine how embedding-space analysis can be used to monitor representational changes during model adaptation, we compared embeddings from the original ProtT5 model with those from ProtTucker,[50] a model that adds task-specific layers to adapt the ProtT5 embedding space for CATH structural classification[66] using contrastive learning. We analyzed the embeddings of 300 proteins from the ProtTucker test set that were not seen during the ProtTucker training. Across a range of neighborhood sizes and distance metrics, ProtTucker embeddings consistently exhibited higher mean KNN feature alignment scores with respect to CATH class feature than ProtT5 (Figures 5A and S5A), reflecting improved capture of structural hierarchy information by ProtTucker embeddings.

We next compared ProtTucker and ProtT5 embeddings to those from AlphaFold2,[1] a model that also captures protein structural information. Interestingly, AlphaFold2 embeddings exhibited KNN feature alignment scores comparable to those of ProtT5, while scores in both AlphaFold2 and ProtT5 remained consistently below those of ProtTucker (Figure 5A). This trend was further confirmed by statistical testing, which showed the KNN feature alignment scores to be significantly higher for ProtTucker embeddings compared to ProtT5 and AlphaFold2

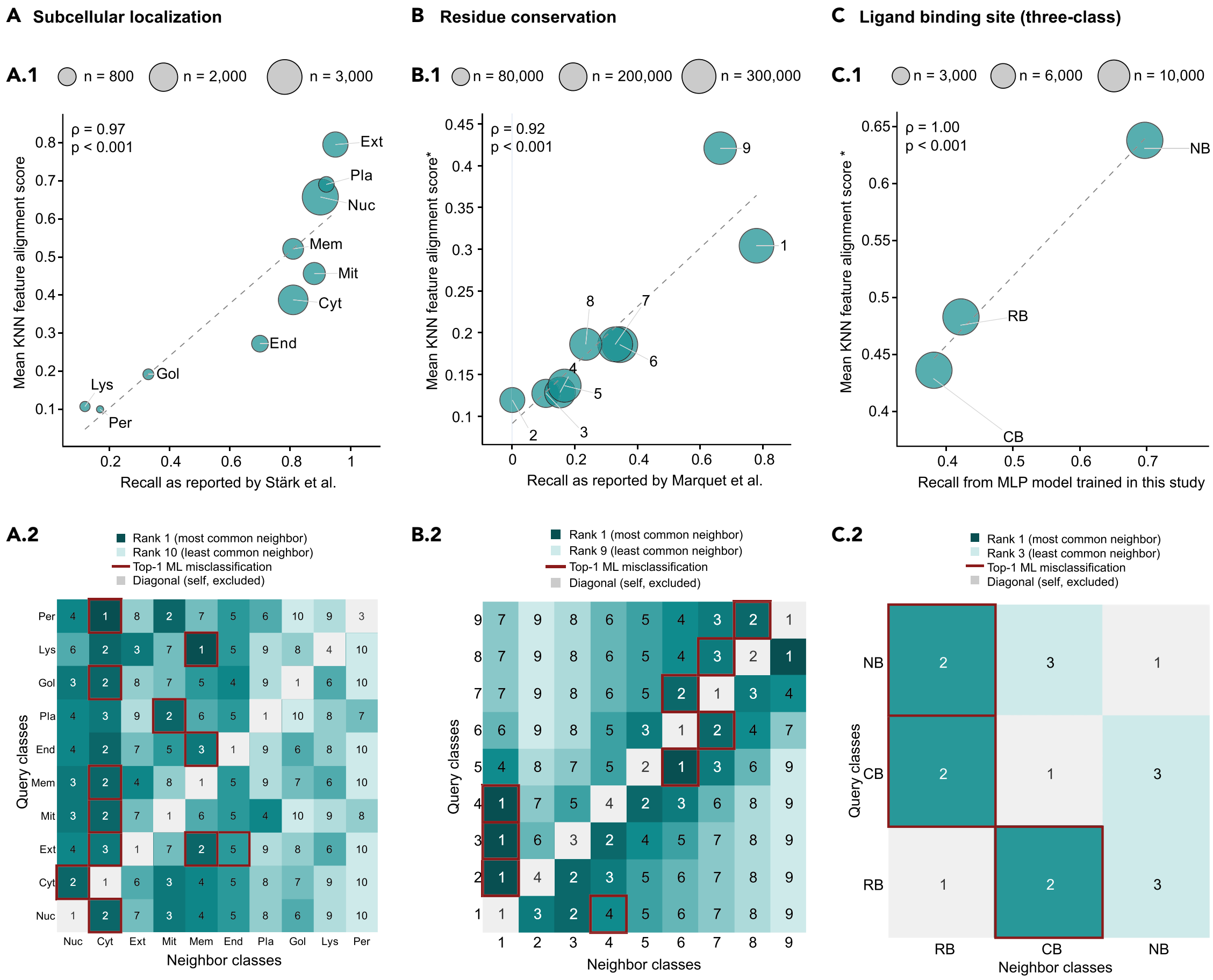

Figure 4. Class-level embedding-space analysis vs. transfer learning analysis across three tasks

Embedding-space analysis based on $k = 100$, cosine distance, performed on training set embeddings after mean centering. All panels (A.1, B.1, C.1) on the top row show scatterplots of mean KNN feature alignment scores (*y* axis, EmmaEmb) vs. per-class transfer learning recall (*x* axis), with dot size reflecting class sample count; asterisk on the *y*-axis label indicates that KNN neighborhoods were approximated using the ANNOY algorithm. All panels on the bottom row (A.2, B.2, C.2) show KNN class ranking matrices where, for each query class (*y* axis), numbers and colors indicate the ranked frequency of each neighbor class in the embedding space from most to least common. Red boxes indicate the top-1 misclassification as reported by the transfer learning model. Diagonal cells (self, excluded) are shown in gray. Fractional neighbor class distributions underlying each ranking matrix are provided in Figure S4.

(A) Subcellular localization. ProtT5 embedding space. Transfer learning performance as reported by Stärk et al.[15] An overall high correlation (Spearman's $\rho = 0.97$, $p < 0.001$) is observed, with some smaller classes (e.g., Pla, plastid) both well predicted and well represented in the embedding space. For nine out of ten classes, the most frequently misclassified class is also the most common neighboring class in the embedding space; for the extracellular (Ext) class, three classes share an equal misclassification pattern. Cyt, cytoplasm; End, endoplasmic reticulum; Ext, extracellular; Gol, Golgi apparatus; Lys, lysosome/vacuole; Mem, cell membrane; Mit, mitochondrion; Nuc, nucleus; Per, peroxisome; Pla, plastid.

(B) Residue conservation. ProtT5 embedding space. Transfer learning performance as reported by Marquet et al.[14] The correlation between per-class EmmaEmb alignment scores and per-class transfer learning recall is high ($\rho = 0.92$, $p < 0.001$). For six of nine classes, the most frequently misclassified class is also the most common neighboring class in the embedding space; for two further classes, the most frequently misclassified class is the second most common neighboring class. Conservation categories 1–9 range from highly variable to highly conserved.

(C) Ligand-binding site (three-class). ProstT5 embedding space, selected as the best-performing embedding space for this task. Transfer learning performance from the MLP classifier trained in this study. The correlation between per-class EmmaEmb alignment scores and per-class transfer learning recall is perfect ($\rho = 1.00$, $p < 0.001$). For all three classes, the most frequently misclassified class is also the most common neighboring class in the embedding space. The lower per-class recall for regular and cryptic binding residues (C.1) reflects the classifier's tendency to mix these two structurally similar classes, as shown in the confusion matrix (C.2). NB, non-binding; RB, regular binding; CB, cryptic binding.

embeddings (one-sided Wilcoxon signed-rank test at $k = 20$: rank biserial correlations 0.77–0.87; all $p < 10^{-10}$; Table S14) and prompted further investigation into the structure of these embedding spaces. To quantify representational overlap, we computed cross-space neighborhood similarity (Sim, Equation 4) between the three embedding spaces. Across all tested *k* values and distance metrics, the mean cross-space neighborhood similarity was higher between ProtTucker and ProtT5 than between either and AlphaFold2 (Figures 5B and S5B). Again, the trends were confirmed by statistical testing showing higher cross-space neighborhood similarity for ProtTucker vs. ProtT5 compared to ProtT5 vs. AlphaFold2 (one-sided Wilcoxon signed-rank test at $k = 20$: rank biserial correlation 0.41; $p < 10^{-8}$; Table S15) as well as higher scores for ProtT5 vs. AlphaFold2 compared to

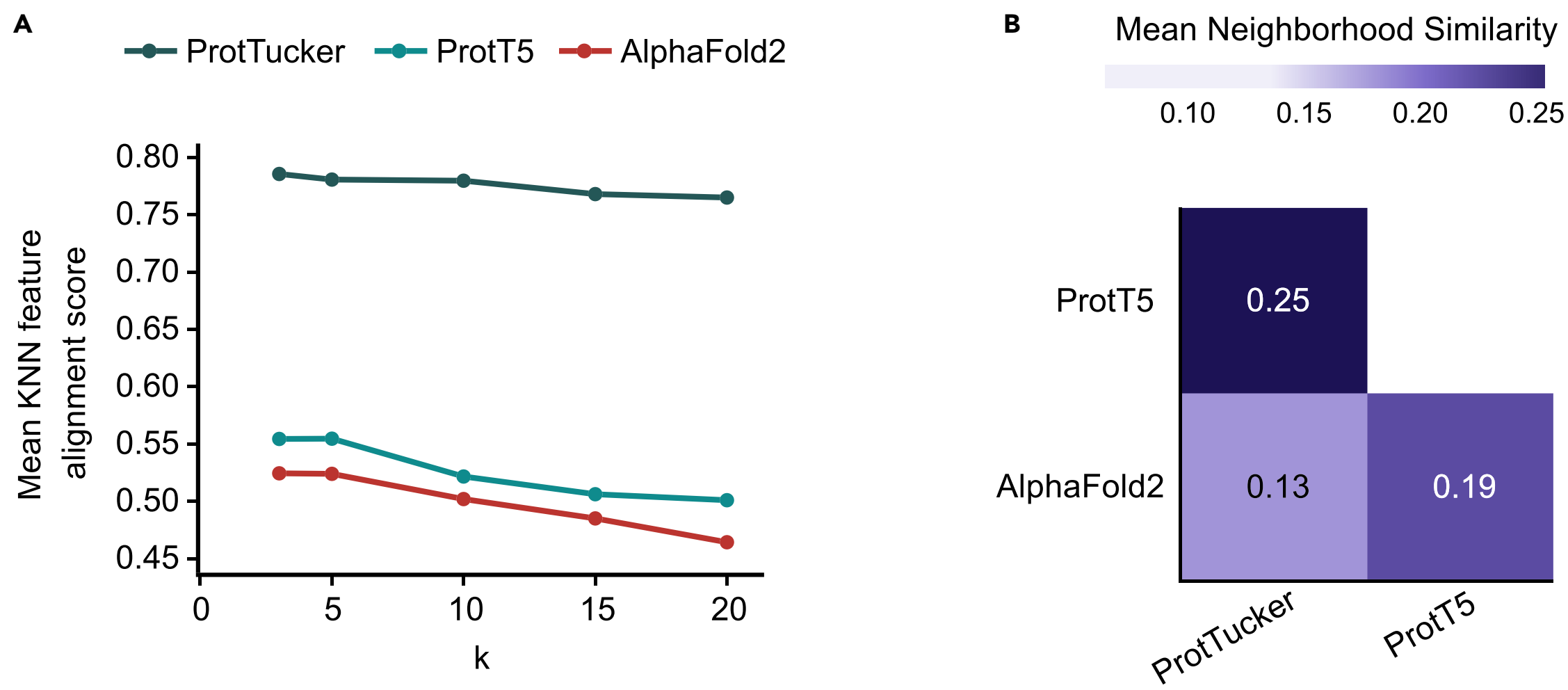

Figure 5. Comparison of ProtT5, ProtTucker (ProtT5 adapted to CATH hierarchy), and AlphaFold2 embeddings for protein structural representation

(A) Mean KNN feature alignment scores (*y* axis) for CATH class feature across varying neighborhood sizes (*k*, *x* axis), shown for cosine distance after mean centering. Each line represents one embedding model. ProtTucker, which is explicitly fine-tuned for CATH structural classification using contrastive learning, consistently shows higher mean KNN feature alignment scores than ProtT5 and AlphaFold2 across all *k* values. AlphaFold2 embeddings show alignment scores comparable to ProtT5 and consistently below ProtTucker, despite being trained on structural data. Results for Manhattan and Euclidean distance are provided in Figure S5.

(B) Mean cross-space neighborhood similarity (shown for $k = 20$, cosine distance) between pairs of embedding models, displayed as a 2×2 matrix where each cell shows the mean similarity between the corresponding model pair. The ProtTucker-ProtT5 pair shows highest mean neighborhood similarity (0.25), indicating that contrastive fine-tuning reshaped the ProtT5 embedding space in a targeted manner while preserving core representational geometry. Both ProtTucker-AlphaFold2 (0.13) and ProtT5-AlphaFold2 (0.19) show lower similarity. Full results across *k* values and distance metrics are provided in Figure S6.

ProtTucker vs. AlphaFold2 (rank biserial correlation 0.71; $p < 10^{-10}$ at $k = 20$; Table S15). This suggests that ProtTucker retains core structural characteristics from ProtT5 while incorporating new task-specific organization, whereas AlphaFold2 encodes structural information in a distinct and different manner.

ProtTucker, explicitly fine-tuned for CATH classification using contrastive learning, shows strong neighborhood alignment for CATH features and serves as a positive control confirming EmmaEmb's ability to detect known embedding structure. Notably, AlphaFold2, despite being trained on structural data, showed no advantage over ProtT5 in neighborhood alignment with CATH classes, and its embedding space was more dissimilar to both ProtT5 and ProtTucker than those two were to each other. This suggests that structural information is encoded through distinct representational routes in sequence-based vs. structure-based models, a finding that would not be accessible from downstream task performance alone. More broadly, these results demonstrate that embedding-space analysis provides a quantitative, architecture-agnostic approach to evaluating how learning objectives shape representations. It can uncover latent similarities and distinctions between models trained with different objectives or architectures, with minimal computational overhead.

Pairwise embedding-space comparison using EmmaEmb reveals and contextualizes differences in information captured by PLMs

In previous sections, we explored embedding spaces starting from specific downstream tasks and comparing model performance in the context of the information encoded in the embeddings. Here, we reversed this perspective and adopted a model-centric view, directly comparing the embedding spaces of models using biologically meaningful features to interpret the models' differences. Toward this end, we analyzed ProtT5 and the more recently released ESM C model,[49] which differ in training strategies, parameter counts, and embedding dimensions (Table 3). Using a set of 446 enzyme proteins from the dataset,[67] we performed pairwise embedding-space comparisons between ProtT5 and ESM C embedding spaces to identify regions of representational divergence and provide context for these differences, leveraging associated biologically interpretable features (Figure 6). Prior to analysis, geometric diagnostics revealed anisotropy in both embedding spaces, which was corrected by applying mean centering of each embedding space (average cosine similarity reduced by mean centering from 0.920 to 0.021 for ESM C and 0.894 to 0.005 for ProtT5, Table S9). Subsequent global correlation analysis of the pairwise cosine distances between the embeddings of the models showed a Spearman's ρ of 0.55 ($p < 0.001$) between all pairwise distances of the ESM C and ProtT5 embeddings, indicating moderate overall similarity in how these two models represent relationships among the protein sequences.

Prior to mean centering, to correct for anisotropy, a visually distinct divergent cluster appeared to be driven primarily by bird protein pairs, an observation that resolved after applying the anisotropy correction (Figure S7), suggesting it was a geometric artifact rather than a genuine representational difference.

After correction, global pairwise distance analysis revealed a cluster of enzyme class B protein pairs showing comparatively low cosine distances in ESM C relative to ProtT5, indicating that ESM C places class B proteins closer together as a group than

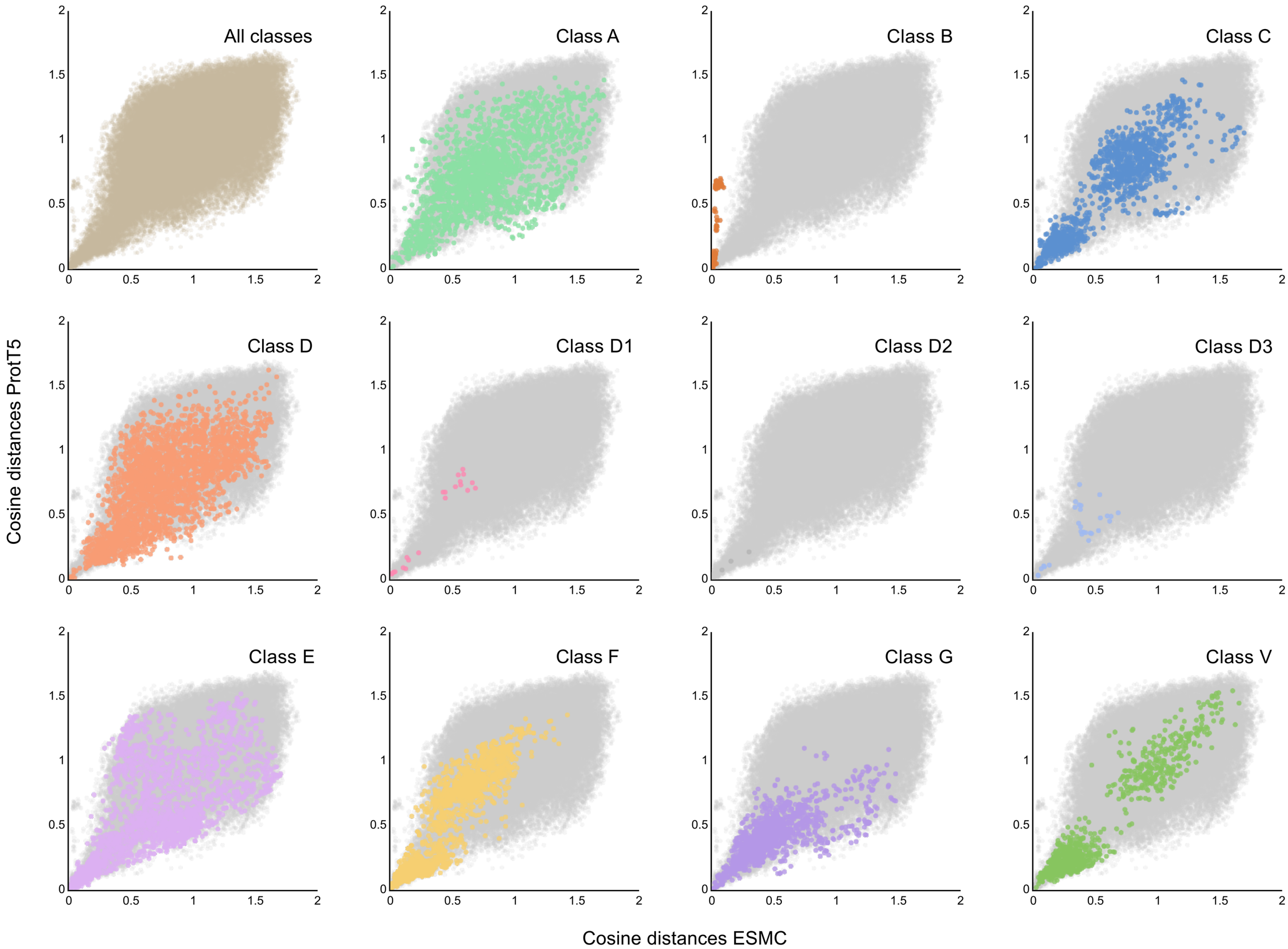

Figure 6. Pairwise embedding-space comparison between ESM C and ProtT5 embeddings on PLA2G2 enzyme proteins

Pairwise cosine distances between PLA2G2 protein embeddings with ESM C (*x* axis) and ProtT5 (*y* axis) for all pairs (all classes, Spearman's $\rho = 0.55$, $p < 0.001$) and for pairs belonging to the same enzyme class (one panel per class, colored points; all other pairs are shown in gray). A distinct cluster of pairs with comparatively low ESM C distances and comparatively high ProtT5 distances is visible and predominantly composed of enzyme class B pairs. This suggests that ESM C places class B proteins closer together than does ProtT5.

does ProtT5 (Figure 6). Enzyme class B (Pla2g2B) has been suggested to be an avian-specific clade of the PLA2G2 family, which derived independently from the enzyme class D2 in birds, is present as a single copy, has a unique N-terminal structure, and whose function remains largely unknown.[67] To assess whether this divergence could be explained by systematic biases, we examined sequence length as a potential confounder. While class B proteins tend to have comparatively shorter sequences relative to other enzyme classes (Figure S9), we found no apparent correlation between pair distance divergence and sequence length across all pairs (Figure S8). Further analysis controlling for additional potential confounders such as taxonomic sampling density and training data overlap would be needed to substantiate the observed class B clustering.

One hypothesis for the class B divergence is that ESM C, which is related to the multi-modal ESM3 model family[4] and incorporates structural information during training, may represent the structurally distinct class B proteins as more similar to each other than a sequence-only model such as ProtT5 would. Given the structural uniqueness of class B within the PLA2G2 family and the limited functional characterization of this clade, this remains a hypothesis for further investigation rather than a conclusion.

While the global pairwise distances between class B proteins differ between the two models, local neighborhood analysis showed that the immediate neighbors of class B proteins are highly similar across the two spaces (Figure S10), indicating that despite their collective repositioning, individual class B proteins retain a consistent local biological context in both models. This dissociation between global and local differences illustrates the complementary information captured by the two levels of analysis and highlights why analyzing embedding spaces at both scales simultaneously is essential for a complete picture of representational differences between models.

DISCUSSION

Despite growing interest in foundation models and their applications, systematic characterization and comparison of these models' embedding spaces remain underexplored in the field

of molecular biology (Table 1). Insights from biological data often emerge from patterns across functionally defined groups of genes or proteins rather than individual data points, calling for tools that support structured, group-level comparison of biological data grounded in features or metadata.[73,74] In this study, we present EmmaEmb, a quantitative, model-agnostic computational framework for analyzing and comparing embedding spaces. By systematically incorporating biological features, EmmaEmb enables researchers to evaluate the organization of embeddings both locally and globally and to contextualize differences between models (Figure 1). Through rigorous experiments in a variety of molecular biology applications, we demonstrate that embedding-space analysis using EmmaEmb can reveal aspects of representational quality that align with transfer learning performance while requiring low computational overhead.

Our findings show that embedding-space neighborhoods can serve as a proxy for downstream predictive power, provided that geometric properties of the embedding space are verified and corrected before analysis (Figures 3 and 4). Neighborhood-based metrics assess whether task-relevant information is readily accessible in the embedding geometry, complementing transfer learning by providing a fast, training-free assessment of representational quality. A high-performing downstream classifier may exploit distributed or non-linear structure that distances between embeddings do not capture, meaning that a low alignment score does not imply absence of information but rather that it may require more complex models to extract. EmmaEmb is therefore best understood as a screening tool that identifies when embedding structure strongly supports a downstream task.

The guided diagnostic workflow implemented in EmmaEmb, covering anisotropy correction, hubness assessment, class imbalance evaluation, and feature noise robustness, provides users with structured, confidence-annotated embedding-space rankings rather than point estimates. A step-by-step walkthrough is provided in Note S1 and the EmmaEmb repository. The residue-binding (binary) task (Figure 3C) illustrates the value of the accompanying diagnostic tools: despite severe class imbalance, feature permutation analyses confirmed that all models' embedding spaces encode binding-relevant information above chance (Figure S3), and progressive rebalancing analyses (Figure S2) identified which embedding-space rankings were stable and which were sensitive to class distribution, allowing conclusions to be drawn selectively and with appropriate robustness assessments.

Notably, for the ligand-binding site (three-class) task (Figures 3D and 4C), which distinguishes between non-binding, regularly binding, and cryptically binding residues, we trained a dedicated MLP classifier on a newly constructed dataset, enabling direct comparison of EmmaEmb rankings against transfer learning performance generated in this study and extending the benchmark to a clinically relevant formulation with direct implications for drug discovery.

Beyond embedding-space ranking, embedding analysis can also monitor how a model's representations change during fine-tuning, an increasingly important capability as foundation models become more specialized.[75] The high cross-space neighborhood similarity between ProtTucker and ProtT5 (Figure 5B) indicates that contrastive fine-tuning introduced targeted task-specific reorganization while retaining the core geometry of the base model. EmmaEmb's cross-space similarity score therefore provides a geometry-aware measure of representational transformation, one that complements downstream performance by quantifying how much the underlying space has changed and what has been preserved. A sharp drop in cross-space similarity relative to the base model may signal that fine-tuning has overwritten general-purpose representations rather than augmenting them, a pattern associated with catastrophic forgetting,[76] while a modest drop combined with improved task alignment suggests targeted, well-controlled adaptation.

The pairwise comparison between ProtT5 and ESM C further illustrates the value of analyzing embedding spaces at both global and local levels simultaneously. The dissociation between global and local differences observed for enzyme class B proteins has direct practical implications for how the two measures should be used in different biological contexts. Global pairwise distance structure is most informative for questions where broad organizational relationships matter, e.g., phylogenetic analysis, taxonomic clustering, or understanding how evolutionary distance is encoded across a protein family.[28,77,78] Local neighborhood structure is most informative where fine-grained similarity is critical, e.g., identifying candidate drug targets by proximity to known binders or retrieving functionally related proteins for annotation transfer.[50,64] Neither failure mode is detectable from a single summary metric, which is why EmmaEmb's combination of global and local analysis is essential for principled model selection. As demonstrated here for sequence length, EmmaEmb's feature-aware design makes it straightforward to investigate confounding effects. The enzyme class B finding should be considered a hypothesis, and future work could leverage this approach to systematically explore further potential confounders.

EmmaEmb is applicable beyond protein embeddings to a wider range of biological data modalities, gene expression, multi-omics, and cell imaging, provided that relevant metadata are available and the embedding space meets the geometric constraints.[79–81] In the context of existing embedding analysis tools (Table 1), EmmaEmb is unique in combining feature-aware analysis, local and global comparisons, geometric diagnostics, and flexible neighborhood-based metrics in a single open-source framework.

We acknowledge that the metadata can be noisy, incomplete, or inconsistent, which can influence the scores computed by EmmaEmb. Further, neighborhood-based metrics are inherently sensitive to class imbalance, a mathematical consequence of minority-class neighborhood availability. To address both challenges directly, EmmaEmb implements progressive class rebalancing and feature perturbation analyses that allow users to quantify the influence of class distribution and metadata quality on embedding-space rankings and to report conclusions with explicit robustness assessments. We note that the benchmark datasets used in this study vary in the stringency and metrics applied to define train and test splits, ranging from published splits inherited from the original studies to sequence identity filtering applied in the newly constructed ligand-binding site dataset. Additional splitting strategies based on protein family, fold type, or binding-pocket similarity would provide further rigor.

The analyses presented here are based on embeddings extracted from a single, fixed representation layer per model. Applying EmmaEmb across layers within a single model is a natural direction for future work. Mean centering was applied in most embedding spaces analyzed in this study, underscoring that anisotropy correction is an important prerequisite for meaningful distance-based analysis; further investigation into anisotropy correction strategies is an interesting direction for future work. Additionally, EmmaEmb is well suited to investigate how neighborhood structure changes with commonly applied preprocessing steps such as dimensionality reduction or feature scaling.

More broadly, EmmaEmb contributes to the growing need for interpretability and trustworthiness in deep learning for molecular biology. By elucidating what biological information is captured, where misclassifications originate, and how representations change with fine-tuning, it supports principled decisions about embedding-space ranking, prioritization, and deployment. We demonstrated this utility through critical applications such as drug discovery, where opaque model behavior can undermine confidence in predictions. Future directions include applying EmmaEmb to other high-stakes applications such as clinical variant interpretation. Extensions could integrate additional interpretability tools such as sparse autoencoders[19] or hierarchical visualization,[28] and EmmaEmb's modular design supports community-driven additions.

In summary, EmmaEmb provides a unified and extensible framework for quantitative comparison of embedding spaces, enabling principled model evaluation, misclassification analysis, and representational monitoring across fine-tuning. As the landscape of foundation models in molecular biology continues to expand, such tools will be indispensable for bridging the gap between raw predictive performance and biologically meaningful understanding.

RESOURCE AVAILABILITY

Lead contact

Requests for further information and resources should be directed to and will be fulfilled by the lead contact, Sumaiya Iqbal (sumaiya@broadinstitute.org).

Materials availability

All datasets used in this study are publicly available.

Data and code availability

All original code regarding the EmmaEmb library, including scripts for data preprocessing and figure reproduction, has been deposited at https://github.com/broadinstitute/EmmaEmb and archived on Zenodo: https://doi.org/10.5281/zenodo.21246289.[41] Code for the ligand-binding site classification models and associated figures is available at https://github.com/skrhakv/emb-space-analysis and archived on Zenodo: https://doi.org/10.5281/zenodo.21246342.[71] The newly curated ligand-binding site dataset including train, evaluation, and test splits, sequence IDs, accession lists, and random seeds has been deposited on Zenodo: https://doi.org/10.5281/zenodo.21246325.[53]

ACKNOWLEDGMENTS

The project was supported by the Designing for Sustainability research program, a joint initiative of the Morningside Academy for Design at MIT and the Hasso Plattner Institute, funded by the Hasso Plattner Foundation. This work was also supported by the Deutsche Forschungsgemeinschaft (DFG, German Research Foundation; project number 459422098). We express our gratitude to Eugenia Alleva, Jannis Baum, Katharina Baum, Jakub Bartoszewicz, Hilary Finucane, Theresa Hradilak, Marta Lemanczyk, and Melania Nowicka for their valuable discussions and insightful input regarding this project.

AUTHOR CONTRIBUTIONS

Conceptualization, P.F.R., P.Y.S., J.F.S., and S.I.; data curation, P.F.R., V.S., and J.F.S.; formal analysis, P.F.R. and V.S.; funding acquisition, H.O.H. and S.I.; investigation, P.F.R. and V.S.; methodology, P.F.R., P.Y.S., B.Y.R., and S.I.; project administration, S.I.; resources, C.W.C., B.Y.R., H.O.H., and S.I.; software, P.F.R. and J.F.S.; supervision, H.O.H. and S.I.; validation, P.F.R. and V.S.; visualization, P.F.R. and V.S.; writing – original draft, P.F.R. and P.Y.S.; writing – review & editing, P.F.R., V.S., P.Y.S., J.F.S., C.W.C., B.Y.R., H.O.H., and S.I.

DECLARATION OF INTERESTS

No competing interests are declared.

DECLARATION OF GENERATIVE AI AND AI-ASSISTED TECHNOLOGIES IN THE WRITING PROCESS

During the preparation of this work, the authors used ChatGPT (GPT-4 and GPT-5 by OpenAI) and Claude Sonnet 4.6 to revise portions of the text for clarity. After using this tool or service, the authors reviewed and edited the content as needed and take full responsibility for the content of the publication.

SUPPLEMENTAL INFORMATION

Supplementary data related to this article can be found online at https://doi.org/10.1016/j.patter.2026.101635.

Received: December 19, 2025
Revised: April 15, 2026
Accepted: July 15, 2026
Published: August 12, 2026

REFERENCES

1. Jumper, J., Evans, R., Pritzel, A., Green, T., Figurnov, M., Ronneberger, O., Tunyasuvunakool, K., Bates, R., Žídek, A., Potapenko, A., et al. (2021). Highly accurate protein structure prediction with AlphaFold. Nature *596*, 583–589. https://doi.org/10.1038/s41586-021-03819-2.
2. Elnaggar, A., Heinzinger, M., Dallago, C., Rehawi, G., Wang, Y., Jones, L., Gibbs, T., Feher, T., Angerer, C., Steinegger, M., et al. (2022). ProtTrans: Toward Understanding the Language of Life Through Self-Supervised Learning. IEEE Trans. Pattern Anal. Mach. Intell. *44*, 7112–7127. https://doi.org/10.1109/TPAMI.2021.3095381.
3. Lin, Z., Akin, H., Rao, R., Hie, B., Zhu, Z., Lu, W., Smetanin, N., Verkuil, R., Kabeli, O., Shmueli, Y., et al. (2023). Evolutionary-scale prediction of atomic level protein structure with a language model. Science *379*, 1123–1130. https://doi.org/10.1126/science.ade2574.
4. Hayes, T., Rao, R., Akin, H., Sofroniew, N.J., Oktay, D., Lin, Z., Verkuil, R., Tran, V.Q., Deaton, J., Wiggert, M., et al. (2025). Simulating 500 million years of evolution with a language model. Science *387*, 850–858. https://doi.org/10.1126/science.ads0018.
5. Kulmanov, M., Guzmán-Vega, F.J., Duek Roggli, P., Lane, L., Arold, S.T., and Hoehndorf, R. (2024). Protein function prediction as approximate semantic entailment. Nat. Mach. Intell. *6*, 220–228. https://doi.org/10.1038/s42256-024-00795-w.
6. Heinzinger, M., Weissenow, K., Sanchez, J.G., Henkel, A., Mirdita, M., Steinegger, M., and Rost, B. (2024). Bilingual language model for protein sequence and structure. NAR Genom. Bioinf. *6*, lqae150. https://doi.org/10.1093/nargab/lqae150.

7. Shrestha, P., Kandel, J., Tayara, H., and Chong, K.T. (2024). Post-translational modification prediction via prompt-based fine-tuning of a GPT-2 model. Nat. Commun. *15*, 6699. https://doi.org/10.1038/s41467-024-51071-9.

8. Zhang, Q., Chen, W., Qin, M., Wang, Y., Pu, Z., Ding, K., Liu, Y., Zhang, Q., Li, D., Li, X., et al. (2025). Integrating protein language models and automatic biofoundry for enhanced protein evolution. Nat. Commun. *16*, 1553. https://doi.org/10.1038/s41467-025-56751-8.

9. Bikias, T., Stamkopoulos, E., and Reddy, S.T. (2025). PLMFit: benchmarking transfer learning with protein language models for protein engineering. Briefings Bioinf. *26*, bbaf381. https://doi.org/10.1093/bib/bbaf381.

10. Ding, F., and Steinhardt, J. (2024). Protein language models are biased by unequal sequence sampling across the tree of life. Preprint at bioRxiv, https://doi.org/10.1101/2024.03.07.584001.

11. Busch, F., Hoffmann, L., Rueger, C., van Dijk, E.H., Kader, R., Ortiz-Prado, E., Makowski, M.R., Saba, L., Hadamitzky, M., Kather, J.N., et al. (2025). Current applications and challenges in large language models for patient care: a systematic review. Commun. Med. *5*, 26. https://doi.org/10.1038/s43856-024-00717-2.

12. Lu, J., Choi, K., Eremeev, M., Gobburu, J., Goswami, S., Liu, Q., Mo, G., Musante, C.J., and Shahin, M.H. (2025). Large Language Models and Their Applications in Drug Discovery and Development: A Primer. Clin. Transl. Sci. *18*, e70205. https://doi.org/10.1111/cts.70205.

13. Shool, S., Adimi, S., Saboori Amleshi, R., Bitaraf, E., Golpira, R., and Tara, M. (2025). A systematic review of large language model (LLM) evaluations in clinical medicine. BMC Med. Inf. Decis. Making *25*, 117. https://doi.org/10.1186/s12911-025-02954-4.

14. Marquet, C., Heinzinger, M., Olenyi, T., Dallago, C., Erckert, K., Bernhofer, M., Nechaev, D., and Rost, B. (2022). Embeddings from protein language models predict conservation and variant effects. Hum. Genet. *141*, 1629–1647. https://doi.org/10.1007/s00439-021-02411-y.

15. Stärk, H., Dallago, C., Heinzinger, M., and Rost, B. (2021). Light attention predicts protein location from the language of life. Bioinform. Adv. *1*, vbab035. https://doi.org/10.1093/bioadv/vbab035.

16. Littmann, M., Heinzinger, M., Dallago, C., Weissenow, K., and Rost, B. (2021). Protein embeddings and deep learning predict binding residues for various ligand classes. Sci. Rep. *11*, 23916. https://doi.org/10.1038/s41598-021-03431-4.

17. Özçelik, R., Ruiter, S. de, and Grisoni, F. (2025). Look the Other Way: Designing "Positive" Molecules with Negative Data via Task Arithmetic. Preprint at arXiv. https://doi.org/10.48550/arXiv.2507.17876.

18. Kilgore, H.R., Chinn, I., Mikhael, P.G., Mitnikov, I., Van Dongen, C., Zylberberg, G., Afeyan, L., Banani, S.F., Wilson-Hawken, S., Lee, T.I., et al. (2025). Protein codes promote selective subcellular compartmentalization. Science *387*, 1095–1101. https://doi.org/10.1126/science.adq2634.

19. Simon, E., and Zou, J. (2025). InterPLM: discovering interpretable features in protein language models via sparse autoencoders. Nat. Methods *22*, 2107–2117. https://doi.org/10.1038/s41592-025-02836-7.

20. Garcia, E.N.V., and Ansuini, A. (2025). Interpreting and Steering Protein Language Models through Sparse Autoencoders. Preprint at arXiv. https://doi.org/10.48550/arXiv.2502.09135.

21. Vylomova, E., Rimell, L., Cohn, T., and Baldwin, T. (2016). Take and Took, Gaggle and Goose, Book and Read: Evaluating the Utility of Vector Differences for Lexical Relation Learning. In Proceedings of the 54th Annual Meeting of the Association for Computational Linguistics (Volume 1: Long Papers), K. Erk and N.A. Smith, eds. (Association for Computational Linguistics), pp. 1671–1682. https://doi.org/10.18653/v1/P16-1158.

22. Bolukbasi, T., Chang, K.-W., Zou, J.Y., Saligrama, V., and Kalai, A.T. (2016). Man is to Computer Programmer as Woman is to Homemaker? Debiasing Word Embeddings,Preprint at arXiv.

23. Hangya, V., Severini, S., Ralev, R., Fraser, A., and Schütze, H. (2023). Multilingual Word Embeddings for Low-Resource Languages using Anchors and a Chain of Related Languages. In Proceedings of the 3rd Workshop on Multi-lingual Representation Learning (MRL), D. Ataman, ed. (Association for Computational Linguistics), pp. 95–105. https://doi.org/10.18653/v1/2023.mrl-1.8.

24. Aldeghi, M., Graff, D.E., Frey, N., Morrone, J.A., Pyzer-Knapp, E.O., Jordan, K.E., and Coley, C.W. (2022). Roughness of Molecular Property Landscapes and Its Impact on Modellability. J. Chem. Inf. Model. *62*, 4660–4671. https://doi.org/10.1021/acs.jcim.2c00903.

25. Graff, D.E., Pyzer-Knapp, E.O., Jordan, K.E., Shakhnovich, E.I., and Coley, C.W. (2023). Evaluating the roughness of structure–property relationships using pretrained molecular representations. Dig. Dis. *2*, 1452–1460. https://doi.org/10.1039/D3DD00088E.

26. Altenburg, T., Muth, T., Zalm, P. van, Steen, H., and Renard, B.Y. (2026). Foundation model enables interpretable open and error-tolerant searching for mass spectrometry-based proteomics. Bioinf. *42*, btag297. https://doi.org/10.1093/bioinformatics/btag297.

27. Prabakaran, R., and Bromberg, Y. (2026). Quantifying uncertainty in protein representations across models and tasks. Nat. Methods *23*, 796–804. https://doi.org/10.1038/s41592-026-03028-7.

28. Yeung, W., Zhou, Z., Mathew, L., Gravel, N., Taujale, R., O'Boyle, B., Salcedo, M., Venkat, A., Lanzilotta, W., Li, S., and Kannan, N. (2023). Tree visualizations of protein sequence embedding space enable improved functional clustering of diverse protein superfamilies. Briefings Bioinf. *24*, bbac619. https://doi.org/10.1093/bib/bbac619.

29. Shiebler, D. (2018). repcomp. https://github.com/dshieble/RepresentationComparison.

30. Heimerl, F., Kralj, C., Moller, T., and Gleicher, M. (2022). embComp: Visual Interactive Comparison of Vector Embeddings. IEEE Trans. Vis. Comput. Graph. *28*, 2953–2969. https://doi.org/10.1109/TVCG.2020.3045918.

31. Boggust, A., Carter, B., and Satyanarayan, A. (2022). Embedding Comparator: Visualizing Differences in Global Structure and Local Neighborhoods via Small Multiples. In Proceedings of the 27th International Conference on Intelligent User Interfaces IUI '22 (Association for Computing Machinery), pp. 746–766. https://doi.org/10.1145/3490099.3511122.

32. PentoAI (2022). Vectory. https://github.com/pentoai/vectory.

33. Sivaraman, V., Wu, Y., and Perer, A. (2022). Emblaze: Illuminating Machine Learning Representations through Interactive Comparison of Embedding Spaces. In Proceedings of the 27th International Conference on Intelligent User Interfaces IUI '22 (Association for Computing Machinery), pp. 418–432. https://doi.org/10.1145/3490099.3511137.

34. France-Travail (2022). EmbCompare. https://github.com/France-Travail/embcompare.

35. Turura, Y., Friedman, S.F., Cremer, A., Maddah, M., and Tonekaboni, S. (2025). The Latentverse: An Open-Source Benchmarking Toolkit for Evaluating Latent Representations. In Proceedings of the Sixth Conference on Health, Inference, and Learning (PMLR), pp. 708–719.

36. Tenenbaum, J.B., de Silva, V., and Langford, J.C. (2000). A Global Geometric Framework for Nonlinear Dimensionality Reduction. Science *290*, 2319–2323. https://doi.org/10.1126/science.290.5500.2319.

37. Roweis, S.T., and Saul, L.K. (2000). Nonlinear dimensionality reduction by locally linear embedding. Science *290*, 2323–2326. https://doi.org/10.1126/science.290.5500.2323.

38. Kruskal, J.B. (1964). Nonmetric multidimensional scaling: A numerical method. Psychometrika *29*, 115–129. https://doi.org/10.1007/BF02289694.

39. Li, Z., Ding, Q., and Zhang, W. (2011). A Comparative Study of Different Distances for Similarity Estimation. Intelligent Computing and Information Science I *134*, 483–488. https://doi.org/10.1007/978-3-642-18129-0_75.

40. Wolf, F.A., Angerer, P., and Theis, F.J. (2018). SCANPY: large-scale single-cell gene expression data analysis. Genome Biol. *19*, 15. https://doi.org/10.1186/s13059-017-1382-0.

41. Rissom, P.F., and Safer, J.F. (2026). broadinstitute/EmmaEmb: EmmaEmb: A Quantitative Framework for Analyzing Embedding Spaces in Molecular Biology (Zenodo). https://doi.org/10.5281/zenodo.21246289.

42. Pedregosa, F., Varoquaux, G., Gramfort, A., Michel, V., Thirion, B., Grisel, O., Blondel, M., Prettenhofer, P., Weiss, R., Dubourg, V., et al. (2011). Scikit-learn: Machine Learning in Python. J. Mach. Learn. Res. *12*, 2825–2830.

43. Plotly Technologies Inc. (2015). Plotly: Interactive Graphing Libraries. https://plot.ly

44. Bernhardsson, E. (2019). ANNOY library. https://github.com/spotify/annoy.

45. Ethayarajh, K. (2019). How Contextual are Contextualized Word Representations? Comparing the Geometry of BERT, ELMo, and GPT-2 Embeddings. In Proceedings of the 2019 Conference on Empirical Methods in Natural Language Processing and the 9th International Joint Conference on Natural Language Processing (EMNLP-IJCNLP), K. Inui, J. Jiang, V. Ng, and X. Wan, eds. (Association for Computational Linguistics), pp. 55–65. https://doi.org/10.18653/v1/D19-1006.

46. Radovanovic, M., Nanopoulos, A., and Ivanovic, M. (2010). Hubs in space: Popular nearest neighbors in high-dimensional data. Journal of machine learning research *11*, 2487–2531.

47. Feldbauer, R., Leodolter, M., Plant, C., and Flexer, A. (2018). Fast Approximate Hubness Reduction for Large High-Dimensional Data. In 2018 IEEE International Conference on Big Knowledge (ICBK), pp. 358–367. https://doi.org/10.1109/ICBK.2018.00055.

48. Elnaggar, A., Essam, H., Salah-Eldin, W., Moustafa, W., Elkerdawy, M., Rochereau, C., and Rost, B. (2023). Ankh: Optimized Protein Language Model Unlocks General-Purpose Modelling. Preprint at arXiv. https://doi.org/10.48550/arXiv.2301.06568.

49. ESM Team (2024). ESM Cambrian: Revealing the mysteries of proteins with unsupervised learning. https://evolutionaryscale.ai/blog/esm-cambrian.

50. Heinzinger, M., Littmann, M., Sillitoe, I., Bordin, N., Orengo, C., and Rost, B. (2022). Contrastive learning on protein embeddings enlightens midnight zone. NAR Genom. Bioinf. *4*, lqac043. https://doi.org/10.1093/nargab/lqac043.

51. Almagro Armenteros, J.J., Sønderby, C.K., Sønderby, S.K., Nielsen, H., and Winther, O. (2017). DeepLoc: prediction of protein subcellular localization using deep learning. Bioinformatics *33*, 3387–3395. https://doi.org/10.1093/bioinformatics/btx431.

52. Ben Chorin, A., Masrati, G., Kessel, A., Narunsky, A., Sprinzak, J., Lahav, S., Ashkenazy, H., and Ben-Tal, N. (2020). ConSurf-DB: An accessible repository for the evolutionary conservation patterns of the majority of PDB proteins. Protein Sci. *29*, 258–267. https://doi.org/10.1002/pro.3779.

53. Škrhák, V. (2026). Training data and trained model for EmmaEmb application to residue binding prediction. Zenodo. https://doi.org/10.5281/zenodo.21246325.

54. Škrhák, V., Novotný, M., Feidakis, C.P., Krivák, R., and Hoksza, D. (2024). CryptoBench: cryptic protein–ligand binding sites dataset and benchmark. Bioinformatics *41*, btae745. https://doi.org/10.1093/bioinformatics/btae745.

55. Škrhák, V., and Hoksza, D. (2026). Cryptobench (OSF)). https://doi.org/10.17605/OSF.IO/PZ4A9.

56. Desaphy, J., Bret, G., Rognan, D., and Kellenberger, E. (2015). sc-PDB: a 3D-database of ligandable binding sites–10 years on. Nucleic Acids Res. *43*, D399–D404. https://doi.org/10.1093/nar/gku928.

57. Kellenberger, E., Muller, P., Schalon, C., Bret, G., Foata, N., and Rognan, D. (2006). sc-PDB: an annotated database of druggable binding sites from the Protein Data Bank. J. Chem. Inf. Model. *46*, 717–727. https://doi.org/10.1021/ci050372x.

58. Meslamani, J., Rognan, D., and Kellenberger, E. (2011). sc-PDB: a database for identifying variations and multiplicity of "druggable" binding sites in proteins. Bioinformatics *27*, 1324–1326. https://doi.org/10.1093/bioinformatics/btr120.

59. Škrhák, V. (2026). scPDB-Enhanced: An expansion of scPDB including all experimentally observed binding sites. https://doi.org/10.5281/zenodo.18271517.

60. Škrhák, V., Polák, L., Novotný, M., and Hoksza, D. (2026). Seq2Pocket: Augmenting protein language models for spatially consistent binding site prediction. Preprint at bioRxiv. https://doi.org/10.64898/2026.01.28.702257.

61. Feidakis, C.P., Krivak, R., Hoksza, D., and Novotny, M. (2022). AHoJ: rapid, tailored search and retrieval of apo and holo protein structures for user-defined ligands. Bioinformatics *38*, 5452–5453. https://doi.org/10.1093/bioinformatics/btac701.

62. Feidakis, C.P., Krivak, R., Hoksza, D., and Novotny, M. (2024). AHoJ-DB: A PDB-wide Assignment of apo & holo Relationships Based on Individual Protein-Ligand Interactions. J. Mol. Biol. *436*, 168545. https://doi.org/10.1016/j.jmb.2024.168545.

63. Utgés, J.S., and Barton, G.J. (2024). Comparative evaluation of methods for the prediction of protein–ligand binding sites. J. Cheminf. *16*, 126. https://doi.org/10.1186/s13321-024-00923-z.

64. Utgés, J.S., MacGowan, S.A., and Barton, G.J. (2025). LIGYSIS-web: a resource for the analysis of protein-ligand binding sites. Nucleic Acids Res. *53*, W351–W360. https://doi.org/10.1093/nar/gkaf411.

65. Steinegger, M., and Söding, J. (2017). MMseqs2 enables sensitive protein sequence searching for the analysis of massive data sets. Nat. Biotechnol. *35*, 1026–1028. https://doi.org/10.1038/nbt.3988.

66. Orengo, C.A., Michie, A.D., Jones, S., Jones, D.T., Swindells, M.B., and Thornton, J.M. (1997). CATH–a hierarchic classification of protein domain structures. Structure *5*, 1093–1108. https://doi.org/10.1016/s0969-2126(97)00260-8.

67. Koludarov, I., Jackson, T.N., Suranse, V., Pozzi, A., Sunagar, K., and Mikheyev, A.S. (2020). Reconstructing the evolutionary history of a functionally diverse gene family reveals complexity at the genetic origins of novelty. Preprint at bioRxiv. https://doi.org/10.1101/583344.

68. Pla2g2 dataset (2024). ProtSpace GitHub. https://github.com/tsenoner/protspace/tree/main/data/Pla2g2.

69. Brandes, N., Goldman, G., Wang, C.H., Ye, C.J., and Ntranos, V. (2023). Genome-wide prediction of disease variant effects with a deep protein language model. Nat. Genet. *55*, 1512–1522. https://doi.org/10.1038/s41588-023-01465-0.

70. Mirdita, M., Schütze, K., Moriwaki, Y., Heo, L., Ovchinnikov, S., and Steinegger, M. (2022). ColabFold: making protein folding accessible to all. Nat. Methods *19*, 679–682. https://doi.org/10.1038/s41592-022-01488-1.

71. Škrhák, V. (2026). EmmaEmb application to binding site prediction. Zenodo. https://doi.org/10.5281/zenodo.21246342.

72. Vallat, R. (2018). Pingouin: statistics in Python. J. Open Source Softw. *3*, 1026. https://doi.org/10.21105/joss.01026.

73. Ragueneau, E., Gong, C., Sinquin, P., Sevilla, C., Beavers, D., Grentner, A., Griss, J., Hogue, G.F.J., Li, N.T., Matthews, L., et al. (2026). The Reactome Knowledgebase 2026. Nucleic Acids Res. *54*, D673–D681. https://doi.org/10.1093/nar/gkaf1223.

74. Subramanian, A., Tamayo, P., Mootha, V.K., Mukherjee, S., Ebert, B.L., Gillette, M.A., Paulovich, A., Pomeroy, S.L., Golub, T.R., Lander, E.S., and Mesirov, J.P. (2005). Gene set enrichment analysis: A knowledge-based approach for interpreting genome-wide expression profiles. Proc. Natl. Acad. Sci. USA *102*, 15545–15550. https://doi.org/10.1073/pnas.0506580102.

75. Schmirler, R., Heinzinger, M., and Rost, B. (2024). Fine-tuning protein language models boosts predictions across diverse tasks. Nat. Commun. *15*, 7407. https://doi.org/10.1038/s41467-024-51844-2.

76. Kirkpatrick, J., Pascanu, R., Rabinowitz, N., Veness, J., Desjardins, G., Rusu, A.A., Milan, K., Quan, J., Ramalho, T., Grabska-Barwinska, A., et al. (2017). Overcoming catastrophic forgetting in neural networks. Proc. Natl. Acad. Sci. USA *114*, 3521–3526. https://doi.org/10.1073/pnas.1611835114.

77. Ektefaie, Y., Shen, A., Jain, L., Farhat, M., and Zitnik, M. (2025). Evolutionary Reasoning Does Not Arise in Standard Usage of Protein Language Models. bioRxiv, 2025.01.17.633626. https://doi.org/10.1101/2025.01.17.633626.

78. Mock, F., Kretschmer, F., Kriese, A., Böcker, S., and Marz, M. (2022). Taxonomic classification of DNA sequences beyond sequence similarity using deep neural networks. Proc. Natl. Acad. Sci. USA *119*, e2122636119. https://doi.org/10.1073/pnas.2122636119.

79. Castellano-Escuder, P., Zachman, D.K., Han, K., and Hirschey, M.D. (2025). GAUDI: interpretable multi-omics integration with UMAP embeddings and density-based clustering. Nat. Commun. *16*, 5771. https://doi.org/10.1038/s41467-025-60822-1.

80. Marks, M., Israel, U., Dilip, R., Li, Q., Yu, C., Laubscher, E., Iqbal, A., Pradhan, E., Ates, A., Abt, M., et al. (2025). CellSAM: a foundation model for cell segmentation. Nat. Methods *22*, 2585–2593. https://doi.org/10.1038/s41592-025-02879-w.

81. Theodoris, C.V., Xiao, L., Chopra, A., Chaffin, M.D., Al Sayed, Z.R., Hill, M.C., Mantineo, H., Brydon, E.M., Zeng, Z., Liu, X.S., and Ellinor, P.T. (2023). Transfer learning enables predictions in network biology. Nature *618*, 616–624. https://doi.org/10.1038/s41586-023-06139-9.

Patterns, Volume *7*

Supplemental information

EmmaEmb: A quantitative framework for analyzing embedding spaces in molecular biology

Pia Francesca Rissom, Vít Škrhák, Paulo Yanez Sarmiento, Jordan F. Safer, Connor W. Coley, Bernhard Y. Renard, Henrike O. Heyne, and Sumaiya Iqbal

Table of Contents

Supplemental Figures....3
Figure S1: Mean KNN feature alignment scores across distance metrics and k-values without mean centering....3
Figure S2: Class rebalancing analysis for residue binding (binary)....5
Figure S3: Feature perturbation robustness analysis for residue binding (binary)....6
Figure S4: Fractional class distributions from transfer learning and embedding space neighborhood analysis across three tasks....7
Figure S5: Mean KNN feature alignment scores for the CATH class feature across Manhattan and Euclidean distance metrics....9
Figure S6: Mean cross-space neighborhood similarity matrices between ProtTucker, ProtT5, and AlphaFold2 across k values and distance metrics....10
Figure S7: Pairwise cosine distances between PLA2G2 protein embeddings in ESM C and ProtT5, colored by species, before and after mean centering....11
Figure S8: Pairwise cosine distances after mean centering between PLA2G2 protein embeddings in ESM C and ProtT5, colored by binned sequence length....12
Figure S9: Sequence length distribution per PLA2G2 enzyme class....12
Figure S10: Cross-space neighborhood similarity between ESM C and ProtT5 embeddings of PLA2G2 proteins, stratified by species, sequence length, and enzyme class....13
Supplemental Tables....14
Table S1: Protein counts per subcellular localization class in the subcellular localization dataset....14
Table S2: Residue counts per conservation class in the residue conservation dataset....14
Table S3: Residue counts per residue binding class in the residue binding (binary) dataset....14
Table S4: Protein counts per CATH class in the protein structure (CATH) dataset....15
Table S5: Protein counts per class in the Phospholipase A2 family (PLA2G2) dataset....16
Table S6: Residue counts per ligand binding class in the ligand binding site (three-class) dataset....17
Table S7: Overview of protein language model benchmark tasks, datasets, and reference....18
Table S8: Performance of transfer learning model for ligand binding site (three-class)....19
Table S9: Embedding space geometry diagnostics for all model-dataset combinations....20
Table S10: One-sided Wilcoxon signed-rank tests for KNN feature alignment scores at k = 100 for the subcellular localization dataset after mean centering of the embeddings....21
Table S11: One-sided Wilcoxon signed-rank tests for KNN feature alignment scores at k = 100 for the residue conservation dataset after mean centering of the embeddings....21
Table S12: One-sided Wilcoxon signed-rank tests for KNN feature alignment scores at k = 100 for the residue binding (binary) dataset after mean centering of the embeddings....21
Table S13: One-sided Wilcoxon signed-rank tests for KNN feature alignment scores at k = 100 for the ligand binding site (three-class) dataset after mean centering of the embeddings....21
Table S14: One-sided Wilcoxon signed-rank tests for KNN feature alignment scores at k = 20 for the protein structure (CATH) dataset after mean centering of the embeddings....22
Table S15: One-sided Wilcoxon signed-rank tests for neighborhood similarity scores at k = 20 for the protein structure (CATH) dataset after mean centering of the embeddings....22
Supplemental Notes....23
S1: EmmaEmb guided diagnostic workflow for embedding space ranking....23

References 27

Supplemental Figures

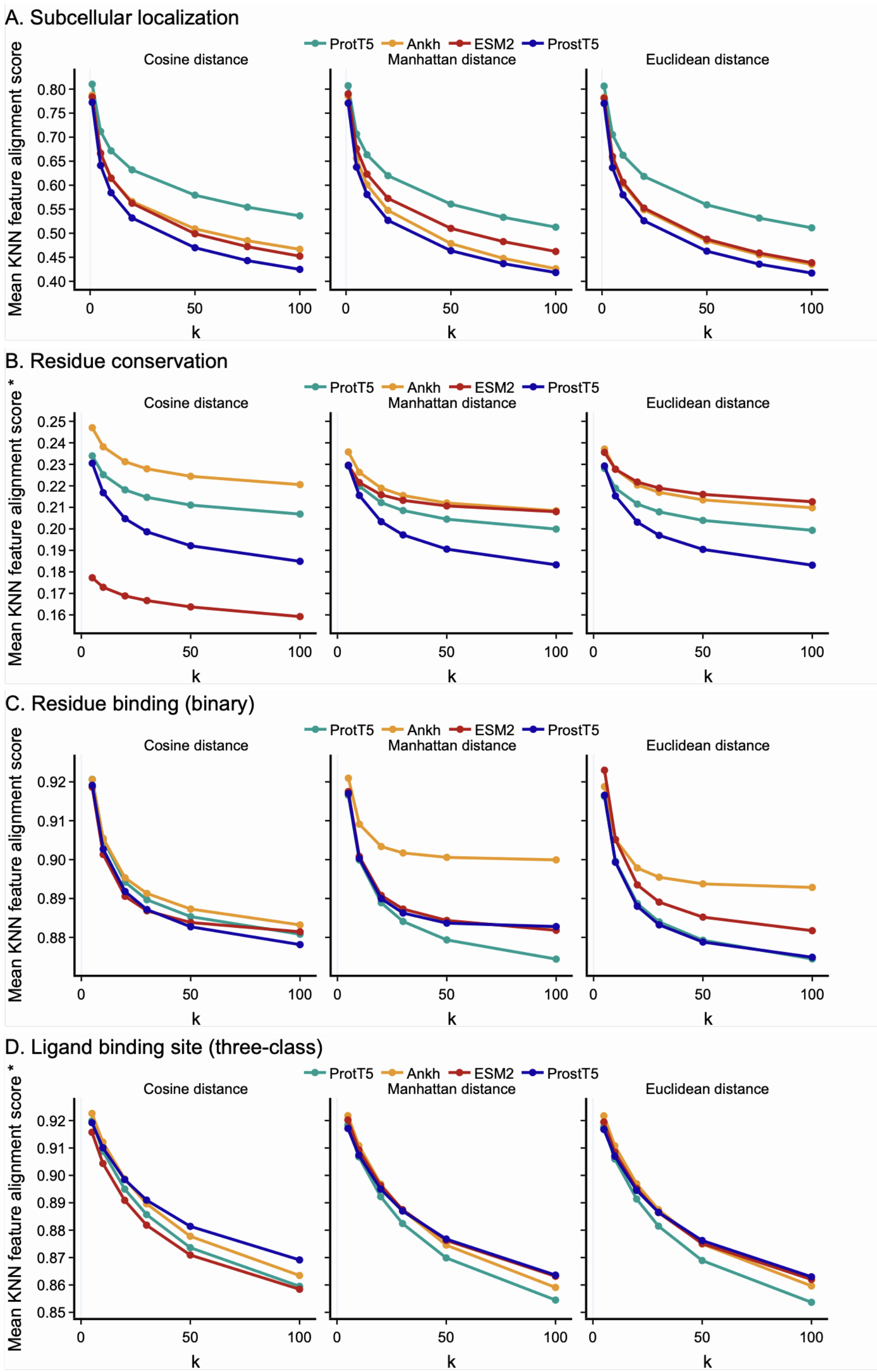

Figure S1: Mean KNN feature alignment scores across distance metrics and k-values without mean centering.
Mean KNN feature alignment scores (y-axis, EmmaEmb) across varying k-values (x-axis) are shown for three distance metrics (columns: cosine, Euclidean, Manhattan) and four PLM models (colors: ProtT5, Ankh, ESM2, ProstT5), prior to mean centering correction. An asterisk on the y-axis label indicates that KNN neighborhoods were approximated using the ANNOY algorithm. Results are shown for the datasets

(A) subcellular localization, (B) residue conservation, (C) residue binding (binary), and (D) ligand binding site (three-class). Comparison with Figure 3 (cosine distance, after mean centering) illustrates the effect of geometric correction on embedding space rankings. Notably for residue conservation and residue binding (binary), where embedding space rankings shift to align with transfer learning performance only after mean centering is applied.

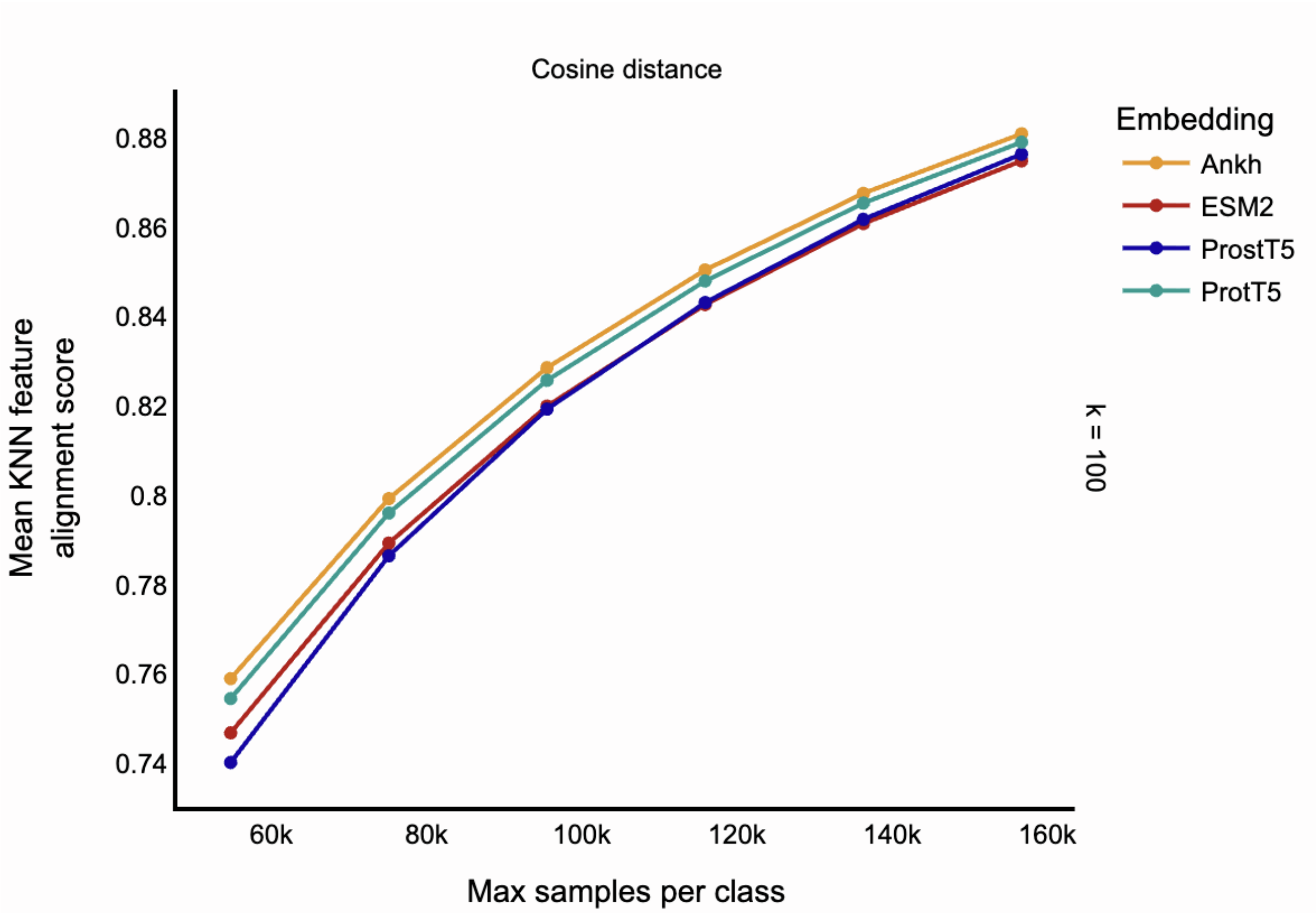

Figure S2: Class rebalancing analysis for residue binding (binary)
Mean KNN feature alignment scores (y-axis, EmmaEmb, cosine distance, k = 100, after mean centering) for four PLMs (Ankh, ESM2, ProstT5, ProtT5) as the maximum number of samples per class is progressively decreasing toward class balance by down sampling the majority class (x-axis). As the majority class is reduced, alignment scores decrease across all models, reflecting the reduced availability of same-class neighbors for the dominant non-binding class.

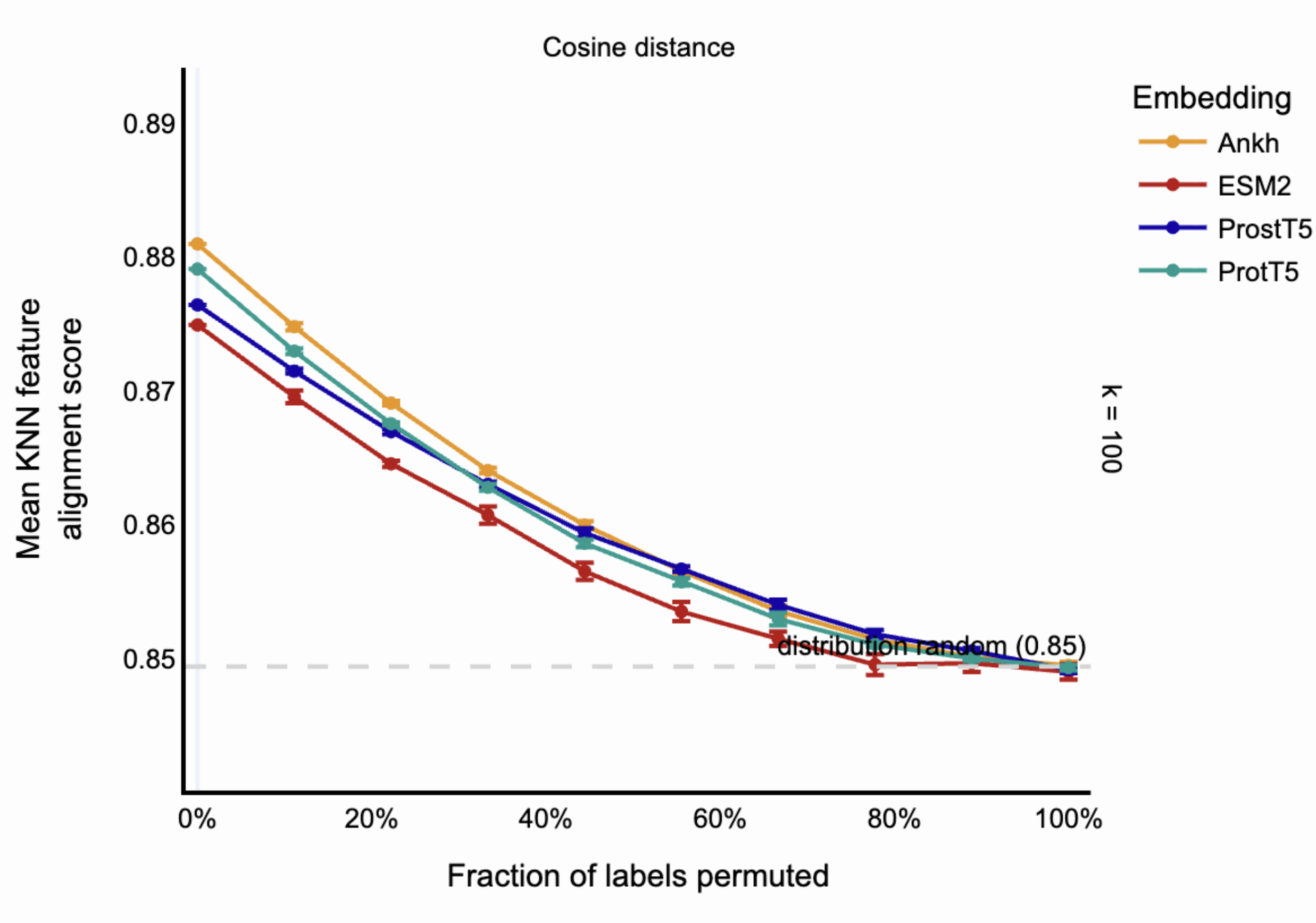

Figure S3: Feature perturbation robustness analysis for residue binding (binary)
Mean KNN feature alignment scores (y-axis; cosine distance and k = 100; after mean centering) for four PLMs (Ankh, ESM2, ProstT5, ProtT5) as an increasing fraction of features is randomly permuted (x-axis). The dashed horizontal line indicates the expected random baseline score (0.85), reflecting the alignment score that would be obtained under fully randomized feature given the class distribution. Error bars indicate variability across 5 repeated permutations. All models' scores decay toward the random baseline as permutation increases, confirming that the KNN feature alignment score captures the alignment of the embedding space with the feature rather than reflecting a property of the class distribution alone.

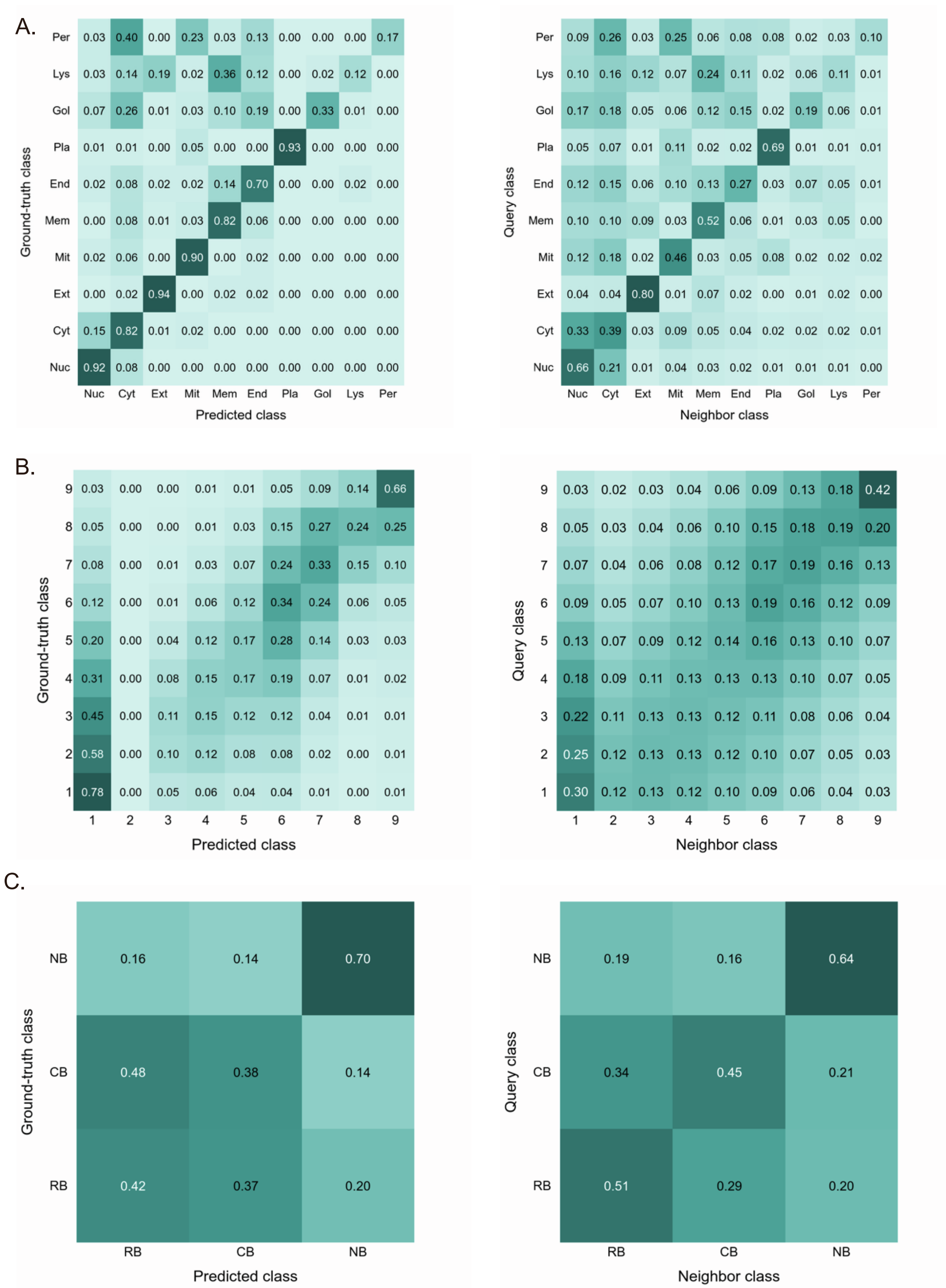

Figure S4: Fractional class distributions from transfer learning and embedding space neighborhood analysis across three tasks.

For each task, the left panel shows the predicted class distribution from the transfer learning model, where values indicate the fraction of predictions falling in each predicted class for each ground-truth class (x-axis: predicted class, y-axis: ground-truth class). The right panel shows the normalized KNN class mixing matrix, where values indicate the proportion of k-nearest neighbors belonging to each neighbor class out of all neighbors of the query class (x-axis: neighbor class, y-axis: query class), normalized per query class row, based on k = 100, cosine distance, after mean centering. **(A) Subcellular localization.** ProtT5 embedding space. Transfer learning performance as reported by Stärk et al. [S2]. Cyt: Cytoplasm; End: Endoplasmic reticulum; Ext: Extracellular; Gol: Golgi apparatus; Lys: Lysosome/vacuole; Mem: Cell membrane; Mit: Mitochondrion; Nuc: Nucleus; Per: Peroxisome; Pla: Plastid. **(B) Residue conservation.** ProtT5 embedding space. Transfer learning performance as reported by Marquet et al. [S4]. Conservation categories 1–9 range from highly variable to highly conserved. **(C) Ligand binding site (three-class).** ProstT5 embedding space, selected as the best-performing embedding space for this task. Transfer learning performance from the MLP classifier trained in this study. NB: non-binding; RB: regular binding; CB: cryptic binding.

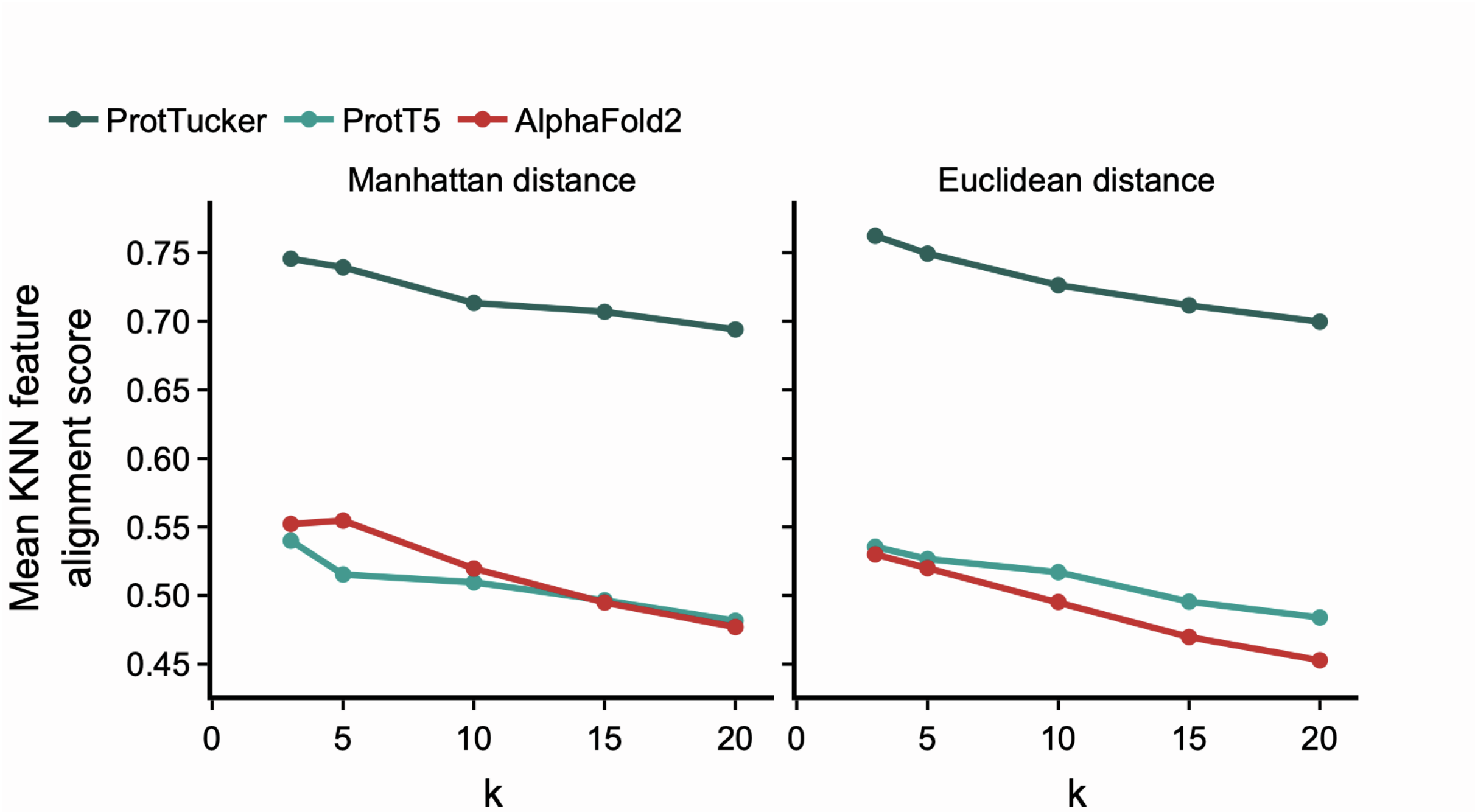

Figure S5: Mean KNN feature alignment scores for the CATH class feature across Manhattan and Euclidean distance metrics.
Mean KNN feature alignment scores (y-axis) for CATH class feature across varying neighborhood sizes (k, x-axis) for ProtTucker, ProtT5, and AlphaFold2, shown for Manhattan (left panel) and Euclidean distance (right panel) after mean centering. ProtTucker consistently ranks highest across both distance metrics, confirming that the ranking pattern observed with cosine distance in Figure 5A is robust to the choice of distance metric.

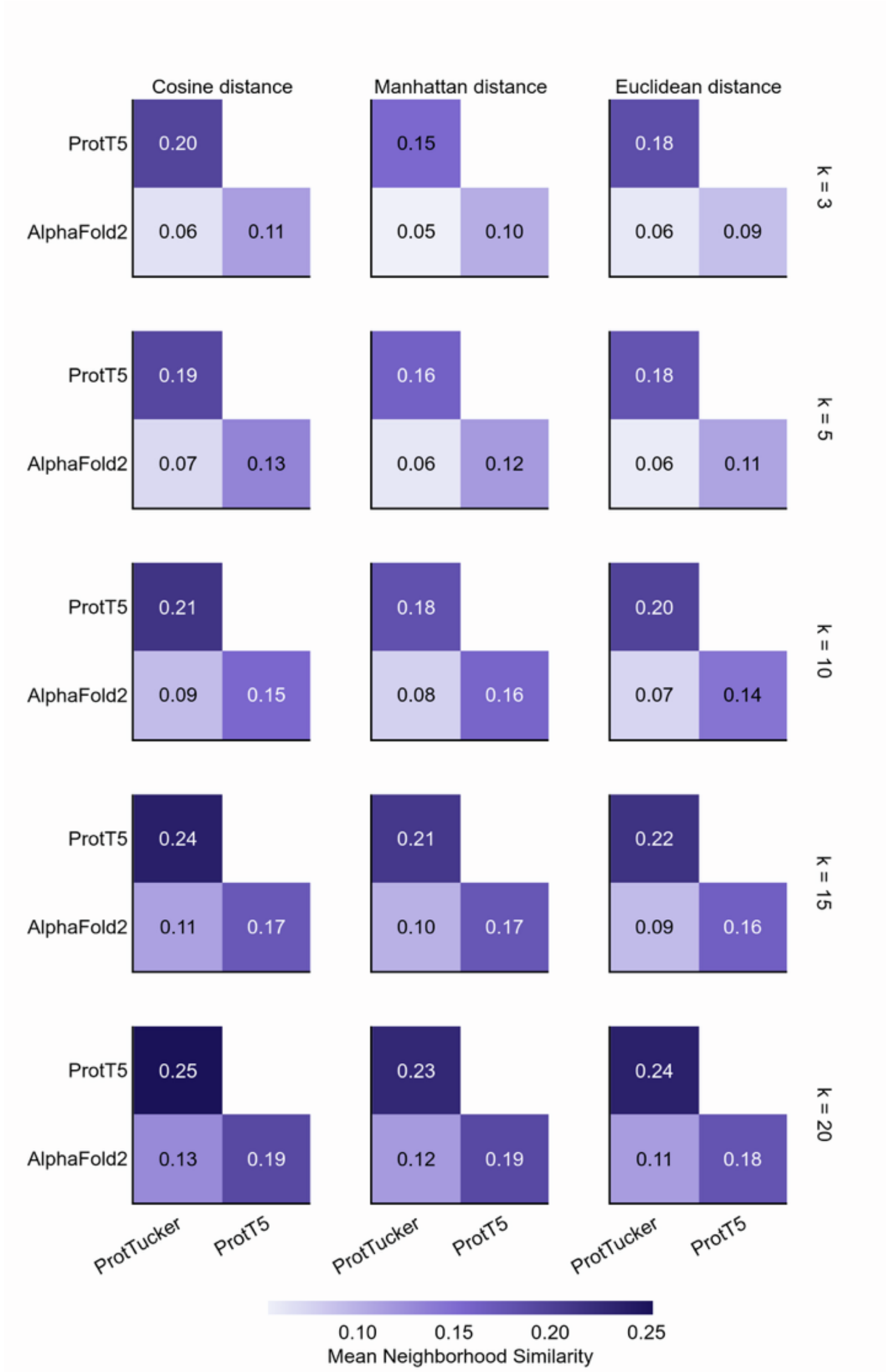

Figure S6: Mean cross-space neighborhood similarity matrices between ProtTucker, ProtT5, and AlphaFold2 across k values and distance metrics.
Mean cross-space neighborhood similarity between all pairs of embedding models (ProtTucker, ProtT5, AlphaFold2), shown as 2×2 matrices for five k values (k = 3, 5, 10, 15, 20; rows) and three distance metrics (cosine, Manhattan, Euclidean; columns). Each cell shows the mean neighborhood similarity for the corresponding model pair at that k value and distance metric; diagonal cells are excluded as self-similarity is uninformative. Across all k values and distance metrics, the ProtTucker–ProtT5 pair consistently shows the highest neighborhood similarity, reflecting the preservation of core representational geometry following contrastive fine-tuning.

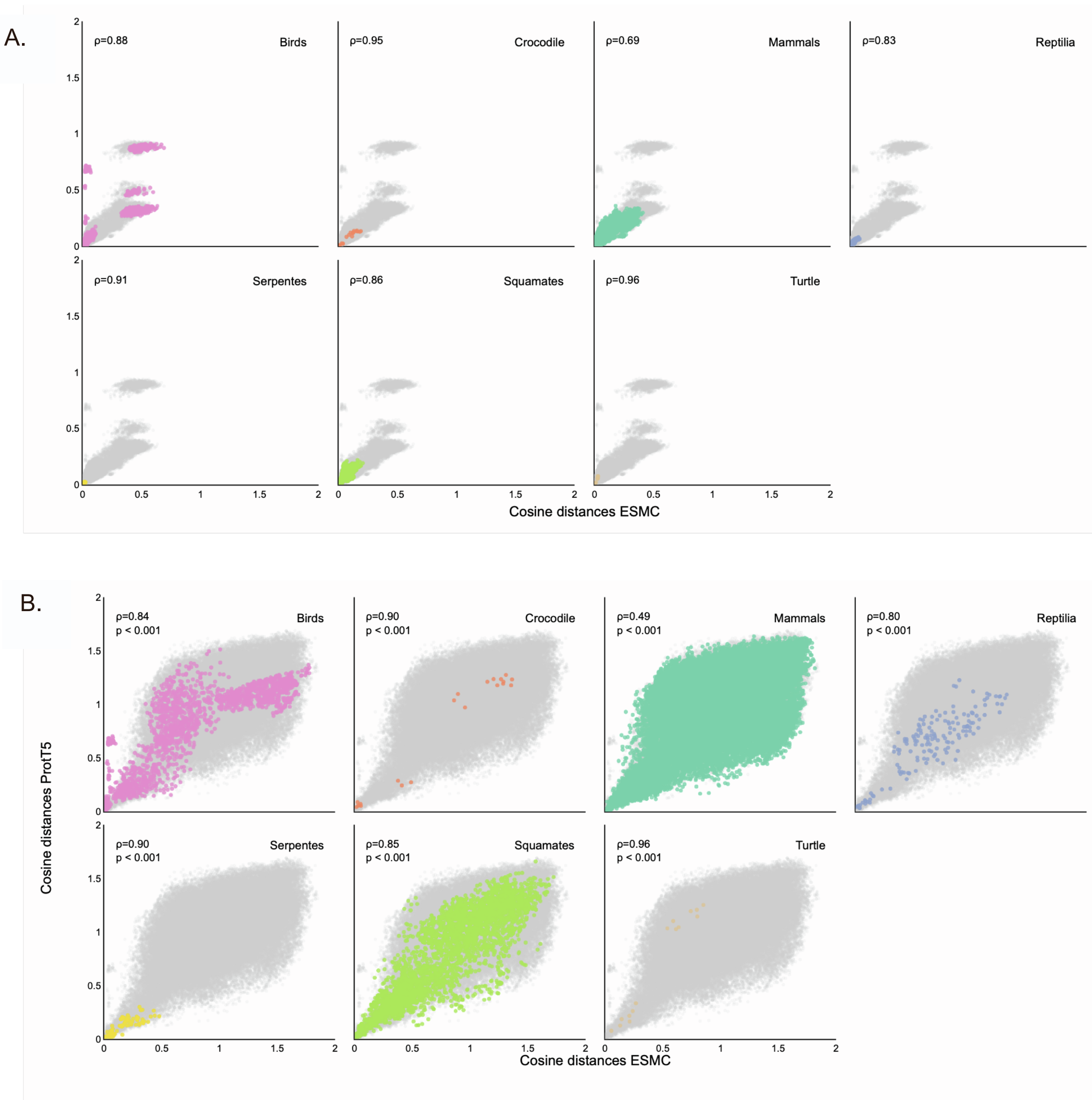

Figure S7: Pairwise cosine distances between PLA2G2 protein embeddings in ESM C and ProtT5, colored by species, before and after mean centering.

Pairwise cosine distances between PLA2G2 protein embeddings in ESM C (x-axis) and ProtT5 (y-axis) for all pairs (grey) and for pairs belonging to the same species (colored points; one panel per species). (A) Before mean centering. A visually distinct divergent cluster is apparent, predominantly composed of pairs from bird species (pink), suggesting a species-level representational difference between the two models. (B) After mean centering. The bird protein cluster is less apparent, indicating that the divergence observed prior to correction is a geometric artifact of anisotropy rather than a genuine representational difference between the models.

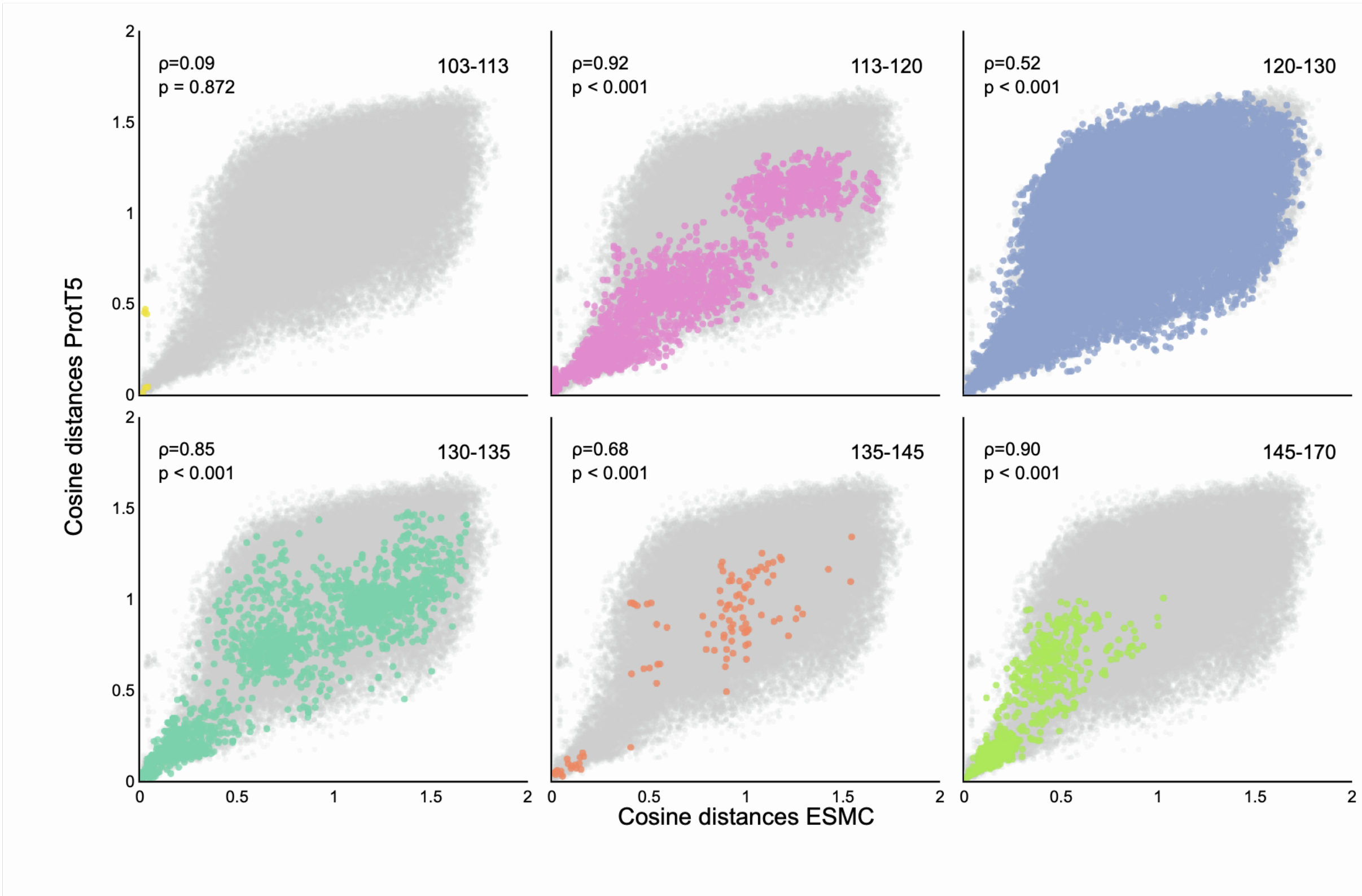

Figure S8: Pairwise cosine distances after mean centering between PLA2G2 protein embeddings in ESM C and ProtT5, colored by binned sequence length.
Pairwise cosine distances after mean centering between PLA2G2 protein embeddings in ESM C (x-axis) and ProtT5 (y-axis) for all pairs (grey) and for pairs falling within the same sequence length bin (colored points; one panel per bin).

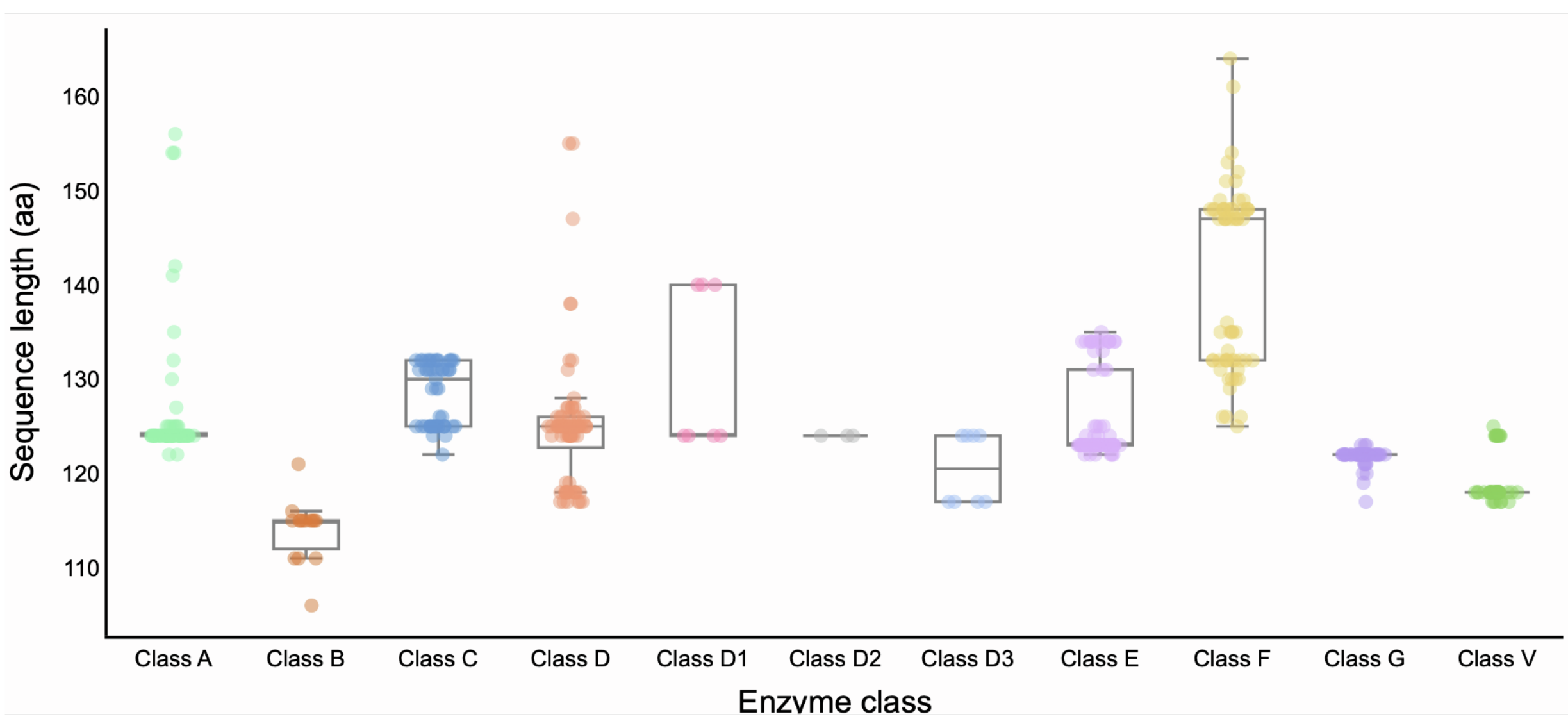

Figure S9: Sequence length distribution per PLA2G2 enzyme class.
Box plots showing the distribution of protein sequence length (y-axis) across PLA2G2 enzyme classes (x-axis).

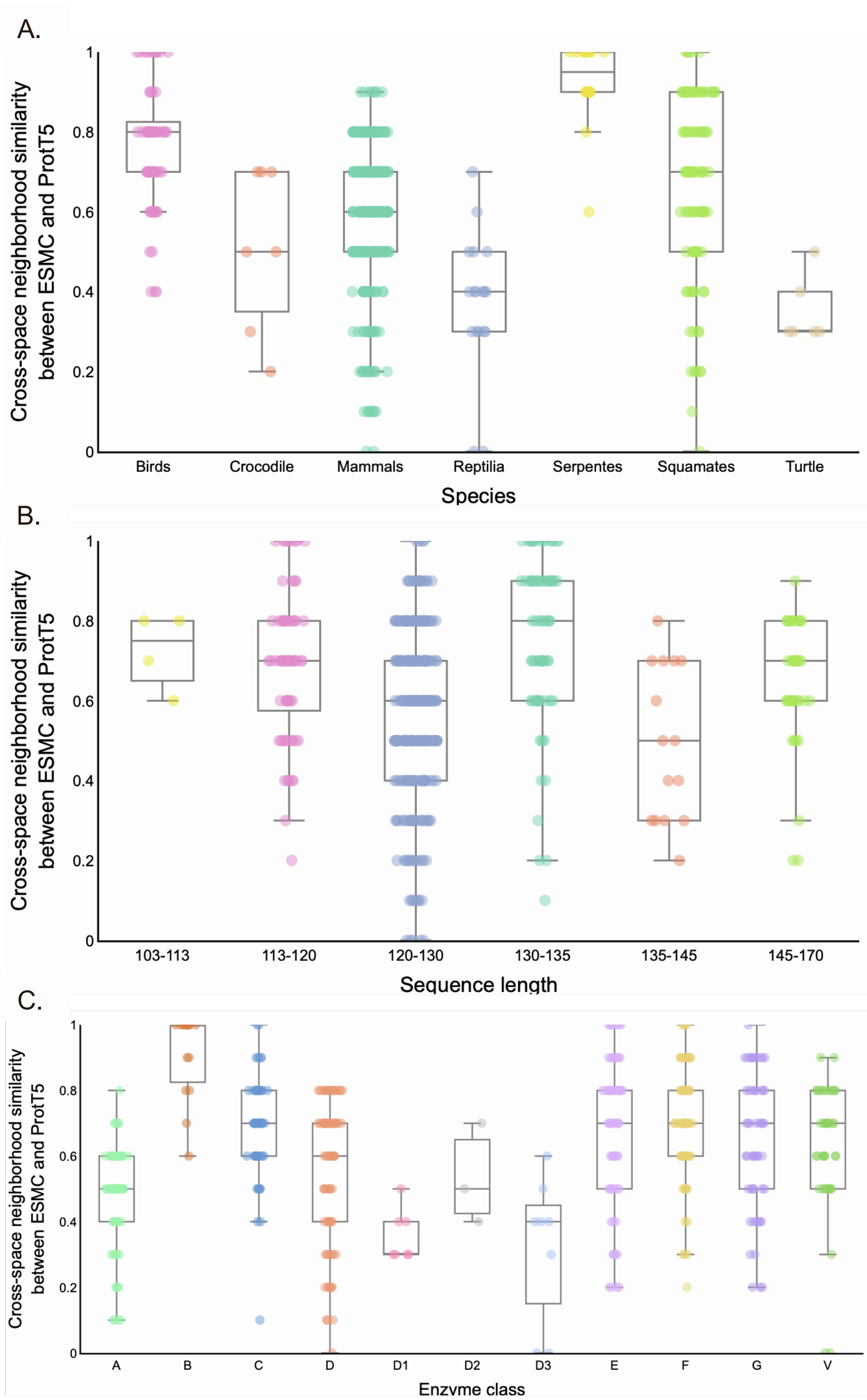

Figure S10: Cross-space neighborhood similarity between ESM C and ProtT5 embeddings of PLA2G2 proteins, stratified by species, sequence length, and enzyme class.
Cross-space neighborhood similarity (y-axis; cosine distance with k = 10) between ESM C and ProtT5 embeddings after mean centering for individual PLA2G2 proteins, shown as box plots with overlaid individual data points, stratified by (A) taxonomic group (Birds, Crocodile, Mammals, Reptilia, Serpentes, Squamates, Turtle), (B) sequence length bin, and (C) enzyme class.

Supplemental Tables

Table S1: Protein counts per subcellular localization class in the subcellular localization dataset

**Class**	**Protein count**	**Percentage**
Nucleus	3,235	28.8%
Cytoplasm	2,180	19.4%
Extracellular	1,580	14.1%
Mitochondrion	1,208	10.8%
Membrane	1,067	9.5%
Endoplasmic reticulum	689	6.1%
Plastid	605	5.4%
Golgi apparatus	286	2.6%
Lysosome/ Vacuole	257	2.3%
Peroxisome	124	1.1%
Total	11,231	

Table S2: Residue counts per conservation class in the residue conservation dataset

**Class**	**Residue count**	**Percentage**
1-conservation	311,365	13.0%
2-conservation	160,915	6.7%
3-conservation	205,712	8.6%
4-conservation	246,379	10.3%
5-conservation	283,649	11.8%
6-conservation	334,004	13.9%
7-conservation	315,853	13.2%
8-conservation	266,929	11.1%
9-conservation	273,931	11.4%
**Total**	**2,398,737**	

Table S3: Residue counts per residue binding class in the residue binding (binary) dataset

**Class**	**Residue count**	**Percentage**
Non-binding	156,687	91.8%
Binding	13,999	8.2%
**Total**	**170,686**	

Table S4: Protein counts per CATH class in the protein structure (CATH) dataset

Class	Protein count	Percentage
A-1	93	31.0%
A-2	77	25.7%
A-3	123	41.0%
A-4	6	2.0%
A-6	1	0.3%
**Total**	**300**	

Table S5: Protein counts per class in the Phospholipase A2 family (PLA2G2) dataset

Feature	Class	Protein count	Percentage
Species	mammals	250	56.1%
	squamates	88	19.7%
	birds	61	13.7%
	reptilia	18	4.0%
	serpentes	16	3.6%
	crocodile	7	1.6%
	turtle	6	1.3%
Enzyme class	D	73	16.4%
	E	70	15.7%
	A	65	14.6%
	F	61	13.7%
	G	53	11.9%
	C	51	11.4%
	V	40	9.0%
	B	15	3.4%
	D3	8	1.8%
	D1	7	1.6%
	D2	3	0.7%
Sequence length	120-130	257	57.6%
	130-135	67	15.0%
	113-120	65	14.6%
	145-170	38	8.5%
	135-145	15	3.4%
	103-113	4	0.9%
Total		446	

Table S6: Residue counts per ligand binding class in the ligand binding site (three-class) dataset

**Dataset**	**Class**	**Residue count**	**Percentage**
Train	Non-binding	12,964	33.3%
	Cryptic binding	12,964	33.3%
	Regular binding	12,970	33.3%
	**Total**	**38,898**	
Test	Non-binding	3,099	33.2%
	Cryptic binding	3,099	33.1%
	Regular binding	3,138	33.6%
	**Total**	**9,336**	

Table S7: Overview of protein language model benchmark tasks, datasets, and reference

Task	Task description	Prediction level	Number of classes	Training and test datasets	Redundancy reduction	Model architecture	Performance measure
Subcellular localization	Predicting the subcellular localization of a protein	Per protein	10	train: DeepLoc training data [S1]; test: setHARD [S2]	Test set was constructed independently from a different data source than the training data and filtered at 20% sequence identity to remove all sequences homologous to training set proteins	Light-attention [S2]	Q10: 10-state per-protein accuracy
Residue conservation	Predicting the conservation of a protein residue	Per residue	9 (1 = highly variable to 9 = highly conserved)	train and test: ConSurf10k [S3, S4]	Redundancy reduction at 25% sequence identity was applied to the full dataset prior to random partitioning into train, validation, and test sets	2-layer CNN [S4]	Q9: nine-state per-residue accuracy
Residue binding (binary)	Predicting binding of a protein to ligands (small molecule, metal ion, or nucleotide)	Per residue	2 (binding vs non-binding)	BioLip [S5]; train and test splits as in [S6]	Redundancy reduction at less than 20% pairwise sequence identity over 250 aligned residues was applied to the full dataset prior to splitting into development and test sets	2-layer CNN [S6]	F1 score
Ligand binding site (three-class)	Predicting cryptic binding residues, regular binding residues and non-binding residues	Per residue	3	Train: scPDB-enhanced [S7], CryptoBench train set [S8]; Test: LIGYSIS [S9, S10], CryptoBench test set [S8]	Controlled sequence similarity down to 10%	3-layer MLP	AUPRC

CNN: convolutional neural network; MLP: multilayer perceptron

Table S8: Performance of transfer learning model for ligand binding site (three-class)

Model	Accuracy	AUC	MCC	F1	AUPRC
Ankh	0.486	0.727	0.231	0.484	0.559
ProtT5	0.486	0.733	0.234	0.486	0.565
ProstT5	0.499	0.739	0.249	0.497	0.585
ESM2	0.448	0.677	0.178	0.425	0.500

Table S9: Embedding space geometry diagnostics for all model-dataset combinations

Dataset	Model	AvgCosine (raw)	AvgCosine (centered)	Robin Hood score (raw)	Robin Hood score (centered)	N90 Dim	Mean centering applied
Subcellular localization	Ankh	0.437	0.003	0.289	0.257	1224 out of 1536	True
	ESM2	0.956	0.005	0.389	0.326	1983 out of 2560	True
	ProstT5	0.330	0.001	0.258	0.248	859 out of 1024	True
	ProtT5	0.663	0.009	0.270	0.270	799 out of 1024	True
Residue conservation	Ankh	0.027	0.001	0.330	0.324	1247 out of 1536	True
	ESM2	0.689	0.003	0.958	0.494	2203 out of 2560	True
	ProstT5	0.037	-0.001	0.292	0.290	825 out of 1024	True
	ProtT5	0.015	-0.000	0.296	0.296	868 out of 1024	True
Residue binding (binary)	Ankh	0.026	0.000	0.264	0.258	1248 out of 1536	True
	ESM2	0.717	0.002	0.560	0.268	2201 out of 2560	True
	ProstT5	0.041	0.001	0.255	0.250	824 out of 1024	True
	ProtT5	0.016	0.000	0.224	0.222	864 out of 1024	True
Ligand binding site (three-class)	Ankh	0.038	0.000	0.265	0.254	1242 out of 1536	True
	ESM2*	0.356	0.003	0.479	0.393	1086 out of 1280	True
	ProstT5	0.045	0.001	0.231	0.228	828 out of 1024	True
	ProtT5	0.021	0.001	0.233	0.228	861 out of 1024	True
CATH	ProtT5	0.278	0.003	0.310	0.220	864 out of 1024	True
	ProtTucker	0.853	0.001	0.423	0.244	113 out of 128	True
	AlphaFold2	0.975	0.011	0.344	0.267	189 out of 256	True
PLA2G2	ESM C	0.920	0.021	0.252	0.264	574 out of 960	True
	ProtT5	0.894	0.005	0.270	0.264	704 out of 1024	True

AvgCosine (raw) = average pairwise cosine similarity before mean-centering; AvgCosine (centered) = average pairwise cosine similarity after mean-centering; N90 Dim = number of dimensions capturing 90% of total variance before mean centering; * For ligand binding sites (three-class), a different ESM-2 model version was used: esm2_t33_650M_UR50D. All other tasks used esm2_t36_3B_UR50D.

Table S10: One-sided Wilcoxon signed-rank tests for KNN feature alignment scores at k = 100 for the subcellular localization dataset after mean centering of the embeddings.
P-values shown as 0.000000e+00 are smaller than machine precision (p < 2.22e-16).

Distance metric	Model 1	Model 2	p	Rank biserial correlation
Cosine	ProtT5	Ankh	2.045e-284	0.408
Cosine	ProtT5	ESM2	0.000e+00	0.496
Cosine	Ankh	ESM2	9.259e-11	0.072
Cosine	Ankh	ProstT5	3.047e-235	0.371
Cosine	ESM2	ProstT5	9.710e-85	0.219

Table S11: One-sided Wilcoxon signed-rank tests for KNN feature alignment scores at k = 100 for the residue conservation dataset after mean centering of the embeddings.
P-values shown as 0.000000e+00 are smaller than machine precision (p < 2.22e-16).

Distance metric	Model 1	Model 2	p	Rank biserial correlation
Cosine	Ankh	ESM2	0.000000e+00	0.050
Cosine	ESM2	ProtT5	0.000000e+00	0.228
Cosine	ProtT5	ProstT5	0.000000e+00	0.203

Table S12: One-sided Wilcoxon signed-rank tests for KNN feature alignment scores at k = 100 for the residue binding (binary) dataset after mean centering of the embeddings.

Distance metric	Model 1	Model 2	p	Rank biserial correlation
Cosine	Ankh	ProtT5	4.374e-95	0.063
Cosine	ProtT5	ProstT5	5.731e-174	0.085
Cosine	ProstT5	ESM2	1.154e-05	0.013

Table S13: One-sided Wilcoxon signed-rank tests for KNN feature alignment scores at k = 100 for the ligand binding site (three-class) dataset after mean centering of the embeddings.

Distance metric	Model 1	Model 2	p	Rank biserial correlation
Cosine	ProstT5	ProtT5	1.617e-49	0.088
Cosine	ProtT5	Ankh	1.808e-68	0.104
Cosine	Ankh	ESM2	9.189e-182	0.171

Table S14: One-sided Wilcoxon signed-rank tests for KNN feature alignment scores at k = 20 for the protein structure (CATH) dataset after mean centering of the embeddings.

Distance metric	Model 1	Model 2	p	Rank biserial correlation
Cosine	ProtTucker	AlphaFold2	1.541e-36	0.767
Cosine	ProtTucker	ProtT5	6.471e-37	0.871
Cosine	ProtT5	AlphaFold2	0.0009	0.221

Table S15: One-sided Wilcoxon signed-rank tests for neighborhood similarity scores at k = 20 for the protein structure (CATH) dataset after mean centering of the embeddings.

Distance metric	Model pair 1	Model pair 2	p	Rank biserial correlation
Cosine	ProtTucker to ProtT5	ProtT5 to AlphaFold2	5.353e-09	0.405
Cosine	ProtT5 to AlphaFold2	ProtTucker to AlphaFold2	1.411e-23	0.708

Supplemental Notes

S1: EmmaEmb guided diagnostic workflow for embedding space ranking

Steps 1-2 characterize the geometric properties of the embedding space and are run once per embedding space prior to any feature-specific analysis. Steps 3-6 are run separately for each feature of interest, as class distribution, k range, and robustness assessments depend on the specific feature being analyzed.

A worked example of Step 1 and the full workflow is provided as an interactive Jupyter notebook, viewable and executable in Google Colab within the EmmaEmb github repository. The notebook demonstrates each step on an example dataset and can be adapted for user-provided embedding spaces and metadata.

Step 1: Anisotropy assessment

Tests:

- Compute average pairwise cosine similarity between randomly sampled embedding pairs (following [S11]) on raw and mean-centered embeddings
- Compute per-dimension variance to identify dominant dimensions

Decision:

- if average cosine similarity drops substantially after mean centering → mean-offset anisotropy detected → apply mean centering to all subsequent steps after step 2
- if average cosine similarity remains high after mean centering → structural anisotropy → recommend cosine distance; flag results for caution
- A small number of dimensions account for a disproportionate fraction of total variance → dimension dominance detected:
  - avoid Euclidean and Manhattan distances; distances will be driven by dominant dimensions regardless of biological signal in remaining dimensions
  - cosine distance is more robust but not immune; if dominance persists after mean centering, interpret all distance-based results with caution

Output: processed embeddings (raw or mean-centered); recommended distance metric; dimension variance profile

Step 2: Hubness assessment

Tests:

- Compute k-occurrence distribution: count how many times each point appears as a nearest neighbor of others (following [S12]) and visualize skewness of k-occurrence distribution
- Compute Robin Hood index (RHI): fraction of total occurrences that would need to be redistributed from hubs to equalize the distribution [S13], run at k = 5, 10, 20 to confirm stability of hubness estimate - Compare RHI across models, not against absolute thresholds, a large spread in RHI across models on the same dataset indicates that KNN scores are geometrically less comparable between models, which could distort relative rankings independently of biological signal

Note that hubness tends to increase with embedding dimensionality [S12], a high RHI for a high-dimensional model may partly reflect dimensionality rather than learned representation properties; reporting effective rank or intrinsic dimensionality alongside RHI helps disambiguate

Decisions:

- RHI drops substantially after mean centering, and values are low and comparable across models → hubness was primarily driven by anisotropy and has been addressed by mean centering → no contraindicating measures against proceeding with KNN-based analysis
- RHI drops after mean centering, but one or more models remain substantially more hubbed than others → asymmetric residual hubness remains → flag affected models; rankings for those models should be interpreted with additional caution; cross-model comparisons may not be geometrically equivalent
- RHI remains high and comparable across all models after mean centering → structural or dimensionality-driven hubness → no contraindicating measures against proceeding, but results should be interpreted with caution; prefer smaller k values within the range set in Step 3; note that KNN scores may underestimate true representational differences for all models equally, which preserves relative rankings but reduces absolute interpretability
- RHI is high for some models but not others after mean centering, and the spread is large → asymmetric hubness not resolved by centering → flag strongly; relative embedding space rankings are potentially unreliable; consider whether the analysis question can be reframed to reduce dependence on local neighborhood structure
- RHI is low and comparable across all models on raw embeddings with no substantial change after centering → no contraindicating measures against proceeding

Output: RHI before and after centering per model; cross-model spread; flag indicating whether hubness concern is resolved, partially resolved, or unresolved; recommended k ceiling adjustment if applicable

Step 3: Class distribution and k range

Tests:

- Count samples per class; compute class size distribution
- Compute imbalance ratio (largest class / smallest class)
- Compute expected random baseline alignment score per class: $n_{class} / (N - 1)$, scores should be interpreted relative to this baseline throughout

Determining k value:

- Hard ceiling: $k < n_{min}$ (smallest class size): if $k \geq n_{min}$, alignment scores decrease trivially rather than reflecting embedding structure
- Soft ceiling: $k \leq \sqrt{N}$ as a general heuristic; for datasets where $\sqrt{N}$ approaches n_{min} $k \leq 5\%$ of N
- Tighter ceiling if high hubness detected in Step 2: $k \leq \sqrt{n_{min}}$
- Range construction: rather than testing a single k value, the analysis is run across a progressive range of k values from the lower bound to the upper bound, for example k = 1, 2, 5, 10, 20, 50, 100 or similar geometric progression, so that the stability of rankings across neighborhood scales can be assessed directly. The specific steps within the range are chosen to be informative at both small and large scales rather than uniformly spaced, since neighborhood structure tends to change most rapidly at small k values
- If the ANNOY approximate nearest neighbor algorithm was used: account for the fact that ANNOY returns approximate rather than exact neighbors, which introduces a small degree of noise into the k-occurrence counts and alignment scores. The k range used should be reported alongside the number of trees used in the ANNOY index, and results at very small k values ($k < 5$) should be interpreted with additional caution since approximation errors are proportionally larger relative to the neighborhood size

Flag missing data: columns with a high proportion of missing values are flagged; a default threshold of 70% is used as a practical starting point, which users may adjust depending on their dataset.

Decision:

- datasets with severe class imbalance (a default imbalance ratio of 10 is used as a practical indicator, though the appropriate threshold is context-dependent) → flag as highly imbalanced → Step 5 rebalancing analysis required

Output: k range; expected random baseline per class; imbalance flag; missing data flags

Step 4: Parameter sensitivity analysis

Test:
- Ranking stability plot: Run KNN alignment scores across full k range from Step 3. Run across all three distance metrics (cosine, Euclidean, Manhattan) even if one is recommended, sensitivity across metrics is itself informative; Produce ranking stability plot: model scores (y) vs k (x), one line per model, one panel per metric

Decision:
- Consistent embedding space rankings across full k range and all metrics → high confidence in model prioritization
- Rankings consistent across k but sensitive to distance metric → report cosine results as primary; note metric sensitivity
- Rankings crossing or inverting across k range → conclusions are parameter-sensitive → report full range; do not report single k

Output: ranking stability plot; confidence flag (stable / metric-sensitive / parameter-sensitive)

Step 5: Class imbalance robustness (run if flagged in Step 3)

Test:
- Rebalancing stability plot: Progressive rebalancing: down sample majority class toward n_min in steps; repeat each ratio multiple times for stable estimates; Produce rebalancing stability plot: model scores (y) vs imbalance ratio (x)

Decision:
- Consistent rankings across all subsampling ratios → imbalance is not driving the result → report original rankings with confidence
- Rankings change substantially under rebalancing → original ranking was frequency-driven, not structure-driven → balanced sampling gives more trustworthy estimate; report rebalanced rankings as primary
- Rankings inconsistent across subsampling levels → neighborhood-based model prioritization is not reliable for this dataset → flag; do not report a embedding space ranking

Step 6: Feature noise robustness

Test:
- Noise stability plot: Progressively corrupt a fraction of annotations randomly (0% to 100% in steps); keep embeddings fixed; recompute embedding space rankings at each noise level; produce noise stability plot: embedding space rankings (y) vs noise level (x)
- Report crossing point: the noise level at which embedding space rankings first become unreliable

Decision:
- Rankings are consistent across all noise levels up to the point where annotations are substantially randomized → strong evidence that conclusions are robust to annotation uncertainty; the crossing point can be reported as a lower bound on the annotation quality required for the ranking to hold

- Rankings change at low noise levels → conclusions are sensitive to metadata quality; the crossing point gives a concrete, interpretable statement about required annotation quality: rankings are only reliable if annotation error rate is below the crossing point

References

S1. Almagro Armenteros, J.J., Sønderby, C.K., Sønderby, S.K., Nielsen, H., and Winther, O. (2017). DeepLoc: prediction of protein subcellular localization using deep learning. Bioinformatics *33*, 3387–3395. https://doi.org/10.1093/bioinformatics/btx431.

S2. Stärk, H., Dallago, C., Heinzinger, M., and Rost, B. (2021). Light attention predicts protein location from the language of life. Bioinformatics Advances *1*, vbab035. https://doi.org/10.1093/bioadv/vbab035.

S3. Ben Chorin, A., Masrati, G., Kessel, A., Narunsky, A., Sprinzak, J., Lahav, S., Ashkenazy, H., and Ben-Tal, N. (2020). ConSurf-DB: An accessible repository for the evolutionary conservation patterns of the majority of PDB proteins. Protein Sci *29*, 258–267. https://doi.org/10.1002/pro.3779.

S4. Marquet, C., Heinzinger, M., Olenyi, T., Dallago, C., Erckert, K., Bernhofer, M., Nechaev, D., and Rost, B. (2022). Embeddings from protein language models predict conservation and variant effects. Hum Genet *141*, 1629–1647. https://doi.org/10.1007/s00439-021-02411-y.

S5. Yang, J., Roy, A., and Zhang, Y. (2012). BioLiP: a semi-manually curated database for biologically relevant ligand–protein interactions. Nucleic Acids Research *41*, D1096–D1103. https://doi.org/10.1093/nar/gks966.

S6. Littmann, M., Heinzinger, M., Dallago, C., Weissenow, K., and Rost, B. (2021). Protein embeddings and deep learning predict binding residues for various ligand classes. Sci Rep *11*, 23916. https://doi.org/10.1038/s41598-021-03431-4.

S7. Vít Škrhák (2026). scPDB-Enhanced: An expansion of scPDB including all experimentally observed binding sites. https://doi.org/https://doi.org/10.5281/zenodo.18271517 https://doi.org/https://doi.org/10.5281/zenodo.18271517.

S8. Škrhák, V., Novotný, M., Feidakis, C.P., Kriváк, R., and Hoksza, D. (2025). CryptoBench: cryptic protein–ligand binding sites dataset and benchmark. Bioinformatics *41*, btae745. https://doi.org/10.1093/bioinformatics/btae745.

S9. Utgés, J.S., and Barton, G.J. (2024). Comparative evaluation of methods for the prediction of protein–ligand binding sites. J Cheminform *16*, 126. https://doi.org/10.1186/s13321-024-00923-z.

S10. Utgés, J.S., MacGowan, S.A., and Barton, G.J. (2025). LIGYSIS-web: a resource for the analysis of protein-ligand binding sites. Nucleic Acids Res *53*, W351–W360. https://doi.org/10.1093/nar/gkaf411.

S11. Ethayarajh, K. (2019). How Contextual are Contextualized Word Representations? Comparing the Geometry of BERT, ELMo, and GPT-2 Embeddings. In Proceedings of the 2019 Conference on Empirical Methods in Natural Language Processing and the 9th International Joint Conference on Natural Language Processing (EMNLP-IJCNLP), K. Inui, J. Jiang, V. Ng, and X. Wan, eds. (Association for Computational Linguistics), pp. 55–65. https://doi.org/10.18653/v1/D19-1006.

S12. Radovanovic, M., Nanopoulos, A., and Ivanovic, M. (2010). Hubs in space: Popular nearest neighbors in high-dimensional data. Journal of machine learning research *11*, 2487–2531.

S13. Feldbauer, R., Leodolter, M., Plant, C., and Flexer, A. (2018). Fast Approximate Hubness Reduction for Large High-Dimensional Data. In 2018 IEEE International Conference on Big Knowledge (ICBK), pp. 358–367. https://doi.org/10.1109/ICBK.2018.00055.
